# Immune evasion in cancer: mechanisms and cutting-edge therapeutic approaches

**DOI:** 10.1038/s41392-025-02280-1

**Published:** 2025-07-31

**Authors:** Muhammad Tufail, Can-Hua Jiang, Ning Li

**Affiliations:** 1 Department of Oral and Maxillofacial Surgery, Center of Stomatology, Xiangya Hospital, Central South University, Changsha, China; 2 Institute of Oral Precancerous Lesions, Central South University, Changsha, China; 3 Research Center of Oral and Maxillofacial Tumor, Xiangya Hospital, Central South University, Changsha, China; 4 National Clinical Research Center for Geriatric Disorders, Xiangya Hospital, Central South University, Changsha, China

**Keywords:** Cancer, Cancer therapy

## Abstract

Immune evasion represents a significant challenge in oncology. It allows tumors to evade immune surveillance and destruction, thereby complicating therapeutic interventions and contributing to suboptimal patient outcomes. This review addresses the critical need to understand how cancers evade immune surveillance. It aims to provide a comprehensive overview of strategies of tumors to escape immune detection by examining tumor-induced immune suppression, immune checkpoint regulation, and genetic and epigenetic influences. Moreover, it explores the dynamic role of the tumor microenvironment (TME) in fostering immune resistance and highlights the impact of metabolic reprogramming on immune suppression. Additionally, this review focuses on how tumor heterogeneity influences immune evasion and discusses the limitations of current immunotherapies. The role of key signaling pathways, including programmed cell death protein 1/programmed cell death ligand 1 (PD-1/PD-L1), cytotoxic T-lymphocyte–associated antigen 4 (CTLA-4), transforming growth factor-β (TGF-β), nuclear factor kappa-light-chain-enhancer of activated B cells (NF-κB), and cyclic GMP-AMP synthase-stimulator of interferon genes (cGAS–STING) is analyzed to elucidate their contributions to immune escape. Emphasizing the complexities of immune evasion, this review underscores the importance of personalized approaches and the integration of multi-omics data to combat therapeutic resistance. Furthermore, it discusses novel and emerging therapeutic strategies, such as bispecific antibodies, oncolytic viruses, and nanotechnology-driven immunotherapies, showcasing innovative avenues in cancer treatment. The significance of this review lies in its potential to guide future research and innovations in immunotherapy, ultimately improving patient outcomes and advancing our understanding of cancer immunology.

## Introduction

Cancer remains a significant challenge for modern medicine.^1^ The immune system is essential for recognizing and destroying abnormal cells, maintaining cellular homeostasis, and protecting against malignant changes.^2^ However, cancer cells are using various complex mechanisms to evade immune surveillance, enabling them to grow uncontrollably and ultimately form life-threatening tumors.^3,4^

The immune evasion phenomenon in cancer involves a complex interplay among tumor cells, the TME, and immune cells.^5^ Tumor cells evade immune detection and destruction through various strategies, including altering antigen presentation, creating an immunosuppressive microenvironment, and inhibiting immune cell function.^6,7^ These tactics enable cancer cells to persist and grow despite the immune system’s efforts to eradicate them.

Recent immunology and oncology advances reveal mechanisms of immune evasion, providing new insights into how cancer cells manage to subvert immune responses.^8,9^ New findings have led to treatments targeting immune evasion, including immune checkpoint inhibitors (ICIs), CAR-T cell therapy, and cancer vaccines, offering hope for previously untreatable cancers.^10,11^

This review article provides a complete summary of how cancer cells escape the immune system and the most recent breakthroughs in therapy to address these obstacles. We will look at the essential signaling pathways involved in immune evasion, the role of TME in aiding immune escape, and the novel therapeutic methods that are revolutionizing cancer treatment. Understanding the link between cancer and the immune system allows us to understand better the complexity of immune evasion and the possibility of future medicines to improve cancer treatment.

## Mechanisms of immune evasion in cancer

### Tumor-induced immune suppression

**Fig. 1:**
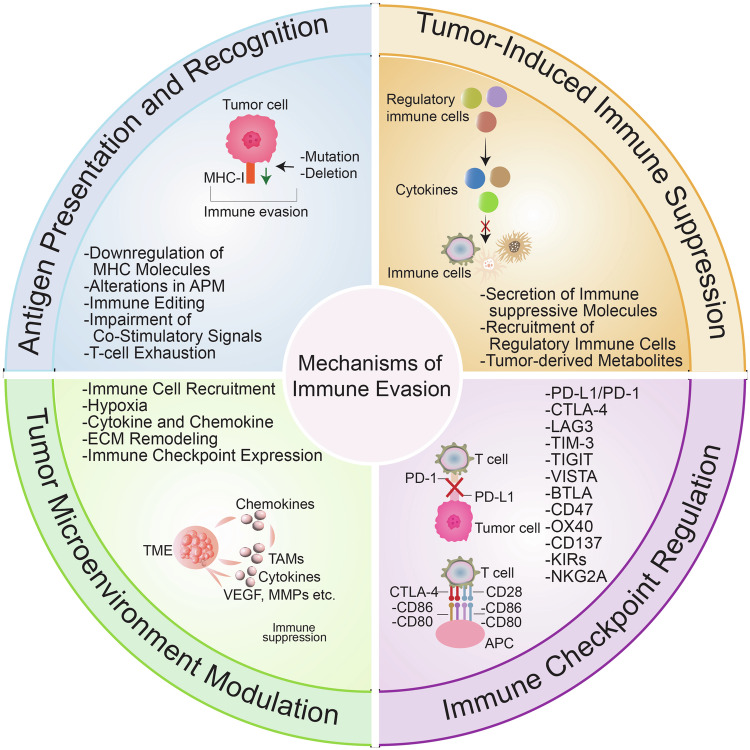
Mechanisms of immune evasion in cancer. This figure illustrates the diverse mechanisms by which cancer cells evade immune surveillance. At the center, the main theme, “Immune Evasion in Cancer,” branches out into four key mechanisms. Tumor-induced immune suppression includes inhibitory cytokines and chemokines, Tregs, and MDSCs, all of which contribute to an immunosuppressive environment. Immune Checkpoint Regulation highlights pathways like PD-1/PD-L1, CTLA-4, and other inhibitory checkpoints, which tumors exploit to deactivate immune cells. In TME modulation, components like hypoxia, metabolic reprogramming, stromal cells, and ECM create physical and biochemical barriers that hinder immune cell infiltration and activation. Antigen presentation and recognition pathways are compromised through MHC class I downregulation, immune editing, etc., reducing the visibility of cancer cells to cytotoxic T cells

Tumor cells avoid the immune system through different mechanisms (Fig. 1). One primary mechanism is tumor-induced immune suppression.^12,13^ This involves forming an immunosuppressive microenvironment that hinders immune cell function and growth, allowing tumor cells to persist and multiply without restriction.^14^ Specifically, tumors can influence the immune system by secreting immunosuppressive chemicals, recruiting regulatory immune cells, and expressing checkpoint molecules that hinder immune responses. The immune system typically identifies and eliminates malignant cells.^15,16^

One of the key mechanisms by which tumors cause immune suppression is the production of soluble substances that impede immune cell activity. For example, tumor cells frequently secrete elevated levels of cytokines, including transforming growth factor-beta (TGF-β),^17^ interleukin-10 (IL-10),^18^ and vascular endothelial growth factor (VEGF),^19,20^ which collectively contribute to an immunosuppressive milieu. Notably, TGF-β is a powerful immunosuppressive cytokine that restricts the activation and growth of T cells^21,22^ and natural killer (NK) cells,^23,24^ both crucial for anti-tumor immunity. Additionally, it also promotes regulatory T cell (Treg) development, further dampening the immune response by suppressing effector T cell activity and fostering immune tolerance.^25,26^ Similarly, IL-10, another immunosuppressive cytokine, plays a vital role in reducing immune responses within the TME. By inhibiting pro-inflammatory cytokine production from macrophages and dendritic cells, IL-10 blocks T cell activation and fosters an anti-inflammatory state.^27,28^ Furthermore, IL-10 can suppress the cytotoxic activity of NK cells^29^ and CD8+ T cells,^30,31^ may further enabling tumor cells to escape immune-mediated destruction. Meanwhile, VEGF, while primarily known for its role in promoting angiogenesis, also has immunosuppressive properties. For example, it can impede dendritic cell maturation,^32,33^ which is required for antigen presentation and T cell activation.^34^ VEGF inhibits dendritic cell function, preventing the initiation of an efficient immune response against the tumor and allowing cancer cells to avoid immune monitoring.

Moreover, tumors actively attract regulatory immune cells, including Tregs and myeloid-derived suppressor cells (MDSCs), which are pivotal in inhibiting anti-tumor-immune responses.^35,36^ Tregs, a CD4+ T cell subset with FoxP3 expression, maintain immune tolerance and prevent autoimmunity.^36^ However, in the TME, Tregs accumulate to suppress effector T cells, NK cells, and other immune cells by releasing IL-10, TGF-β, and expressing CTLA-4.^37^ Similarly, MDSCs, a group of immature myeloid cells, expand in response to tumor factors, suppressing T cells by producing ROS, NO, and arginase, depleting nutrients for T cell function.^38^ Furthermore, MDSCs can encourage the proliferation of Tregs and the production of immune checkpoint markers, further increasing the immunosuppressive milieu within the tumor.^39^

Beyond cytokines and immune cell recruitment, studies have increasingly highlighted the role of tumor-derived metabolites in immune suppression within the TME.^40^ One such metabolite, lactate, has been strongly linked to immune suppression.^41,42^ In fact, in cancer patients, elevated lactate serum levels correlate with tumor burden. Lactic acid inhibits the proliferation and cytokine production of cytotoxic T lymphocytes (CTLs), reducing their cytotoxic activity by up to 50%. Interestingly, CTL function can be restored after a 24-h recovery period in lactic acid–free medium. Tumor spheroids that produce lactic acid also impair CTL function. However, blocking lactate production in these spheroids prevents this effect. T cells rely on glycolysis and efficient lactate export for proper function. Disruption of lactate export by monocarboxylate transporter-1 (MCT-1) further impairs CTL activity.^43^ Tumors often rely on aerobic glycolysis that leads to the accumulation of lactic acid. This byproduct lowers the pH of the TME, creating an acidic environment that directly inhibits the function of immune cells, including T cells, macrophages, dendritic cells, and NK cells. The acidic conditions impair T cell activation and proliferation by disrupting key signaling pathways.^44^ Ex vivo studies have shown that low pH in the TME directly inhibits melanoma tumor-infiltrating lymphocytes (TILs) by reducing their proliferation, activation markers like p-STAT5 and p-ERK, and the production of cytokines such as IL-2, TNFα, and IFN-γ. Treatment with proton pump inhibitors, which increased the intratumoral pH from 6.5 to 7, enhanced the efficacy of adoptive cell therapy.^45^ Similarly, mice drinking bicarbonate showed reduced tumor volume and increased CD8+ T cell infiltration, improving both ACT and immune checkpoint blockade efficacy.^46^ Furthermore, inhibition of carbonic anhydrase IX, which modulates pH, also boosted responses to ICB.^47^ In a model of Myc-overexpressing lymphomas, which typically produce fewer IFN-γ + NK cells, providing exogenous bicarbonate reversed the acidic TME, increased NK cell infiltration, and enhanced their activity, including JNK phosphorylation and IFN-γ production. These changes led to improved mouse survival.^48^ Overall, it suggests that neutralizing the acidic TME, which is driven by increased glycolysis, can enhance the functionality and activity of anti-tumor immune cells.

In addition to affecting T cells, lactic acid can reprogram other immune cells. For example, it induces macrophages to adopt an immunosuppressive M2 phenotype, promoting the secretion of anti-inflammatory cytokines like IL-10 and TGF-β. This shift suppresses anti-tumor immunity and fosters tumor growth.^49,50^ Dendritic cells, crucial for activating T cells, are also impaired under acidic conditions, which reduces their ability to prime T cells effectively. Lactic acid further complicates immune responses by promoting the expansion of Tregs, which inhibit effector T cells. DEC205, a key endocytotic receptor on dendritic cells, plays an essential role in immune regulation and tumor immunotherapy. Specifically, DEC205 recognizes apoptotic and necrotic cells through a pH-dependent mechanism. At acidic pH, DEC205 adopts a compact double-ringed conformation, allowing it to bind dead cells. At basic pH, the receptor extends, losing its binding ability. Importantly, DEC205 does not recognize healthy cells at either pH, highlighting its selective role in clearing dead cells. This pH-dependent mechanism may provide valuable insights into immune surveillance and tumor scavenging, offering potential for improving immune therapies targeting cell death.^51^

While lactic acid is the most well-known metabolite involved in immune suppression, ammonia is another byproduct that plays a critical role, particularly in cancer. Traditionally regarded as a cytotoxin, ammonia has recently been shown to induce a unique form of cell death in effector T cells. In rapidly proliferating T cells, ammonia is produced through glutaminolysis and is released into the mitochondria before being stored in the lysosomes. When ammonia accumulates excessively, it causes lysosomal alkalization, which disrupts ammonia storage and triggers its reflux into the mitochondria. This cascade results in mitochondrial damage, lysosomal dysfunction, and impaired autophagic flux, leading to T cell death. Blocking glutaminolysis or inhibiting lysosomal alkalization can prevent this form of cell death, improving T cell survival and enhancing the effectiveness of T cell-based cancer immunotherapies.^52^ Therefore, understanding how tumor-derived metabolites shape the TME and suppress immune responses offers new opportunities for cancer therapy. Targeting these metabolic pathways, such as by inhibiting lactic acid production or neutralizing the acidic environment, could restore immune function.

### Immune checkpoint regulation

Immune checkpoint molecules are proteins that modulate immune responses by sending inhibitory signals to prevent overactivation. Tumor cells can also elude immune detection by using immune checkpoint pathways, which are generally involved in self-tolerance and autoimmune control (Fig. 1). Immune checkpoints like PD-L1 and CTLA-4 are overexpressed in tumor and immune cells, inhibiting T cell activation and proliferation, which hampers the anti-tumor response.^53,54^ Tumor cells can upregulate immune checkpoint molecules in response to a variety of circumstances, including oncogenic signaling pathways and the presence of inflammatory cytokines in the TME. For example, stimulation of the PI3K/AKT pathway in tumor cells might result in increased expression of PD-L1,^55,56^ while the presence of IFN-γ can induce PD-L1 expression as a feedback mechanism to limit immune activation.^57^ Such as a previous study discovered that CD8-positive lymphocytes secrete Interferon-γ, which enhances PD-L1 expression in ovarian cancer cells, thereby promoting tumor growth and facilitating immune evasion. The findings suggest that lymphocyte infiltration and the status of IFN-γ are critical factors in assessing the effectiveness of anti-PD-1 or anti-PD-L1 therapies in ovarian cancer.^58^ This dynamic modulation of immunological checkpoints allows malignancies to avoid immune surveillance and maintain an immunosuppressive microenvironment.

Aside from PD-L1/PD-1 and CTLA-4, a few more immune checkpoint molecules are emerging as key actors in cancer immune evasion. One such molecule is lymphocyte-activation gene 3 (LAG-3). LAG-3 is a co-inhibitory receptor that is frequently expressed alongside PD-1 on exhausted T lymphocytes. It attaches to MHC class II molecules and sends inhibitory signals, which can drastically limit T cell proliferation and cytokine production. This process leads to tumor-immune evasion because LAG-3 inhibits the anti-tumor immune response.^59,60^ Dual inhibition of PD-1 and LAG-3 can induce T cell function, beneficial in chronic infections and cancer. The bispecific PD-1×LAG-3 molecule Tebotelimab showed safety and effectiveness in advanced cancer, generating responses in various solid tumors and LAG-3+ non-Hodgkin lymphomas, even with margetuximab.^61^

Similarly, T cell immunoglobulin and mucin-domain containing-3 (TIM-3), an emerging immune checkpoint, binds ligands like galectin-9 and phosphatidylserine to inhibit T cell responses. It’s often upregulated in exhausted T cells and associated with resistance to PD-1 blockade, making it a promising target for combination therapies.^62^ For example, a study found that co-expression of PD-1 and TIM-3 on TILs in head and neck squamous cell carcinoma (HNSCC) correlates with an exhausted immune phenotype and resistance to PD-1 inhibition. TIM-3 elevation due to PD-1 suppression via PI3K/Akt signaling suggests that dual targeting of PD-1 and TIM-3 could enhance cancer immunotherapy effectiveness.^63^ Another study found that adaptive resistance to PD-1 inhibition in lung cancer is linked to alternative immune checkpoint activation, particularly TIM-3. Adding TIM-3 blocking after PD-1 therapy failure offers survival benefits, indicating TIM-3 as a targetable biomarker against resistance to immune checkpoint inhibitors.^64^

Another key player, T cell immunoreceptor with Ig and ITIM domains (TIGIT), is another emerging immune checkpoint. It is an inhibitory receptor expressed on T cells, NK cells, and other immune cells like dendritic cells and regulatory T cells. It functions as a critical immune checkpoint, particularly in the TME. TIGIT competes with CD226 (DNAM-1) for binding to the common ligands CD112 and CD155. When TIGIT binds to CD155, it transmits an inhibitory signal through the immunoreceptor tyrosine-based inhibition motif (ITIM) in its cytoplasmic domain. This suppresses T cell activation, proliferation, cytokine production, and the killing potential of T cells and NK cells. As a result, TIGIT contributes to immune evasion by tumors.^65,66^ TIGIT’s expression is often upregulated in cancers, contributing to immune evasion. For example, recent studies reported that TIGIT promotes immune evasion by inhibiting the activation and function of NK and T cells, thereby contributing to disease progression and immune escape in cancers such as myelodysplastic syndrome (MDS) and pancreatic cancer. In MDS, high expression of TIGIT on NK and T cells correlates with decreased secretion of activation factors like CD107a, IFN-γ, and TNF-α, while blocking TIGIT enhances anti-tumor immunity.^67^ In pancreatic cancer, the CD155/TIGIT axis is co-opted to maintain immune evasion, as TIGIT modulates T cell exhaustion and dysfunction, with co-blockade strategies showing promise in reinvigorating tumor-reactive T cells.^68^ The presence of TIGIT on immune cells correlates with increased tumor aggressiveness and poor patient prognosis, making it a promising target for immunotherapy. Blocking TIGIT with monoclonal antibodies has emerged as a promising strategy to restore anti-tumor immunity. By reactivating T cells and NK cells, TIGIT inhibitors may improve cancer treatment.^69,70^ These inhibitors are often studied in combination with other immune checkpoint inhibitors to enhance therapeutic efficacy.^71^

In addition, V domain immunoglobulin suppressor of T cell activation (VISTA) is an immune checkpoint molecule that acts as a negative regulator of T cell activation and immune responses. It is expressed on myeloid cells, including macrophages and dendritic cells, as well as T cells. Although its ligands are not fully identified, VISTA plays a key role in regulating immune responses in cancer. When expressed on APCs or T cells, VISTA suppresses T cell activation and proliferation.^72,73^ It functions similarly to other immune checkpoints like PD-1/PD-L1 and CTLA-4, promoting immune tolerance and preventing overactive immune responses.

In the TME, VISTA expression is often elevated, contributing to immune evasion by tumors. The VISTA pathway helps tumors escape immune detection by suppressing T cell activation.^74^ It also promotes the expansion of Tregs, which further suppresses anti-tumor immune responses. VISTA’s role in cancer appears to be context-dependent. For example, in non-small cell lung cancer (NSCLC), VISTA expression is elevated in the TME and is associated with increased immune suppression and resistance to checkpoint blockade therapy, highlighting its role in promoting tumor immune evasion and disease progression.^75,76^ In contrast, in colorectal cancer (CRC) and TNBC, high VISTA expression on tumor-infiltrating immune cells is associated with early-stage disease, favorable prognosis, and enhanced anti-tumor immunity.^77,78^ Given its role in immune suppression, VISTA is being explored as a therapeutic target. Inhibiting VISTA could potentially enhance T cell responses against tumors.^79^

Another important immune checkpoint receptor is B and T lymphocyte attenuator (BTLA). It is found on T cells, B cells, and other immune cells. It binds to HVEM, a receptor on APCs. This interaction inhibits T cell activation and reduces immune responses. BTLA transmits signals that limit T cell expansion and cytokine production, helping maintain immune balance and prevent autoimmunity. The BTLA-HVEM pathway also plays a key role in immune tolerance, particularly in tumors.^80^ In tumors, BTLA expression can contribute to immune evasion. By inhibiting T cell activation, tumor cells can escape detection and destruction by the immune system. This suppression of anti-tumor immunity is one way that cancer cells can evade immune surveillance. For example, in chronic lymphocytic leukemia, elevated BTLA expression on CD4+ and CD8+ T cells is associated with impaired T cell-mediated anti-tumor responses, leading to immune exhaustion. High BTLA levels on T lymphocytes correlate with poor clinical outcomes, such as a shortened time to treatment initiation, and contribute to reduced cytokine production, including IL-2 and IFN-γ, upon T cell activation. This dysregulation is further exacerbated by the co-expression of other inhibitory receptors. In cancer models, blocking BTLA in combination with other immune checkpoint inhibitors, such as PD-1 or bispecific antibodies, enhances T cell-mediated tumor eradication, suggesting a promising therapeutic strategy.^81,82^ Therefore, targeting BTLA may enhance T cell activation and restore anti-tumor immune responses. Such therapies could be beneficial in overcoming immune evasion mechanisms in tumors. Such as in the context of paclitaxel treatment, dual blockade of BTLA and PD-1 has been shown to improve the efficacy of chemotherapy in disseminated intraperitoneal tumors, underscoring the potential of targeting BTLA to overcome immune suppression.^83^

CD47 is known as the “don’t eat me” signal. It is expressed in many cells, including tumor cells. CD47 interacts with SIRPα, a receptor found on macrophages, to inhibit phagocytosis. This prevents immune cells from engulfing and destroying the cell. The binding of CD47 to SIRPα transmits a “self-recognition” signal, preventing macrophages from attacking the cell.^84,85^ Tumor cells overexpress CD47 to evade immune detection and avoid destruction by phagocytes, particularly macrophages. In tumors, CD47 overexpression contributes to immune evasion by shielding cancer cells from immune surveillance. This helps tumor cells escape clearance by the innate immune system, promoting tumor growth and progression. For example, in CRC, CD47 expression is upregulated and is linked to poor prognosis. CD47 promotes tumor cell growth and metastasis by stabilizing the glycolytic enzyme ENO1. This stabilization enhances aerobic glycolysis and activates the ERK signaling pathway. These immune-independent functions of CD47 contribute to CRC progression, making it a potential therapeutic target.^86^ In lung cancer, CD47 is found not only on the surface of cancer cells but also in extracellular vesicles. This form of CD47 inhibits the phagocytic activity of alveolar macrophages, which is critical for immune surveillance. Elevated soluble CD47 in the serum of lung cancer patients further supports immune evasion. It reduces tumor-infiltrating macrophages, promoting an immune-suppressive environment. This highlights the importance of CD47 in early-stage lung cancer progression.^87^ Monoclonal antibodies that block the CD47-SIRPα interaction are being investigated as a promising cancer treatment.^88,89^ By blocking CD47, these antibodies can restore macrophage-mediated phagocytosis of tumor cells, thus enhancing the anti-tumor immune response.

Moreover, OX40 is another key immune checkpoint.^90^ It is a co-stimulatory molecule expressed on activated T cells. It is part of the TNF receptor superfamily and plays a crucial role in promoting T cell expansion, survival, and effector function. Its ligand, OX40L, is typically expressed on APCs. When OX40 interacts with OX40L, it delivers a positive signal that enhances T cell activation. This interaction promotes the survival and proliferation of T cells, boosting their ability to eliminate tumor cells. OX40 signaling also helps sustain T cell effector functions, contributing to a more robust immune response.^91^

In the TME, OX40 expression on T cells can be suppressed, limiting the anti-tumor immune response. However, activating the OX40-OX40L pathway can increase the effectiveness of anti-tumor immunity. This activation may lead to enhanced T cell responses, helping to control tumor growth. For example, OX40 triggering has emerged as a potent strategy to overcome the inhibitory effects of Treg cells in cancer immunotherapy. Activation of OX40 can functionally inactivate Treg cells, thereby enhancing anti-tumor immunity. In tumors, most CD4+ T cells express both Foxp3 and high levels of OX40. Treatment with an agonist anti-OX40 antibody (OX86) induces tumor rejection in 80% of mice, an effect dependent on CD8+ T cells. This response is accompanied by increased infiltration of dendritic cells into draining lymph nodes, which in turn activate tumor-specific cytotoxic T lymphocytes. OX40 triggering also helps overcome tumor-induced immune tolerization, making it a promising approach for enhancing cancer immunotherapy.^92^ OX40 agonists, which stimulate the OX40-OX40L pathway, are being studied as potential cancer therapies. These agonists can enhance T cell responses and improve anti-tumor immunity, particularly when combined with other immune checkpoint inhibitors.^93,94^

4-1BB (CD137) is another important co-stimulatory immune checkpoint protein that enhances T cell activation and anti-tumor immunity. It belongs to the TNF receptor superfamily and plays a key role in T cell activation, survival, and proliferation. Its ligand, 4-1BBL, is found on APCs and other immune cells. When 4-1BB binds to 4-1BBL, it enhances T cell activation, promotes expansion, and supports effector T cell survival. This interaction is crucial for sustaining immune responses, especially in tumors.^95^ In tumors, 4-1BBL expression enhances the immune responses. Activating 4-1BB improves the anti-tumor immune response by boosting T cell expansion and activity, which is important for tumor clearance. For example, a recent study reported that 4-1BB expression was increased in both peripheral blood mononuclear cells (PBMCs) and TILs. The highest levels were found in oropharyngeal cancer, compared to other cancer types. This upregulation of 4-1BB was associated with greater lymphocyte infiltration, including CD8+ and CD4+ T cells, into the TME.^96^ 4-1BB agonists are being explored as cancer immunotherapies. These agonists can stimulate T cell responses, improve tumor eradication, and enhance the effects of other immune checkpoint inhibitors. For instance, targeting 4-1BB with antibodies has been shown to enhance T cell proliferation and survival, promote CD8+ T cell-dependent anti-tumor immunity, and facilitate tumor rejection. Anti-4-1BB antibodies can deplete Treg cells in the TME and promote effector T cell activation, leading to improved anti-tumor responses. Optimizing antibody isotypes and FcγR interactions has further enhanced these effects, making 4-1BB a promising target for cancer immunotherapy.^97^

Killer immunoglobulin-like receptors (KIRs) are another important immune checkpoint that regulate NK cell activity by interacting with MHC class I molecules to modulate immune responses, but tumors can exploit this mechanism to evade NK cell surveillance.^98^ In the TME, KIRs can suppress NK cell activity, which allows cancer cells to evade immune detection and destruction.^99^ Targeting KIRs or blocking their interaction with MHC Class I molecules may restore NK cell function and enhance anti-tumor immunity. KIR inhibitors are being studied as potential cancer treatments. These inhibitors aim to unleash NK cell responses, enabling them to target and destroy tumor cells more effectively. For example, targeting KIRs with monoclonal antibodies has shown promise in enhancing NK cell-mediated anti-tumor responses.^100^ Clinical studies have demonstrated that blocking KIRs can improve NK cell activation, increase tumor cell lysis, and potentially enhance the effects of other immune therapies.^101^ However, while KIR blockade has shown some efficacy, its impact on long-term outcomes, such as leukemia-free survival in AML patients, remains variable and requires further optimization in future trials.^102^

Similarly, research has shown that natural killer group 2 member A (NKG2A) is also a critical immune checkpoint that regulates immune responses. It is an inhibitory receptor on NK and T cells that binds to HLA-E on target cells, suppressing NK cell activation and contributing to immune evasion in cancer.^103^ Tumor cells often exploit the NKG2A-HLA-E axis to escape immune detection. By overexpressing HLA-E, tumor cells can suppress NK cell-mediated immune responses. For example, in EBV+ lymphoma patients, the high-affine LMP-1 GGDPHLPTL peptide variant induces the overexpression of HLA-E, which inhibits NKG2A + NK cells and facilitates the spread of EBV-infected tumor cells.^104^ Similarly, circulating tumor cells (CTCs) in pancreatic cancer upregulate HLA-E through platelet-derived RGS18, thereby evading NK cell-mediated immune surveillance by engaging the HLA-E:CD94-NKG2A immune checkpoint.^105^ Blocking NKG2A or inhibiting its interaction with HLA-E has become a potential therapeutic strategy. Such inhibition could enhance NK cell activity and restore immune surveillance, improving anti-tumor immunity. For example, blocking NKG2A or inhibiting its interaction with HLA-E has emerged as a potential therapeutic strategy in glioblastoma. In this context, the expression of the activating receptor NKG2C on glioblastoma tumor cells was associated with enhanced tumor-resident lymphocyte levels and a stronger anti-tumor response. Furthermore, high NKG2C expression correlated with improved outcomes after PD-1 monoclonal antibody treatment, suggesting that targeting inhibitory NK receptors and promoting NKG2C-mediated responses could restore immune surveillance and improve anti-tumor immunity.^106^

Understanding immune checkpoint regulation has led to the emergence of checkpoint inhibitors, a breakthrough class of cancer immunotherapies. Immune checkpoint inhibitors have revolutionized cancer immunotherapy by restoring T cell activity against tumors. These therapies block inhibitory signals, reactivating immune responses and showing durable efficacy in various cancers. Ongoing research targets additional checkpoints to overcome resistance and enhance treatment outcomes. Combination therapies are also being explored to boost effectiveness by targeting multiple checkpoints simultaneously.

### TME modulation

The TME is a complex ecosystem of cancer cells, stromal cells, immune cells, and signaling molecules.^107^ This microenvironment plays a crucial role in tumor development, metastasis, and immune evasion (Fig. 1).^108^ The TME is not merely a passive setting for tumor growth; instead, it actively shapes the immune response through various mechanisms that collectively promote immune suppression and allow tumor cells to avoid immune surveillance.^108^

The TME recruits and polarizes immune cells into immunosuppressive phenotypes. For example, Tumor-associated macrophages (TAMs), attracted by tumor-derived chemokines like CCL2 and CSF-1, often adopt an M2-like phenotype, producing anti-inflammatory cytokines like IL-10 and TGF-β. TAMs aid immune evasion by suppressing CD8+ T cell and NK cell functions, promoting tissue remodeling and angiogenesis, and supporting tumor growth and metastasis.^109^ M2-TAMs play crucial roles in cancer progression by fostering immune evasion,^109,110^ tissue remodeling,^111^ and tumor growth.^110,112^ They inhibit CD8+ T cell and NK cell functions, enabling tumors to evade immune surveillance. Additionally, M2-TAMs facilitate angiogenesis tissue remodeling, which is vital for tumor expansion and metastasis. For example, M2-TAMs secrete pro-angiogenic factors like VEGF, CXCL8, MMP7, MMP9, and MMP12. This promotes new blood vessel formation to supply nutrients and oxygen to the growing tumor.^111,113^ TAMs interact with endothelial cells and the innate immune system to modulate the TME and promote angiogenesis. The presence of M2-TAMs in the TME plays an important role in generating tumor angiogenesis, which is critical for tumor growth.^114^ TAMs create an immunosuppressive milieu that not only supports tumor cell survival but also enhances their metastatic potential.

Similarly, hypoxia, or reduced oxygen levels, is a prevalent characteristic of TME, resulting from the rapid proliferation of tumor cells and insufficient blood vessel development. Hypoxia stabilizes hypoxia-inducible factors (HIFs), which subsequently upregulates genes associated with angiogenesis, altered metabolism, and immune evasion.^115,116^ For instance, HIF-1α significantly influences the immune landscape in the TME, promoting immune evasion and tumor progression. It binds to the PD-L1 promoter, increasing PD-L1 expression in tumor and immune cells, which contributes to immune exhaustion via PD-1/PD-L1 interactions.^117,118^ Additionally, HIF-1α helps in immune escape by regulating various immune cells.^118^ For example, a recent study shows that microRNAs (miRNAs) are involved in hypoxia-induced treatment resistance in CRC through a feedback loop with HIF-1α, miR-338-5p, and IL-6. HIF-1α suppresses miR-338-5p, activating IL-6-mediated STAT3/Bcl-2 signaling, contributing to resistance. Targeting the HIF-1α/miR-338-5p/IL-6 pathway may enhance CRC sensitivity to oxaliplatin, offering a novel therapeutic strategy against hypoxia-induced drug resistance.^119^ According to another study, in pediatric malignancies like rhabdomyosarcoma (RMS) and Ewing sarcoma (ES), HIF-1α mediates resistance to apoptosis under hypoxic settings. RMS and ES cells resist apoptosis despite pro-apoptotic signals from proteins like p53 and Bcl-2/E1B 19 kDa interacting protein 3. HIF-1α promotes survival by upregulating anti-apoptotic proteins Bcl-2 and cIAP-2, along with glycolytic enzyme GLUT-1. The HIF-1α-induced increase in glucose uptake is crucial for hypoxia-induced apoptosis resistance, suggesting that targeting HIF-1α and its effectors could be a novel therapeutic strategy for pediatric tumors.^120^ Moreover, HIF-1α hinders antigen presentation by downregulating MHC class I molecules and suppressing NKG2D ligands, helping tumor cells evade recognition by CD8+ T cells and NK cells.^121,122^ It recruits and expands MDSCs, which decrease T cell responses through several methods. It also shifts macrophage polarization towards the M2 phenotype, leading to the release of IL-10 and TGF-β, stimulation of angiogenesis, and inhibition of anti-tumor immunity.^123^ These multifaceted roles of HIF-1α highlight its significance in tumor-immune escape and progression.

In addition, metabolic reprogramming within the TME is important for immune evasion.^124,125^ Tumor cells frequently move towards glycolysis, even under normoxic circumstances.^126,127^ This allows them to rapidly proliferate by providing biosynthetic precursors.^128^ As a result of this metabolic reprogramming, tumor cells compete with infiltrating immune cells for key nutrients like glucose and glutamine.^129,130^ This nutrient depletion in the TME impairs the function and differentiation of effector T cells.^130,131^ The increased glycolysis in tumor cells leads to high production of lactate, which is released into the TME.^128^ The resulting acidification of the TME directly inhibits the cytotoxic functions of T cells and NK cells.^124,130^ The metabolic stress in the TME also disrupts the normal metabolic programming of infiltrating immune cells.^129,130^ This metabolic reprogramming of immune cells towards a more suppressive phenotype further enables tumor-immune evasion.^129,130^

Moreover, TME is rich in cytokines and chemokines that influence the immune response, with tumor and stromal cells secreting cytokines that can either stimulate or suppress it. Key cytokines are often overexpressed, reducing effector T cell and NK cell activity while promoting Treg differentiation and proliferation. This creates an immunosuppressive environment that supports tumor growth and evasion. Tregs, influenced by these cytokines, inhibit cytotoxic T lymphocyte and NK cell functions, allowing tumors to escape immune surveillance and proliferate. This immunosuppressive environment significantly impacts the progression of malignancies, highlighting the TME’s critical role in tumor biology and patient outcomes.^132,133^

CCL22 and CCL5 are key chemokines within the tumor TME that participate in immune evasion by recruiting immunosuppressive cells. CCL22 plays a key role in attracting Tregs to the TME, leading to an immunosuppressive environment that favors tumor growth. Neutralizing CCL22 has been shown to significantly reduce Treg migration toward tumor tissues, underscoring its role in immune suppression.^134,135^ Similarly, CCL5 recruits diverse immune cells, including Tregs and MDSCs, and enhances their immunosuppressive functions, thereby promoting tumor progression and resistance to immune-mediated destruction.^136,137^ Recent studies have highlighted the critical role of macrophage-derived cytokines in immune evasion in cancer. For example, according to a recent study, the CCL2-CCR2 signaling axis has been identified as a key mediator of TAM recruitment in esophageal squamous cell carcinoma (ESCC). The accumulation of TAMs, especially those exhibiting M2 polarization, is associated with poor prognosis and enhanced tumor promotion. This occurs through mechanisms such as increased PD-L2 expression, which facilitates immune evasion via the PD-1 signaling pathway.^138^ Similarly, macrophage-derived CCL5, triggered by lipopolysaccharide or a high-cholesterol diet, plays a central role in immune escape in CRC. CCL5 stabilizes PD-L1 expression by promoting the formation of NF-κB p65/STAT3 complexes, which bind to the COP9 signalosome 5 (CSN5) promoters, leading to CSN5 upregulation. CSN5 then regulates the deubiquitination and stabilization of PD-L1, enhancing immune checkpoint inhibition and supporting tumor survival. The high expression of CSN5 in CRC correlates with poor prognosis, suggesting its potential as a therapeutic target.^139^ CCL22 and CCL5 recruit these cells and not only suppress anti-tumor-immune responses but also create a protective niche that shields tumors from immune attacks, highlighting the potential of targeting these chemokines and their receptors to enhance anti-tumor immunity.

The ECM is a crucial structural component of the TME that undergoes significant remodeling during tumor progression. This remodeling is primarily driven by tumor cells and stromal cells, such as cancer-associated fibroblasts (CAFs), which produce enzymes like matrix metalloproteinases (MMPs). These enzymes degrade and restructure the ECM, leading to changes in its composition and stiffness. This can form a physical barrier that inhibits the invasion of immune cells into the tumor core.^140^ This barrier effect limits the immune system’s ability to target and eliminate tumor cells, facilitating immune evasion. Changes in the ECM enhance tumor cell invasion and metastasis while also affecting the overall immune response in the TME, complicating treatment strategies.^141^ For example, targeting the hexosamine biosynthesis pathway (HBP) in pancreatic ductal adenocarcinoma (PDAC) by inhibiting the glutamine-utilizing enzyme GFAT1 with the small-molecule glutamine analog DON effectively reduces tumor self-renewal and metastatic potential. This treatment also remodels the TME by decreasing hyaluronan and collagen levels in the ECM, leading to elevated infiltration of CD8+ T cells. As a result, DON sensitizes PDAC tumors to anti-PD-1 therapy, promoting tumor regression and extending survival.^142,143^

The ECM, predominantly composed of proteins like collagen and glycosaminoglycans such as hyaluronic acid, plays a key role in modulating immune cell function within the TME. The extensive collagen network functions as a physical barrier to T cell infiltration, limiting their mobility and lowering their cytotoxic potency against tumor cells.^144,145^ Additionally, CAFs, vital to the TME, secrete cytokines and growth factors, and VEGF, which inhibit immune cell activation and function. They also promote the recruitment and development of immunosuppressive cells, sustaining the TME’s immunosuppressive environment and facilitating tumor progression.^144^ For example, in pancreatic cancer, CAFs promote immunosuppression by secreting cytokines and chemokines that recruit and differentiate immunosuppressive cells. CAFs express molecules, including IL-6 and CXCL12, which attract Tregs and suppress cytotoxic CD8+ T cells, reducing the anti-tumor immune response.^146,147^ Furthermore, CAFs contribute to the polarization of macrophages towards the immunosuppressive M2 phenotype, increasing the tumor’s ability to elude the immune system.^147,148^ This creates a feedback loop in which CAFs continually support tumor growth and metastasis while suppressing immune activity. Targeting CAF-mediated immunosuppressive pathways has shown promise in restoring CD8+ T cell function and improving the immune response against tumors in preclinical models, offering a potential strategy for enhancing immunotherapy efficacy in pancreatic cancer.^149,150^

Furthermore, TME significantly influences the expression of immune checkpoint molecules. Factors within the TME can lead to the overexpression of these checkpoints on tumor and immune cells, allowing tumors to modulate immune responses.^151^ Inflammatory cytokines, particularly interferon-γ, have been shown to elevate PD-L1 expression, fostering an immunosuppressive microenvironment that restricts host immune responses.^57,151^ For example, a recent article showed that oxymatrine, a compound with potent anti-cancer activity, enhances the tumor immune response and ferroptosis in liver cancer. The study demonstrated that oxymatrine downregulates PD-L1 expression and key ferroptosis-related proteins, such as xCT and GPX4, in liver cancer cells. In in vivo models, oxymatrine, either alone or in combination with anti-PD-L1 therapy, inhibited tumor growth more significantly than anti-PD-L1 alone. The combination treatment reduced interferon-γ expression and increased infiltration of tumor-infiltrating immune lymphocytes, including CD4+ T and CD8+ T cells, into the TME. Additionally, oxymatrine reversed the IFN-γ-induced upregulation of PD-L1 expression. It also promoted ferroptosis by elevating intracellular levels of Fe2+, reactive oxygen species, and malondialdehyde.^152^ Chronic antigen stimulation leads to elevated PD-1 expression in T cells. This results in T cell exhaustion and impaired function, which is often correlated with poor therapeutic outcomes. Additionally, genomic alterations and signaling pathways intrinsic to tumors further regulate PD-L1 expression, promoting immune evasion and cancer cell survival.^57^

Together, these factors underscore the TME’s critical role in immune checkpoint modulation, emphasizing the need for therapeutic strategies targeting the TME to enhance the efficacy of immunotherapies. Therefore, understanding the mechanisms by which TME modulates immune evasion has significant therapeutic implications. Strategies aimed at reprogramming the TME to enhance anti-tumor immunity are being actively explored. These include the use of drugs that target the recruitment and function of immunosuppressive cells, inhibitors of ECM remodeling, and agents that modify the metabolic landscape of the TME. Additionally, combining immune checkpoint inhibitors with therapies targeting TME components shows promise for overcoming resistance and enhancing immunotherapy efficacy.

### Antigen presentation and recognition

Antigen presentation and recognition allow the immune system to detect and eliminate abnormal cells, like cancer cells. Effective presentation of tumor-associated antigens (TAAs) and T cell recognition are key for a strong anti-tumor response. However, cancer cells disrupt these processes to evade detection, promoting immune evasion and tumor growth.^153,154^

Tumors evade immune detection by downregulating MHC class I molecules, which present TAAs to cytotoxic CD8+ T cells, thus inhibiting their activation and ability to destroy tumor cells. But, tumor cells escape immune surveillance by reducing MHC class I production via mechanisms like genetic mutations or deletions in MHC class I processing components, such as beta-2 microglobulin, TAP1, and TAP2,^11^ or the downregulation of transcription factors like NLRC5 that are required for MHC class I expression.^155^ This results in diminished MHC class I expression, hindering CD8 T cell recognition and allowing tumors to evade immune destruction (Fig. 1). Moreover, the role of YTHDF1 in regulating anti-tumor immunity has recently gained significant attention. Studies have demonstrated that YTHDF1 deficiency in dendritic cells (DCs) enhances the cross-priming of CD8+ T cells and improves the cross-presentation of tumor antigens in vivo.^156^ Recent research has illuminated that one mechanism driving the immune escape is the tumor-intrinsic factor YTHDF1, an m6A reader that regulates the translation of key lysosomal genes. YTHDF1 deficiency has been shown to restore the proteolysis of MHC-I molecules and their associated antigens, thereby enhancing tumor immune surveillance. This mechanism not only contributes to immune evasion but also facilitates resistance to immune ICIs, as YTHDF1 deficiency helps to convert “cold” tumors into “hot” tumors to improve their responsiveness to immunotherapy.^157^ MHC class I and MHC class II expression play pivotal roles in mediating resistance to immunotherapy, albeit through distinct mechanisms. In tumors with defective IFN-γ signaling, reduced MHC-I expression hinders T cell recognition, leading to immune evasion. However, tumors that maintain high MHC-I levels may still respond to immunotherapy, suggesting that strategies targeting the NF-κB pathway to sustain MHC-I expression could overcome resistance.^11,158^ Conversely, MHC-II+ tumors, particularly those treated with PD-1 blockade, develop adaptive resistance through the recruitment of CD4+ T cells and upregulation of inhibitory receptors like LAG-3 and FCRL6. Targeting the MHC-II/FCRL6 axis, in combination with PD-1 blockade, could enhance immunotherapy efficacy in MHC-II+ tumors.^159^ Together, these findings highlight the importance of MHC molecules in modulating immune responses and suggest potential therapeutic strategies to overcome resistance to immune checkpoint inhibitors.

Tumor cells frequently acquire genetic mutations or endure epigenetic alterations that affect the function of the antigen processing machinery (APM), resulting in poor antigen presentation to T cells, a critical mechanism of immune evasion.^160^ The APM is responsible for generating peptide fragments from cellular proteins and loading them onto MHC molecules for presentation to T cells. Mutations in APM genes, such as those encoding proteasome subunits like LMP2, LMP7, and LMP10, can alter the repertoire of peptides generated and presented on MHC class I molecules.^161^ For example, genetic variations in the APM components, specifically the TAP2, LMP7, and ERAP1 genes, have been linked to a higher risk of cervical cancer. A specific combination of haplotypes covering these sites has been associated with a threefold increase in cervical cancer risk. This suggests that SNPs in these APM genes may contribute to the susceptibility and progression of the disease.^162^ Furthermore, epigenetic processes in cancer cells, such as DNA methylation and histone modifications, can control the expression of APM components. Defects in key APM elements like TAP, tapasin, and MHC class I are frequently observed across various human tumors, disrupting the tumor cells’ ability to present antigens and be recognized by tumor-specific cytotoxic T cells, thereby facilitating immune evasion.^161,163^ For example, hypermethylation of promoters or enhancers of genes such as TAP, tapasin, and MHC class I itself leads to their downregulation, impairing antigen presentation and facilitating tumor escape from cytotoxic T cell recognition. This mechanism has been documented in several cancer types, where DNA methylation recruits repressive factors that silence these genes, and treatment with DNA demethylating agents can restore their expression, highlighting the role of epigenetic silencing in immune evasion.^164^

Moreover, immune editing also plays a key role in immune evasion. Immune editing is a process where the immune system selects for tumor cell variants lacking immunogenic antigens, consisting of three phases: elimination, equilibrium, and escape.^165^ In the elimination phase, innate and adaptive immune systems collaborate to destroy early tumor cells, with dendritic cells activating T cells for targeted attacks.^166^ For instance, a study on MCA-induced sarcomas showed that tumors from immunodeficient Rag2-/- mice were more immunogenic when transplanted into wild-type mice, underscoring the immune system’s role in eliminating highly immunogenic tumor cells in normal hosts.^166^ However, some tumor cells may survive this phase and enter the equilibrium phase, where they remain dormant or proliferate slowly while under continuous immune surveillance. During the escape phase, tumor cells that have acquired genetic or epigenetic modifications that allow them to avoid immune identification take precedence. These modifications may include the deletion or mutation of TAAs,^167^ alterations in the expression of co-stimulatory molecules,^1^ or changes in the TME that inhibit T cell activation.^1,167^ As a result, these immune-edited tumor cells can proliferate uncontested, leading to tumor progression and metastasis. For example, a study of the d42m1 sarcoma cell line revealed that T cell-mediated immunoselection resulted in the emergence of tumor variants devoid of strong rejection antigens. Specifically, escape variants lacked a point mutation in the Spectrin-β2 gene that produced a neoantigen in the initial tumor.^168^

Furthermore, antigen presentation and recognition are fundamental for enabling the immune system to identify and destroy abnormal cells, including cancerous ones. In cancer, the successful presentation of TAAs and their recognition by T cells are key to triggering a strong anti-tumor immune response. Cancer cells have evolved several mechanisms to interfere with these processes, leading to immune evasion and promoting tumor progression.^169,170^ However, tumors can evade immune detection by disrupting these co-stimulatory pathways. For example, tumor cells can express immune checkpoint molecules, sending inhibitory signals that counteract co-stimulatory signals and cause T cell anergy or apoptosis.^171,172^ Additionally, tumors can modulate the expression of co-stimulatory molecules on APCs by downregulating CD80 and CD86 or upregulating inhibitory receptors, thereby creating an immunosuppressive environment that impairs T cell recognition and activation.^169,173^ This ability of tumors to manipulate immune responses underscores their capacity to hinder effective anti-tumor immunity.

Recently, the role of T cell exhaustion has been widely discussed in the immune evasion of cancer. T cell exhaustion is marked by reduced proliferation, cytotoxic activity, and impaired effector cytokine production, weakening the immune response against tumors.^174^ This exhaustion involves the overexpression of inhibitory receptors, which impair T cell function by reducing activity and promoting immune tolerance toward tumor antigens.^174^ In various tumor models, co-expression of PD-1, TIM-3, and LAG-3 on CD8+ TILs leads to increased T cell exhaustion, with TIM-3 + PD-1+ cells showing the strongest exhaustion signals.^175^ Similarly, in melanoma, TIM-3 +PD-1 + CD8+ T cells are more dysfunctional than TIM-3-PD-1+ and TIM-3-PD-1- cells.^176^ Preclinical studies found that inhibiting both PD-1 and TIM-3 rejuvenated the anti-tumor activity of exhausted CD8+ T cells. Additionally, co-expression of PD-1 and LAG-3 indicates a higher level of exhaustion in CD8+ TILs, with dual blockade of these checkpoints leading to tumor regression.^177^ In ovarian cancer, LAG-3 + PD-1 + CD8+ T cells showed significantly reduced IFN-γ and TNF-α production compared to their single-positive counterparts.^178^ Moreover, antigen presentation and recognition are essential for the immune system to identify and eliminate abnormal cells. In cancer, the efficient presentation of TAAs and their recognition by T cells are crucial for triggering a robust anti-tumor immune response. However, cancer cells have evolved various strategies to interfere with these processes, leading to immune evasion and promoting tumor progression.^174^

Understanding the mechanisms by which tumors evade antigen presentation and recognition has significant implications for cancer therapy. Strategies to enhance antigen presentation, such as using interferons to upregulate MHC class I expression or employing therapies that target the antigen processing machinery, are being explored to improve immune recognition of tumors. Additionally, immune checkpoint inhibitors that reactivate exhausted T cells have revolutionized cancer treatment, offering new hope for patients with malignancies that have evaded immune detection.

## Genetic and epigenetic influences on immune evasion

### Role of oncogenes and tumor suppressor genes

**Fig. 2:**
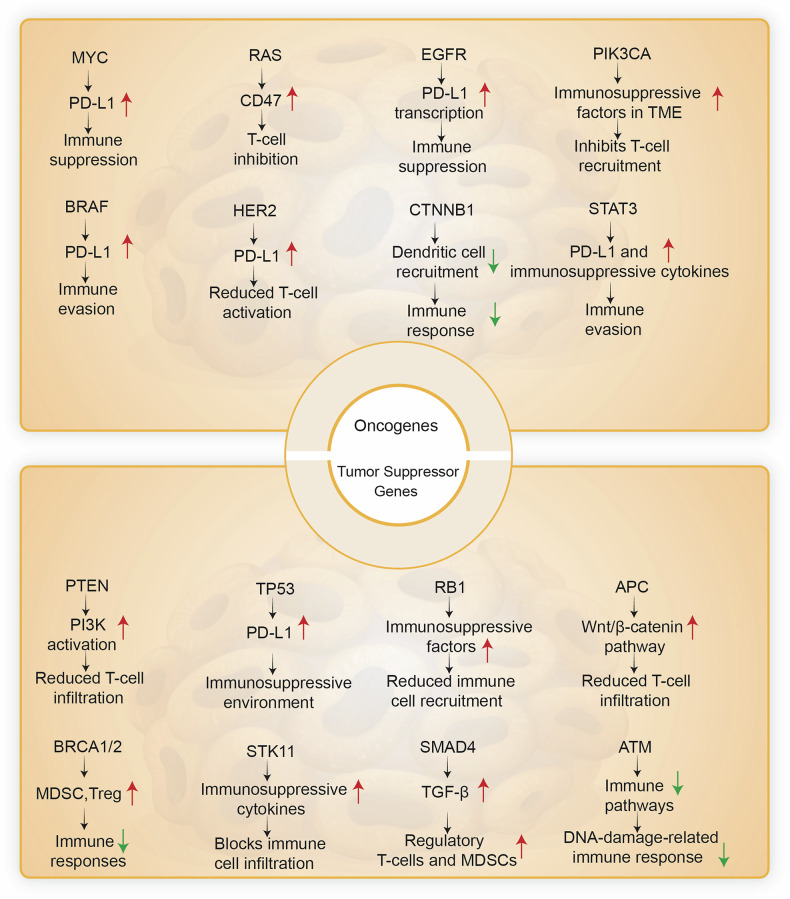
Immune evasion mechanisms driven by oncogenes and tumor suppressor gene loss in cancer. Oncogenes and tumor suppressor genes contribute to immune evasion through diverse mechanisms, aiding tumor cells in avoiding immune detection and elimination. For example, the Myc increases the expression of PD-L1 and leads to immune suppression. Similarly, the RAS family can increase CD47, which leads to T cell inhibition. Mutations in the EGFR, BRAF, and HER2 upregulate PD-L1 expression, leading to immune evasion. STAT3 promotes immune suppression by inducing PD-L1 and immunosuppressive cytokines. In contrast, tumor suppressor gene loss, such as TP53 and PTEN, increases PD-L1 and PI3K pathway activation to enhance immune evasion. RB1 loss increases immunosuppressive factors, preventing immune cell infiltration. APC loss activates the Wnt/β-catenin pathway, reducing T cell infiltration. BRCA1/2 and SMAD4 loss promote the recruitment of regulatory T cells and MDSCs, strengthening the immunosuppressive microenvironment. ATM loss suppresses immune signaling related to the DNA damage response, further reducing immune activation. Each of these genetic changes fosters immune evasion, creating a hostile microenvironment for anti-tumor immune responses

Cancer cells’ ability to evade the immune system is crucial for tumor development and metastasis. Genetic and epigenetic alterations in key regulatory genes often facilitate this evasion. Among these, alterations in oncogenes and tumor suppressor genes play a central role, enabling tumors to manipulate immune responses and escape detection by the body’s natural defenses.^179^ Understanding the mechanisms by which these genetic changes influence immune evasion is critical for developing effective cancer therapies (Fig. 2).

Oncogenes are mutated or overexpressed forms of normal proto-oncogenes that induce cancer cell proliferation and survival. However, their role in immune evasion is equally significant. One of the primary ways oncogenes contribute to immune evasion is by modulating antigen presentation. The findings from recent studies highlight the dual role of oncogenes in cancer progression, acting not only as cell-intrinsic drivers of tumorigenesis but also as potential modulators of the immune response to cancer. For example, TRAP1 (Tumor Necrosis Factor Receptor-Associated Protein 1) supports tumor growth in various cancers, where it is linked to poor survival outcomes.^180^ However, downregulation of TRAP1 in human ovarian cancer promotes invasion and epithelial–mesenchymal transition (EMT), processes that are closely associated with cancer aggressiveness and metastasis. This indicates that TRAP1 also acts as a suppressor of cancer progression, and its loss may enhance tumor invasiveness and the ability of cancer cells to spread.^181^ Similarly, histone deacetylase 1 (HDAC1) has a complex role. It functions as an oncosuppressor during the early stages of tumorigenesis but can promote malignancy during the maintenance phase of established tumors.^182^ KRAS, frequently mutated in various cancers, plays a critical role in initiating tumorigenesis. It alters signaling pathways and metabolism to promote tumor growth.^183,184^ However, research indicates that the loss of KRAS expression in advanced stages can lead to tumor regression. This suggests that while KRAS drives tumor development, it may not be necessary for maintaining established tumors.^185^ Specifically, the catalytically active mutation in the PI3K pathway, PIK3CA H1047R, not only provides tumors with a growth advantage but also aids in immune evasion by altering the TME. This mutation reduces the infiltration of cytotoxic CD8+ T cells and increases the number of inhibitory myeloid cells, generating an immunosuppressive environment that reduces the efficiency of immunotherapy.^186^ Additionally, oncogenic KRAS has been linked to the upregulation of CD47, a molecule that helps tumor cells avoid phagocytosis by macrophages, further illustrating the multifaceted ways oncogenes can facilitate immune evasion.^187^ Moreover, mutations in key oncogenes such as KRAS and STK11 play a significant role in mediating resistance to ICIs in NSCLC. KRAS mutations, particularly the KRAS-G12D substitution, have been shown to impair the tumor immune microenvironment (TIME) by reducing PD-L1 expression and chemokine secretion, which limits CD8+ T cell recruitment and promotes immunosuppression. This results in inferior efficacy of PD-1/PD-L1 blockades. Interestingly, combining chemotherapy with ICIs, such as paclitaxel, to enhance CXCL10/CXCL11 levels, has shown promise in overcoming this resistance.^188^ Similarly, mutations in the STK11 gene are associated with a suppressive immune phenotype, characterized by increased myeloid cell infiltration and elevated STAT3 signaling, which contributes to resistance to PD-L1/CTLA-4 blockade. Preclinical data suggest that targeting STAT3 in STK11-mutant tumors can reverse resistance and restore immune sensitivity.^189^ These findings highlight how mutations can alter the immune landscape and suggest that combination therapies targeting the underlying molecular mechanisms, such as chemotherapy or STAT3 modulation, may overcome resistance and enhance the efficacy of immunotherapy in patients with KRAS or STK11 mutations.

In addition to altering antigen presentation, oncogenes can increase the expression of immune checkpoint molecules, which suppress immune cell function. A well-known example is the PD-L1, whose expression is often increased by oncogenic signaling pathways such as PI3K/AKT/mTOR.^190^ PD-L1 binds to T cells’ PD-1 receptor, giving an inhibitory signal that decreases T cell activity and helps tumor cells escape immune attack.^57,191^ This upregulation of PD-L1 represents a key mechanism by which oncogenes create an immunosuppressive environment, protecting tumor cells from immune-mediated destruction.

Furthermore, oncogenes may induce the release of immunosuppressive cytokines and chemokines, further dampening immune responses. The MYC oncogene produces immunosuppressive substances. Such as the TGF-β inhibits CTL and NK cell activation and proliferation, whereas IL-10 lowers APC activity.^192,193^ By promoting the release of these immunosuppressive factors, oncogenes help create a TME that favors immune evasion.

Tumor suppressor genes, on the other hand, function to prevent uncontrolled cell growth and maintain cellular integrity. Their loss or inactivation is linked with cancer and contributes significantly to immune evasion. Loss of tumor suppressor genes enhances immune evasion by downregulating the antigen presentation machinery. For example, the p53 tumor suppressor gene regulates MHC expression. Loss of p53 function, which occurs in more than half of all cancers, leads to reduced MHC expression, thereby diminishing the tumor’s visibility to CTLs and facilitating immune escape.^194^ A recent study revealed that the development of monoclonal antibodies (mAbs) targeting the p53 hotspot mutation E285K, delivered via lipid nanoparticles (LNPs), shows promise as a precision medicine approach for treating cancers with mutant p53, leveraging both TRIM21 and PIGR pathways for enhanced anti-tumor immunity.^195^

The impact of tumor suppressor gene loss on immune evasion is also evident in the regulation of immune checkpoint molecules. For example, the absence of the PTEN tumor suppressor gene has been linked to increased expression of PD-L1. PTEN typically suppresses the PI3K/AKT/mTOR signaling pathway, and its absence not only promotes tumor development but also improves immunological checkpoint signaling, thus producing an immunosuppressive environment that permits malignancies to escape immune surveillance.^196,197^

Epigenetic alterations in tumor suppressor genes further compound immune evasion. DNA methylation and histone changes can silence tumor suppressor genes, resulting in decreased production of immune-stimulatory molecules and increased secretion of immunosuppressive factors. For example, the epigenetic repression of BRCA1 and BRCA2, genes implicated in DNA repair, has been associated with altered immune signaling in the TME.^198^ Additionally, the epigenetic alteration of CDKN2A can alter the TME, promoting immune evasion by recruiting regulatory Tregs and MDSCs, both of which suppress anti-tumor immunity.^199^ Moreover, the pivotal role of epigenetic regulation in T cell exhaustion within the TME has been underscored by recent findings that link the loss of histone methyltransferase EZH2 to mitochondrial dysfunction and impaired T cell function. A recent study has elucidated a novel Cdkn2a.Arf-mediated, p53-independent pathway through which EZH2 inhibition contributes to metabolic exhaustion in TILs. This insight opens up promising avenues for enhancing the efficacy of cellular therapies by engineering T cells with gain-of-function EZH2 mutants, potentially enabling these cells to better navigate the metabolic challenges posed by the tumor milieu and resist pharmacological stressors.^200^

Overall, the interplay between oncogenes and tumor suppressor genes is pivotal in the process of immune evasion in cancer. Oncogenes actively promote immune escape by modulating antigen presentation, upregulating immune checkpoints, and secreting immunosuppressive cytokines. Conversely, the loss of tumor suppressor gene function exacerbates immune evasion by diminishing antigen presentation and fostering an immunosuppressive microenvironment.

### Epigenetic modifications

Epigenetic modifications enable cancer cells to evade immune surveillance by regulating gene expression. Reversible changes, such as DNA methylation,^201,202^ histone modifications,^203^ and non-coding RNA regulation,^204^ can modify the expression of genes involved in immune recognition and response, allowing cancer cells to escape detection and destruction.^205^

**Fig. 3:**
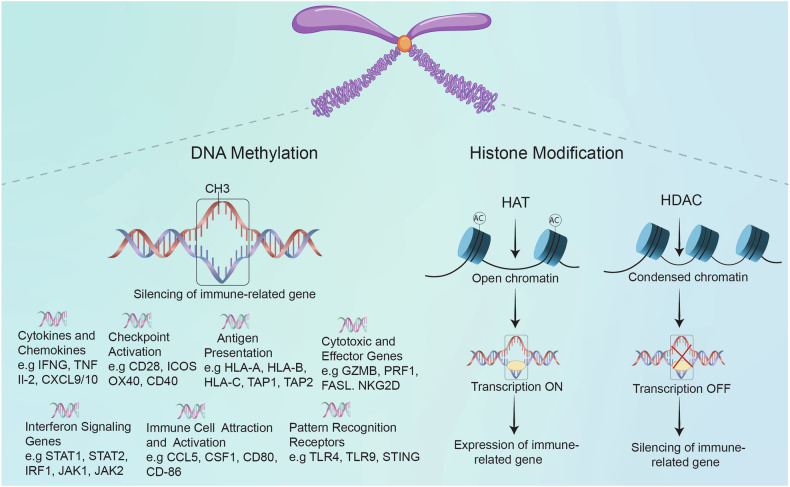
DNA methylation, histone modification, and immune evasion in cancer. This figure illustrates the interplay between key immune-related genes and DNA methylation and histone modification in orchestrating the immune response against tumors in cancer immunology. In the DNA methylation section, hypermethylation of the promoter regions of immune-related genes is shown to repress their expression, reducing immune cell infiltration into the tumor. This is depicted by a DNA strand marked by methyl groups, which connect to reduced gene expression and limited immune presence near the tumor. In the histone modification section, HDACs and histone acetyltransferases (HATs) modify chromatin structure, either condensing it (heterochromatin) or relaxing it (euchromatin), respectively. HDAC activity leads to closed chromatin around immune-regulatory genes, silencing their expression, while HAT activity may open chromatin but does not effectively activate immune-activating genes in tumors. These repressive changes result in immunosuppressive TME, with immune cells depicted as partially blocked or inactive. This illustrates how epigenetic modifications support immune evasion by repressing immune-modulatory molecules and limiting immune cell access to the tumor

One of the most extensively researched epigenetic alterations is DNA methylation (Fig. 3). A typical method of gene inactivation in cancer is hypermethylation of tumor suppressor gene promoter regions. This epigenetic silencing can extend to genes important in the immunological response, such as those that encode components of the antigen presentation machinery. For instance, in a novel approach to pancreatic cancer therapy, a study demonstrates the potential of epigenetically modified vaccines to enhance anti-tumor immune responses. By demethylating the CIITA gene in the pancreatic carcinoma cell line PANC-1, which successfully increased the expression of major MHC-II, which is crucial for antigen presentation. When administered to C57BL/6J mice, this epigenetically modified vaccine increased the proliferation of antigen-specific T cells, enhanced the cytotoxic activity of CTLs, and shifted the cytokine profile towards a Th1-type response, with increased IFN-γ and IL-2 secretion and reduced IL-4 and TGF-β levels.^206,207^ Furthermore, DNA methylation plays a critical role in modulating tumor resistance to immunotherapy by influencing immune evasion mechanisms. Loss of global DNA methylation, particularly in late-replicating partial methylation domains, correlates with immune escape signatures in tumors, independent of mutation burden and aneuploidy. These methylation alterations lead to transcriptional repression of immunomodulatory pathway genes, increasing resistance to immunotherapy. Methylation loss was found to have higher predictive power for immunotherapy resistance than mutation burden, highlighting its significance as an epigenetic modifier in immune evasion.^208^ Therefore, targeting DNA methylation could provide novel strategies to overcome resistance and improve the clinical benefit of immunotherapy.

In addition to DNA methylation, histone modifications are vital for regulating gene expression in cancer. Histones, which encase DNA, undergo post-translational modifications and ubiquitination that can either enhance or inhibit gene transcription.^209^ In immune evasion, histone modifications can change the expression of immune-related genes (Fig. 3). For example, histone deacetylation, mediated by HDACs, is associated with chromatin condensation and transcriptional repression. HDAC overexpression in cancer has been associated with the silencing of genes involved in antigen presentation and immune activation, further promoting an immune-suppressive TME.^210,211^ In contrast, inhibiting HDACs has been shown to restore the expression of genes that enhance tumor cell visibility to the immune system, thereby improving the effectiveness of immunotherapies. Similarly, histone modification also plays a key role in promoting resistance to immunotherapy. For example, the histone methyltransferase EZH2 has been shown to contribute to resistance by silencing tumor immunogenicity and antigen presentation, particularly during T cell-targeting therapies. Inactivation of EZH2 has been demonstrated to reverse resistance and enhance the efficacy of immunotherapies.^212^ Moreover, a recent study utilizing CRISPR-Cas9 screens to target chromatin regulators in mouse tumor models has identified the H3K9 methyltransferase SETDB1 as a key mediator of immune evasion. SETDB1, in conjunction with other members of the HUSH and KAP1 complexes, is known to repress transposable elements and immune-related genes. This repression contributes to immune exclusion and resistance to immune checkpoint blockade. Amplification of SETDB1 in human tumors has been associated with immune evasion, while the loss of SETDB1 can trigger anti-tumor immune responses.^213^

### Non-coding RNAs and immune evasion

Non-coding RNAs (ncRNAs), particularly miRNAs, long non-coding RNAs (lncRNAs), and circular RNAs (circRNAs) have emerged as pivotal players in cancer’s ability to evade the immune system. Though these RNA molecules do not code for proteins, they regulate gene expression in ways that support tumor growth and immune escape or inhibit the immune escape. Studies have reported several miRNAs that play a role in immune evasion in cancer (Table 1). For example, specific miRNAs such as miR-200 and miR-21 can upregulate checkpoint proteins like PD-1 and PD-L1, molecules that interact with T cells to dampen their anti-tumor activity. By promoting immune checkpoint expression, these miRNAs contribute to T cell exhaustion, diminishing the immune system’s ability to detect and destroy cancer cells.^214,215^ Furthermore, miRNAs play a significant role in mediating resistance to immune therapies, particularly in cancers treated with trastuzumab-based chemotherapy. For example, miR-1246, miR-155, and miR-146a-5p have been identified as key regulators of trastuzumab resistance in HER2-positive breast cancer. Elevated levels of miR-1246 and miR-155 in circulating exosomes are associated with poor event-free survival and progression-free survival in trastuzumab-treated patients. These miRNAs have the potential to serve as predictive biomarkers of resistance.^216^ Similarly, miR-146a-5p is upregulated in trastuzumab-resistant cancer cells and exosomes. This miRNA promotes resistance by increasing EMT, migration, angiogenesis, and cell cycle progression. Exosomal transfer of miR-146a-5p further reduces trastuzumab sensitivity in sensitive cells.^217^ Therefore, targeting miRNAs or their exosomal transfer could be a promising strategy to overcome resistance and improve the efficacy of immune-based therapies in HER2-positive breast cancer.

**Table 1:** Role of miRNAs in immune evasion in cancer

miRNA	Role	Effect on immune evasion	Reference
miR-21	Oncogenic	Promotes immune suppression by upregulating PD-L1 expression and inhibiting apoptosis in T cells.	^214,543,544^
miR-155	Oncogenic	Modulates macrophage polarization to an M2 phenotype, enhancing immune evasion in the TME.	^545–547^
miR-34a	Tumor suppressor	Increasing the expression of miR-34a downregulates PD-L1 and MET, restoring T cell function and sensitivity to immune checkpoint inhibitors.	^548^
miR-125b-5p	Tumor suppressor	miR-125b-5p was found to reduce the function of Treg cells by downregulating TNFR2. It also plays a role in invasion.	^549^
miR-200c-3p	Tumor suppressor	Targets PD-L1, reducing its expression and potentially sensitizing cancer cells to T cell-mediated killing.	^550^
miR-29	Tumor suppressor	miR-29b counteracts the tumor-promoting, pro-inflammatory activity of dendritic cells influenced by multiple myeloma. It promotes NK cell activation and promotes CD8+ T cell infiltration into tumors.	^551,552^
miR-210	Oncogenic	Increased expression of miR-210 in hypoxic tumor cells reduced their susceptibility to lysis by cytotoxic T lymphocytes (CTLs)	^553^
miR-223	Oncogenic	miR-223 plays an important role in regulating innate immune responses, particularly in neutrophils and macrophages.	^554^
miR-138	Tumor suppressor	miR-138 enhances anti-tumor immunity by downregulating immune checkpoints (PD-1, CTLA-4 on T cells, and PD-L1 on tumor cells), boosting T cell activity, and promoting dendritic cell function.	^555,556^
miR-125a	Dual role	miR-125a promotes immunosuppressive myeloid cells, reduces antigen presentation, and impairs NK cell function, enabling tumors to evade immune detection.	^557,558^
miR-27a	Oncogenic	miR-27a promotes immune evasion in breast cancer by upregulating PD-L1 in macrophages and reducing MHC class I on cancer cells, hindering T cell recognition and activity.	^558,559^
miR-424	Tumor suppressor	miR-424 targets immune checkpoints like CTLA-4 and PD-L1/PD-1, helping cancer cells to evade immune detection. It modulates the TME by altering cytokine secretion and immune recognition, supporting immune escape.	^558,560^
miR-19a	Oncogenic	miR-19a promotes immune evasion in cancer by downregulating interferon-regulated genes, reducing MHC class I expression, and modulating cytokines. These actions impair T cell recognition, weaken anti-tumor immune responses, and can also inhibit NK cell function.	^561^
miR-214	Oncogenic	miR-214 promotes cancer immune evasion by inducing Tregs expansion, enhancing IL-10 secretion, and fostering immune suppression. Tumor-derived miR-214 boosts Tregs by downregulating PTEN in CD4 + T cells and promotes metastasis via the tumor stroma.	^562,563^
miR-9	Tumor suppressor	miR-9 regulates the expression of interferon-responsive genes and MHC class I molecules in human nasopharyngeal carcinoma cells.	^564^
miR-20a	Oncogenic	miR-20a has been shown to promote immune evasion by targeting MICA/B, which are ligands for the activating NK cell receptor NKG2D. By downregulating MICA/B, miR-20a helps cancer cells evade NK cell-mediated killing,	^558,565,566^
miR-142-5p	Tumor suppressor	miR-142-5p has been found to modulate PD-L1 expression and influence T cell responses in various cancers.	^567^
miR-197	Tumor suppressor	miR-197 miRNA has been implicated in regulating CKS1B and STAT3 signaling, affecting PD-L1 expression and immune evasion in NSCLC.	^568,569^
miR-375	Oncogenic	miR-375 facilitates immune evasion by enhancing macrophage recruitment, infiltration, and pro-tumor polarization.	^570^

Furthermore, most studies have reported that lncRNAs generally promote immune evasion rather than inhibit it (Table 2). For example, research has shown that Lnc-EGFR is crucial in Treg-mediated immunosuppression and the development of hepatocellular carcinoma (HCC). By stabilizing EGFR and stimulating its downstream signaling, lnc-EGFR promotes Treg development while suppressing CTL activity, ultimately leading to tumor growth.^218^ Furthermore, another study found that LncRNA NKILA regulates T cell sensitivity to activation-induced cell death (AICD) in the TME. NKILA inhibits NF-κB activity, preventing T cells from apoptosis and promoting their anti-tumor action.^219^ Additionally, lncRNAs like SNHG1 increase the presence of immunosuppressive cells, creating a more immunosuppressive environment that favors tumor survival.^220^ Moreover, lncRNAs play a significant role in mediating resistance to immunotherapy in breast cancer. For example, LINC00624 inhibits the anti-tumor effects of HER2-targeted therapies by suppressing type I interferon (IFN) pathway activation. It binds to and is edited by the RNA-editing enzyme ADAR1, which reduces the IFN response and promotes immune suppression. LINC00624 also stabilizes ADAR1, preventing its degradation, thereby impairing MHC class I antigen presentation and limiting CD8+ T cell infiltration. This leads to immune checkpoint blockade resistance and reduced efficacy of HER2-targeted therapies.^221^ Similarly, TINCR contributes to immune escape by upregulating PD-L1 expression, which dampens the immune response. TINCR stabilizes USP20 mRNA and inhibits PD-L1 ubiquitination through a competing endogenous RNA (ceRNA) mechanism. It also recruits DNMT1 to methylate miR-199a-5p, further promoting PD-L1 expression. Knockdown of TINCR improves the therapeutic response to PD-L1 inhibitors in vivo, highlighting its role in immunotherapy resistance.^222^ Therefore, targeting lncRNAs could restore immune responses and enhance the effectiveness of immunotherapies.

**Table 2:** Role of lncRNAs in immune evasion in cancer

lncRNA	Role	Effect on immune evasion	Reference
MALAT1	Oncogenic	MALAT1 aids cancer immune evasion by modulating immune cells, suppressing T cell responses, enhancing cytokine production, and promoting metastasis. It also facilitates immune evasion and metastatic reactivation of dormant cancer cells. Its inhibition reduces immune evasion and may enhance the effects of checkpoint inhibitors.	^571^
HOTAIR	Oncogenic	HOTAIR plays a key role in cancer immune evasion by activating the NF-κB pathway, upregulating PD-L1, and modulating inflammatory signaling, which fosters an immunosuppressive TME.	^572^
NEAT1	Oncogenic	NEAT1 enhances immune escape by upregulating PD-L1, promoting T cell apoptosis and exhaustion, and recruiting immunosuppressive cells. It fosters an immunosuppressive TME and may impact antigen presentation, aiding immune evasion.	^573,574^
LINC00473	Oncogenic	LINC00473 promotes immune evasion in cancer by regulating PD-L1, acting as a competing endogenous RNA to modulate PD-L1 levels and help cancer cells evade immune detection. It also reshapes the TME and influences T cell recognition and cytotoxicity, reducing immune system effectiveness against tumor cells.	^575^
SNHG16	Oncogenic	SNHG16 supports immune evasion in breast cancer by promoting immunosuppressive CD73 + γδT1 regulatory T cells via the TGF-β1/SMAD5 pathway. It enhances CD73 expression, increasing adenosine levels in the TME, which suppresses anti-tumor immunity and facilitates tumor growth.	^576^
NKILA	Oncogenic	NKILA promotes tumor immune evasion by inducing apoptosis in tumor-specific CTLs, reducing their infiltration and activation. It disrupts the cancer immunity cycle by impairing T cell priming, trafficking, and tumor cell killing.	^577,578^
FENDRR	Tumor suppressor	FENDRR acts as a tumor suppressor by inhibiting immune evasion, enhancing anti-tumor immunity, and promoting T cell infiltration. It suppresses Treg-mediated immune escape and activates immune-related pathways in tumors.	^579,580^
XIST	Oncogenic	XIST promotes cancer immune evasion by regulating immune checkpoints, influencing immune cell infiltration, and modulating cytokines. It interacts with miRNAs, maintains cancer stem cells, and contributes to an immunosuppressive microenvironment.	^581^
LINC-PINT	Tumor suppressor	LINC-PINT acts as a tumor suppressor by inhibiting cell invasion and proliferation in cancers like breast and lung. However, in colon cancer, higher expression is linked to immune evasion, though it may improve response to ICIs.	^582,583^
GAS5	Tumor suppressor	GAS5 enhances anti-tumor immunity by promoting immune cell infiltration, particularly macrophages and T cells, in the TME. It upregulates pro-inflammatory cytokines and type I interferon signaling, which may reduce immune evasion.	^584^
CCAT1	Tumor suppressor	CCAT1 acts as a tumor suppressor by inhibiting M2 macrophage polarization and tumor cell migration. Downregulation of CCAT1 promotes these processes via upregulation of miR-148a, which targets PKCζ.	^585^
LINK-A	Oncogenic	LINK-A promotes immune evasion in TNBC by downregulating antigen processing and presentation components	^586,587^
GHSROS	Oncogenic	GHSROS is involved in breast cancer immune evasion, promoting cell migration and progression	^586,588^
LNMAT1	Oncogenic	LNMAT1 is upregulated in bladder cancer with lymph node metastasis, enhances immunosuppressive lymphocyte recruitment.	^589,590^

circRNAs are a unique class of ncRNAs with a covalently closed-loop structure, which makes them highly stable and resistant to exonuclease degradation. This stability allows circRNAs to play important roles in gene regulation, cell signaling, and maintaining cellular homeostasis. Recently, they have gained attention for their involvement in cancer, particularly in immune evasion mechanisms. CircRNAs are generated through back-splicing, where a downstream 5′ splice site is joined to an upstream 3′ splice site, forming a closed loop. Their stability, especially compared to linear RNAs, makes them significant regulators in cancer, contributing to immune evasion and promoting tumor progression.^223,224^

Cancer cells often use a variety of strategies to evade immune surveillance, enabling them to grow, survive, and metastasize. CircRNAs have been shown to contribute to these immune evasion strategies (Table 3). They regulate immune checkpoint molecules, influence immune cell infiltration, alter the behavior of TAMs, and modulate other immune response pathways.^225^ These actions allow tumors to avoid being recognized and attacked by the immune system. One of the key mechanisms of immune evasion in cancer is the activation of immune checkpoints. These checkpoints prevent immune cells from attacking tumor cells. CircRNAs have been found to regulate the expression of immune checkpoint molecules. For example, recent research identified circRNA-002178 as being notably elevated in both cancer tissues and LUAD cell lines. This circRNA enhances PD-L1 expression by sponging miR-34, which leads to T cell exhaustion and helps the tumor evade immune surveillance. Moreover, circRNA-002178 was detected in exosomes from the plasma of LUAD patients, suggesting its potential as an early diagnostic biomarker for LUAD. Additionally, circRNA-002178 could be transferred into CD8+ T cells through exosomes, where it induces PD-1 expression, further contributing to immune suppression.^226^

**Table 3:** Role of circRNAs in immune evasion in cancer

circRNA	Role	Effect on immune evasion	Reference
circE7	Oncogenic	CircE7 promotes immune evasion in HNSCC by suppressing CD8+ T cell infiltration and function. It achieves this by downregulating the transcription of LGALS9, which encodes galectin-9. This reduction in galectin-9 expression impairs T cell activity, as galectin-9 interacts with immune checkpoints TIM-3 and PD-1, leading to T cell apoptosis and inhibition of cytotoxic cytokine secretion. The mechanism involves circE7 binding to acetyl-CoA carboxylase 1 (ACC1), promoting its dephosphorylation and activating ACC1, which reduces H3K27 acetylation at the LGALS9 promoter.	^591^
has_circ_0069313	Oncogenic	Has_circ_0069313 promotes immune evasion in OSCC by inhibiting miR-325-3p-induced degradation of Foxp3, a key regulator of Treg function. The upregulation of has_circ_0069313 in OSCC tissues correlates with poor prognosis and enhances Treg function through the maintenance of Foxp3 levels. Additionally, exosomal transfer of has_circ_0069313 to Treg cells further promotes immune suppression, aiding in the tumor’s immune escape.	^592^
CircKRT1	Oncogenic	CircKRT1 promotes immune evasion in OSCC by sponging miR-495-3p, which results in the upregulation of PD-L1. Increased PD-L1 expression suppresses CD8+ T cell cytotoxicity, enhances T cell apoptosis, and promotes immune suppression. Knockdown of CircKRT1 inhibits OSCC progression, reduces cell migration and invasion, and enhances CD8+ T cell cytotoxicity, suggesting that CircKRT1 modulates immune evasion through the miR-495-3p/PD-L1 axis. Additionally, CircKRT1 regulates cancer progression in vivo by modulating the miR-495-3p/PD-L1 pathway.	^593^
circIGF2BP3	Oncogenic	CircIGF2BP3 promotes immune evasion in NSCLC by sponging miR-328-3p and miR-3173-5p, leading to the upregulation of plakophilin 3 (PKP3). PKP3 stabilizes OTUB1 mRNA, which inhibits the ubiquitination and subsequent proteasomal degradation of PD-L1, thereby increasing PD-L1 expression. Elevated PD-L1 suppresses CD8+ T cell-mediated killing and impairs anti-tumor immunity. This mechanism was shown to reduce CD8+ T cell infiltration in NSCLC tumors. Inhibition of the circIGF2BP3/PKP3 axis enhances the efficacy of anti-PD-1 therapy in NSCLC mouse models.	^594^
hsa_circ_0000190	Oncogenic	Hsa_circ_0000190 promotes immune evasion in NSCLC by upregulating the expression of soluble PD-L1 (sPD-L1). This increase in sPD-L1 interferes with the efficacy of anti-PD-L1 antibodies and inhibits T cell activation, thereby contributing to immune resistance and poor outcomes in NSCLC patients. Elevated levels of hsa_circ_0000190 can be used as a biomarker for disease progression and therapeutic efficacy, suggesting its potential as a target for enhancing immunotherapy responses.	^595^
circ-CPA4	Oncogenic	Circ-CPA4 promotes immune evasion in NSCLC by serving as a sponge for let-7 miRNA, leading to the upregulation of PD-L1. This results in the inhibition of CD8+ T cell activation and facilitates immune suppression. Additionally, circ-CPA4 enhances the release of PD-L1-containing exosomes, which further contribute to immune evasion and resistance to cisplatin. Knockdown of circ-CPA4 reactivates CD8+ T cells, suggesting that circ-CPA4 plays a crucial role in modulating the tumor immune microenvironment and resistance to immunotherapy.	^596^
circ-KRT6C	Oncogenic	Circ-KRT6C promotes immune evasion in CRC by acting as a sponge for miR-485-3p, leading to the upregulation of PD-L1 expression. The increased PD-L1 expression suppresses immune responses, including inhibiting the activity of peripheral blood mononuclear cells and cytokine-induced killer cells. Knockdown of circ-KRT6C reduces cancer cell proliferation, migration, invasion, and immune escape, while promoting apoptosis. Additionally, circ-KRT6C inhibition suppresses tumor growth in vivo, suggesting it plays a significant role in immune evasion and cancer progression.	^597^
circFAT1	Oncogenic	CircFAT1 promotes immune evasion in squamous cell carcinoma (SCC) by regulating cancer stemness through STAT3 activation. By binding to STAT3, circFAT1 prevents its dephosphorylation by SHP1, leading to enhanced STAT3 activation. This activation inhibits STAT1-mediated transcription, impairing tumor cell-intrinsic immunity. Knockdown of circFAT1 reduces tumorsphere formation and inhibits tumor growth, while enhancing PD-1 blockade immunotherapy efficacy by promoting CD8+ T cell infiltration into the TME. These findings suggest that circFAT1 plays a key role in regulating the interaction between cancer stemness and immune evasion.	^598^
circ_0020710	Oncogenic	Elevated circ_0020710 promotes immune evasion in melanoma by upregulating CXCL12 via sponging miR-370-3p. High levels of CXCL12 lead to the exhaustion of cytotoxic lymphocytes, impairing effective anti-tumor immune responses. Additionally, a combination of AMD3100 (a CXCL12/CXCR4 axis inhibitor) and anti-PD-1 therapy significantly reduces tumor growth, suggesting that circ_0020710 contributes to immune evasion through the CXCL12/CXCR4 signaling axis.	^599^
circ-HSP90A	Oncogenic	Circ-HSP90A promotes immune evasion in NSCLC by sponging miR-424-5p to upregulate PD-L1 expression. This enhances immune suppression in the tumor microenvironment by impairing CD8+ T cell activity through the PD-1/PD-L1 axis. Additionally, circ-HSP90A stabilizes HSP90A and activates STAT3 signaling, further promoting tumor progression and resistance to immune responses.	^600^
circ_0101675	Oncogenic	Circ_0101675 promotes immune evasion in NSCLC by sponging miR-607 to upregulate PD-L1 expression. This enhances immune suppression by impairing T cell function through the PD-1/PD-L1 axis. Additionally, circ_0101675 promotes tumor progression, migration, invasion, and angiogenesis, facilitating tumor immune escape and resistance to immune surveillance.	^601^
hsa_circ_0136666	Oncogenic	Hsa_circ_0136666 promotes immune evasion in colorectal cancer by inhibiting miR-497 expression. This upregulates PD-L1, leading to the activation of Treg cells, which contribute to immune suppression and tumor immune escape. Hsa_circ_0136666 accelerates tumor growth and induces immune evasion through the miR-497/PD-L1/Treg axis.	^602^
circSOD2	Oncogenic	CircSOD2 promotes immune evasion in HCC by acting as a sponge for miR-497-5p. This leads to the upregulation of ANXA11, which enhances tumor progression. The circSOD2/miR-497-5p/ANXA11 axis also contributes to resistance against anti-PD-1 therapy by promoting immunosuppression and reducing CD8+ T cell infiltration.	^603^
circ-METTL15	Oncogenic	Circ-METTL15 promotes immune evasion in lung cancer by sponging miR-1299. This upregulates PD-L1, which contributes to immune escape and tumor progression. Downregulation of circ-METTL15 inhibits tumor growth in vivo via the miR-1299/PD-L1 axis.	^604^
circCRIM1	Oncogenic	CircCRIM1 inhibits immune evasion in NSCLC by binding to IGF2BP1, leading to the destabilization of HLA-F mRNA. This results in the restoration of immune responses, increasing the expression of Granzyme B, IFN-γ, and TNF-α in CD8+ T and NK cells, thus promoting anti-tumor immunity.	^605^
circBART2.2	Oncogenic	Promotes immune escape in nasopharyngeal carcinoma (NPC) by upregulating PD-L1 expression. This leads to inhibition of T cell function, facilitating tumor immune evasion.	^606^
circ_0001806	Oncogenic	Promotes immune escape in NSCLC by regulating PD-L1 expression and influencing cell proliferation, migration, and invasion.	^607^
EZH2-92aa	Oncogenic	Promotes immune evasion in glioblastoma stem cells (GSCs) by inhibiting NK cell response.	^608^
hsa_circ_0001479	Oncogenic	Promotes immune evasion by inhibiting CD8+ T cell infiltration and activating immune checkpoints.	^609^
hsa_circ_0050102 (circPGPEP1)	Oncogenic	Promotes immune escape by regulating tumor immunity. circPGPEP1 acts as a sponge for miR-515-5p, upregulating NFAT5 expression. This pathway regulates CRC cell proliferation, migration, EMT, and immune evasion.	^610^
circ_0058058	Oncogenic	Promotes immune escape by regulating PD-L1 expression in PC. circ_0058058 acts as a sponge for miR-557, upregulating PD-L1 expression, leading to enhanced tumor growth, invasion, angiogenesis, and immune evasion.	^611^
circEIF3C	Oncogenic	Induces immune evasion by upregulating B7-H4 in intrahepatic cholangiocarcinoma (ICC). circEIF3C sponges miR-34a-5p, leading to B7-H4 upregulation, which inhibits CD8+ T cell apoptosis and promotes tumor progression.	^612^
circ-0000512	Oncogenic	Promotes immune escape by inhibiting PD-L1 ubiquitination in TNBC. circ-0000512 sponges miR-622, leading to upregulation of CMTM6, which inhibits PD-L1 ubiquitination, reducing T cell killing activity and promoting tumor progression.	^613^
circRHBDD1	Oncogenic	Promotes immune escape by upregulating PD-L1 and inhibiting CD8+ T cell infiltration. circRHBDD1 binds IGF2BP2, disrupting its interaction with TRIM25, leading to IGF2BP2 stabilization, which increases PD-L1 mRNA stability through m6A modification.	^614^
circFOXK2	Oncogenic	circFOXK2 sponges miR-485-5p, leading to increased PD-L1 expression. This enhances tumor progression and impairs CD8+ T cell response to promote immune evasion.	^615^
circ-CTNNB1	Oncogenic	circ-CTNNB1 promotes immune escape by activating NF-κB signaling, increasing PD-L1 expression, and facilitating N2 polarization of neutrophils.	^616^
circ_0007422	Oncogenic	circ_0007422 is upregulated in CRC tissues and cells. Knockdown of circ_0007422 inhibits CRC cell proliferation, invasion, self-replication ability, and immune escape, while promoting cell apoptosis. circ_0007422 binds to miR-1256, which targets PD-L1, enhancing immune escape by upregulating PD-L1 and impairing CD8+ T cell function. In xenograft models, circ_0007422 knockdown reduces tumor growth and immune escape.	^617^
circATXN7	Oncogenic	circATXN7 is upregulated in KRASMUT tumors and plays a crucial role in immune evasion by regulating T cell sensitivity to activation-induced cell death (AICD). It binds to the NF-κB p65 subunit, preventing its nuclear localization, thus inactivating NF-κB. This leads to immune evasion by impairing T cell function. Upregulation of circATXN7 in tumor-specific CTLs is associated with poor clinical outcomes and immunotherapy resistance. Genetic ablation of circAtxn7 in CD8+ T cells enhances anti-PD-1 efficacy and tumor inhibition. Targeting circATXN7 improves anti-tumor activities of tumor-reactive CTLs.	^618^
hsa_circ_0004872	Tumor Suppressor	hsa_circ_0004872 acts as a tumor suppressor by inhibiting meningioma cell proliferation, metastasis, and immune escape. It enhances the cytotoxicity of CD8+ T cells by suppressing PD-L1 expression. hsa_circ_0004872 interacts with EIF4A3, leading to the degradation of PD-L1 mRNA, which reduces immune evasion. Inhibition of EIF4A3 improved immune escape, proliferation, and metastasis of meningioma cells.	^619^

Another way circRNAs contribute to immune evasion is by modulating TAMs. TAMs play a critical role in the TME and are often polarized into an immunosuppressive M2 phenotype, which promotes tumor growth and suppresses anti-tumor immune responses. CircRNAs can influence the polarization of macrophages by regulating the expression of inflammatory cytokines and chemokines.^227,228^ For example, circITGB6 enhances platinum resistance in ovarian cancer by inducing the polarization of TAMs toward the M2 phenotype. This immune-suppressive phenotype supports chemoresistance. The mechanism involves the stabilization of FGF9 mRNA through a complex with IGF2BP2, which promotes M2 polarization.^229^ Similarly, circPTPN22 is upregulated in pancreatic cancer and promotes tumor cell proliferation. It also alters the immune microenvironment by enhancing STAT3 acetylation. This is achieved by inhibiting the interaction between STAT3 and SIRT1.^230^

CircRNAs also play a significant role in regulating DCs, which are crucial for initiating adaptive immune responses by presenting tumor antigens to T cells. CircRNAs can influence the differentiation and function of DCs, thereby affecting their ability to prime T cells effectively. For example, circSnx5 controls the immunogenicity of DCs through the miR-544/SOCS1 axis and regulation of PU.1 activity, which enhances the DCs’ ability to prime T cells against tumors.^231^ circRNAs play a critical role in resistance to immunotherapy by promoting immune evasion in various cancers. For instance, circMET in HCC induces immune tolerance through the miR-30-5p/Snail/DPP4/CXCL10 axis and contributes to EMT. Targeting this axis with a DPP4 inhibitor like sitagliptin, combined with anti-PD-1 therapy, may enhance immune responses in HCC patients.^232^ Similarly, circNCOA3 in CRC sponges miR-203a-3p to increase CXCL1 expression, leading to immune escape and PD-1 blockade resistance. Knockdown of circNCOA3 restores CD8+ T cell function and improves sensitivity to PD-1 therapy.^233^ In HCC, circCCAR1, released in exosomes, promotes CD8+ T cell dysfunction by stabilizing PD-1, thereby contributing to anti-PD-1 resistance. Targeting the circCCAR1/miR-127-5p/WTAP feedback loop could reverse this effect and enhance treatment efficacy.^234^ Similarly, circHMGB2 in NSCLC reshapes the TME by sponging miR-181a-5p and inactivating the type 1 interferon response, leading to immune suppression and PD-1 resistance. Combining CARM1 inhibitors with anti-PD-1 therapy may overcome this resistance.^235^ CircFGFR1 in NSCLC interacts with miR-381-3p to upregulate CXCR4, promoting tumor progression and immune evasion. Targeting circFGFR1 could improve the response to PD-1 blockade.^236^ Additionally, exosomal circUSP7, secreted by NSCLC cells, induces CD8+ T cell dysfunction by sponging miR-934 and upregulating SHP-2 expression. This leads to decreased secretion of critical cytokines and cytotoxic proteins, impairing the anti-tumor immune response. Additionally, circUSP7 plays a role in resistance to anti-PD-1 therapy, highlighting circRNAs as key regulators of immune suppression in cancer and potential targets for improving immunotherapy efficacy.^237^ In gastric cancer, circDLG1 promotes immune evasion and PD-1 resistance by sponging miR-141-3p to enhance CXCL12 expression. Targeting circDLG1 could provide a strategy to overcome immune escape and improve the efficacy of anti-PD-1 therapy.^238^ Hence, disrupting circRNA-mediated immune suppression by targeting these feedback loops, inhibiting circRNA expression, or combining circRNA-targeting therapies with PD-1 blockade holds promise for improving immunotherapy outcomes.

Overall, ncRNAs have emerged as crucial regulators of immune evasion in cancer. They can influence various immune processes, such as immune checkpoint regulation, macrophage polarization, and T cell responses. By modulating key immune pathways, ncRNAs play a significant role in tumor immune escape. Several strategies are being investigated to target ncRNAs, including the use of small molecules or oligonucleotides to inhibit ncRNAs that promote immune evasion. Additionally, ncRNAs could be incorporated into therapeutic vaccines to enhance T cell recognition or block immune checkpoint pathways. However, challenges persist, including the functional diversity of ncRNAs across different cancers and difficulties in their stable delivery. Overcoming these obstacles and combining ncRNA-based therapies with existing immunotherapies could offer new approaches to improving cancer treatment outcomes and overcoming immune evasion.

## Impact of tumor heterogeneity on immune evasion

**Fig. 4:**
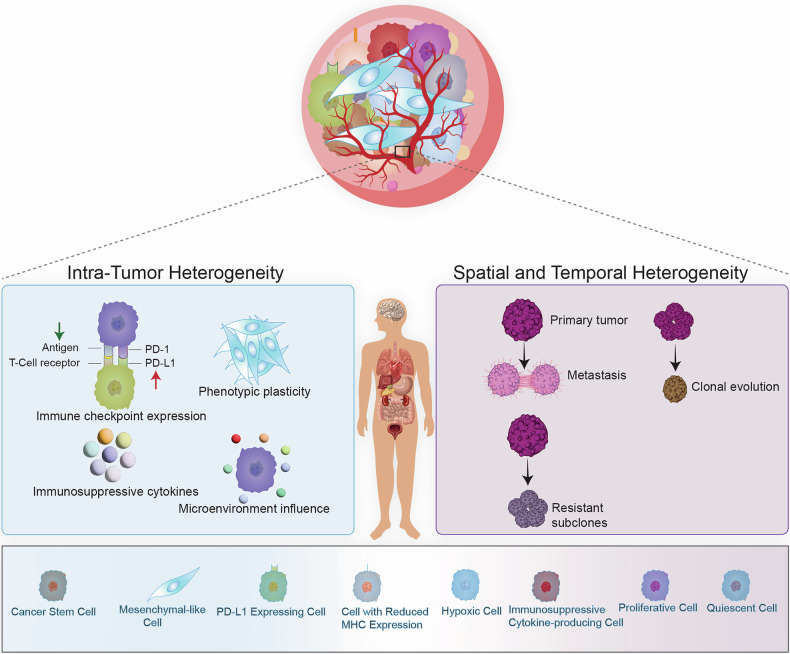
Tumor heterogeneity and immune evasion in cancer. This figure illustrates intra-tumor heterogeneity and the spatial-temporal dynamics of immune evasion in cancer. The diversity of cell populations in the tumor enables unique immune escape strategies that enhance resistance and contribute to cancer progression. Cancer stem cells exhibit self-renewal and releasing immune-suppressive factors to aid regrowth and metastasis. Mesenchymal-like cells undergo EMT, gaining motility and reducing antigen presentation for immune evasion. Cells expressing PD-L1 inhibit T cell activity through checkpoint interactions, while those with low MHC expression avoid cytotoxic detection by reducing antigen display. Hypoxic cells thrive in low-oxygen zones, fostering an immunosuppressive microenvironment and secret factors that create immune responses in cytokine-producing cells. Highly proliferative, mutationally diverse cells vary in antigen expression, complicating immune targeting, while dormant/quiescent cells evade detection through reduced activity but may later drive recurrence. Spatial heterogeneity within the TME and the variable distribution of immune cell populations across these regions are shown. A timeline tracking clonal evolution from primary tumor to metastasis illustrates temporal heterogeneity throughout tumor progression. There is also the emergence of resistance mechanisms, while depicted immune evasion strategies evolve over time, including loss of antigen presentation and increased regulatory T cells

In tumor immune evasion, both intra-tumor heterogeneity (ITH) and spatial and temporal heterogeneity play pivotal roles.^239^ However, they represent distinct aspects of tumor biology. ITH refers to the genetic, phenotypic, and functional diversity within a single tumor. Different subpopulations of tumor cells arise due to mutations and selective pressures. This leads to variations in immune evasion mechanisms, such as immune checkpoint expression or antigen loss. This diversity complicates the immune response. Some subclones may evade immune surveillance more effectively, contributing to therapeutic resistance and tumor relapse.^240,241^ In contrast, spatial and temporal heterogeneity describe how tumor characteristics vary across different regions and over time. Spatial heterogeneity refers to differences in the TME. Different regions of the tumor exhibit variations in oxygenation, blood supply, and immune cell infiltration. These factors influence immune suppression and tumor growth. Temporal heterogeneity refers to how tumor properties evolve as the tumor progresses. Changes in the immune microenvironment and mutations allow the tumor to continuously evade immune detection, often by upregulating immune checkpoint pathways.^242,243^ While ITH focuses on cellular diversity within the tumor, spatial and temporal heterogeneity address how location and changes over time influence immune responses. Together, both concepts highlight the complexity of immune evasion in cancer (Fig. 4). They underscore the challenge of developing effective therapies that can target both tumor cell diversity and dynamic immune interactions within the TME.

### Intra-tumor heterogeneity

Intra-tumor heterogeneity is a hallmark of cancer that significantly complicates the treatment landscape, particularly concerning the immune system’s ability to recognize and eliminate malignant cells.^244^ This diversity within the TME creates a formidable challenge for the immune system and contributes to immune evasion.

One of the primary mechanisms by which ITH facilitates immune evasion is through the differential expression of tumor antigens across various subclones within the tumor.^245^ As cancer cells evolve, they accumulate mutations, leading to the generation of neoantigens—novel proteins that the immune system can identify as foreign. However, due to ITH, not all cancer cells within the tumor express the same set of neoantigens. This mosaic of antigen presentation allows some subclones to escape immune detection and destruction. For instance, immune cells may successfully target and eliminate subpopulations of cells presenting specific neoantigens while leaving other subclones, which do not express these neoantigens. Selected pressure can cause the expansion of immune-resistant clones, which contributes to tumor development and treatment resistance.^244,246^ For example, a recent study demonstrates the potential of AI-based image analysis in predicting platinum-based chemotherapy resistance in ovarian cancer. By automatically extracting image features from hematoxylin and eosin-stained tissue sections, researchers identified perimeters as a key feature associated with ITH. ITH, in turn, correlated with drug resistance.^247^

Moreover, ITH influences TME in ways that further enhance immune evasion. The diverse subpopulations within a tumor can secrete different cytokines and growth factors, shaping the TME to be more immunosuppressive. For example, certain subclones may secrete elevated levels of immunosuppressive cytokines, which hinder the function of cytotoxic T cells and promote the recruitment of Tregs and MDSCs. These changes create a local environment that is hostile to effective immune responses, thereby protecting the tumor from immune-mediated destruction.^248,249^ Additionally, the phenotypic plasticity of cancer cells within a heterogeneous tumor allows for the adoption of traits that further enhance immune evasion, such as upregulation of immune checkpoint molecules or downregulation of MHC molecules, which are critical for antigen presentation.^248,250^

The dynamic nature of ITH complicates predictions of immunotherapy efficacy. Immune checkpoint inhibitors, which have transformed cancer therapy, rely on the immune system’s ability to recognize tumor antigens. However, due to the variability in antigen expression driven by ITH, these therapies may be less effective against tumors with high levels of heterogeneity. Some subclones within the tumor may not express the target antigens at all, rendering them invisible to the immune system despite the presence of immunotherapeutic agents.^251^ Furthermore, as immunotherapies exert selective pressure on the tumor, they may inadvertently promote the survival and expansion of resistant subclones, leading to relapse and disease progression.^252,253^ For example, a recent study highlights the critical role of classical Ly6C+ monocytes in ICI resistance. The authors demonstrate that these monocytes can differentiate into immunosuppressive cells, contributing to resistance to anti-PD-1/CTLA-4 combination therapy. Targeting classical Ly6C+ monocytes with an anti-Ly6C antibody effectively reverses resistance and restores anti-tumor T cell activity.^252^ Another study describes a new tumor-microenvironment-on-chip (TMoC) technology that accurately captures the complex, diverse character of breast cancer TME. By combining a circulatory system, ex vivo tissue culture, and physiological gradients, the TMoC allows researchers to examine the efficiency of combination immunotherapies in a highly predictive manner. The platform’s capability to mimic the spatial interactions between tumor cells, immune cells, and drug gradients makes it a significant tool for drug discovery and development, bringing insights into the mechanisms of resistance and viable approaches to overcome them.^253^

### Spatial and temporal heterogeneity

Tumor heterogeneity plays a crucial role in cancer progression, treatment resistance, and immune evasion.^254,255^ Spatial and temporal heterogeneity are particularly important in shaping how tumors interact with the immune system.^256^ These forms of heterogeneity introduce complexity into the TME and cancer evolution, making it difficult for the immune system to effectively target and eradicate malignant cells.

Spatial heterogeneity creates a distinct microenvironment with unique immune landscapes. Some regions of the tumor may harbor highly immunogenic cancer cells, which express abundant neoantigens and are more susceptible to immune attack.^257,258^ However, other regions may contain subpopulations that have downregulated antigen presentation machinery or have upregulated immune checkpoint molecules, enabling these cells to avoid immune surveillance. As a result, the immune system’s capability to mount an effective and uniform response is compromised, as it must contend with a patchwork of immune-resistant regions within the same tumor.^259,260^

Moreover, spatial heterogeneity in tumors leads to areas rich in cytotoxic T cells and others dominated by immunosuppressive cells, enhancing immune evasion.^257,259^ For example, in nasopharyngeal carcinoma, markers for Tregs and suppressive myeloid cells were higher in “surrounding stromal leukocyte” regions compared to “immune-rich cancer cell islets”.^261^ Furthermore, regionally diverse expression of cytokines and chemokines can influence immune cell recruitment and activity, leading to a highly variable immune microenvironment across the tumor. This spatially driven immune landscape enables certain cancer cell populations to thrive in immune-privileged niches, contributing to the persistence and growth of the tumor. For example, a recent study, which modeled heterogeneous tumors with distinct pro-inflammatory (“hot”) and immunosuppressive (“cold”) populations, revealed that the spatial distribution of these tumor cell types significantly impacts the infiltration and activity of immune cells. It was observed that “hot” tumor regions, marked by YFP expression, attracted more T cells, including Th1 cells and IFN-γ + CD8 T cells, compared to “cold” regions tagged with RFP. Conversely, immunosuppressive macrophages were more abundant in “cold” areas, with CX3CL1 identified as a key chemokine mediating their accumulation, particularly the CD206Hi subset. Despite the ability of the combination therapy of PD-1 blockade and CD40 agonist to enhance Th1 cell presence in “cold” regions, the overall T cell activity remained suboptimal compared to “hot” regions, resulting in an inability to induce tumor rejection.^259^

Temporal heterogeneity, on the other hand, refers to the dynamic changes in the genetic and phenotypic profiles of cancer cells over time. As the tumor evolves, cancer cells continuously acquire new mutations, undergo epigenetic modifications, and adapt to selective pressures imposed by the immune system and therapeutic interventions. This temporal evolution can result in the emergence of new subclones that are more adept at evading immune detection.^255^ For example, cancer cells that initially present strong neoantigens may undergo mutations that lead to the loss or modification of these antigens, making them less detectable by the immune system.^1,153^ Alternatively, cancer cells may increase the expression of immune checkpoint molecules, such as PD-L1, in response to immunological pressure, therefore suppressing T cell function and encouraging immune escape.^262^ For example, recent research revealed that the modulation of PD-L1 expression plays a significant role in tumor growth and immune evasion across diverse cancer types, including HNSCC and HCC. In HNSCC, PD-L1 overexpression is driven by the IFN-γ/JAK2/STAT1 signaling pathway and modulated by circ_0000052, which sponges miR-382-3p, thereby enhancing PD-L1 levels and promoting malignancy.^263^ Similarly, in HCC, the lncRNA NRIR is highly expressed and promotes immune escape by upregulating PD-L1 expression through the IFN-γ signaling pathway. This upregulation of PD-L1 contributes to tumor progression by inhibiting effective T cell-mediated immune responses.^264^ Additionally, DKK1 upregulates PD-L1 in HCC through Akt/β-catenin signaling, decreasing CD8+ T cell infiltration and promoting tumor growth.^265^

Furthermore, the temporal aspect of tumor heterogeneity also affects the efficacy of immunotherapies. As tumors evolve over time, they can develop resistance to immune checkpoint inhibitors or other forms of immunotherapy.^266^ This resistance may arise from the selection of immune-resistant subclones or from adaptive changes in the TME that reduce the effectiveness of the immune response.^267,268^ For instance, treatment with immune checkpoint inhibitors may initially lead to tumor regression, but over time, resistant subclones may emerge, leading to disease relapse. These subclones may have evolved mechanisms to escape immune detection, such as altered antigen presentation, increased immunosuppressive signaling, or enhanced survival pathways.^269^

## Signaling pathway and immune evasion in cancer

### PD-1/PD-L1 pathway

**Fig. 5:**
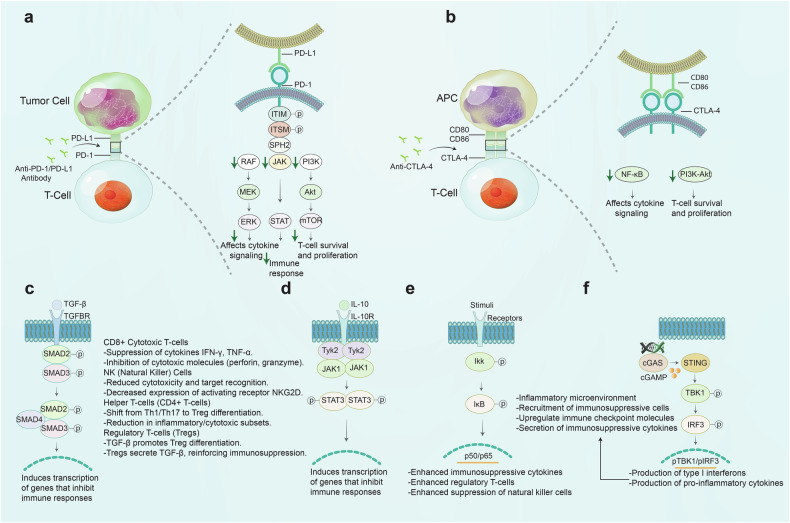
Pathways associated with the immune evasion of cancer. **a** The PD-1/PD-L1 pathway in immune evasion of cancer illustrates how binding between PD-1 receptors on T cells and PD-L1 on cancer cells triggers inhibitory signaling that suppresses T cell functions. This interaction recruits SHP-2 phosphatase to PD-1’s ITIM and ITSM motifs, which dephosphorylates key signaling molecules, inhibiting the PI3K-Akt, RAS-MAPK, and JAK-STAT pathways. This suppression decreases cytokine production, cytotoxic activity, and T cell proliferation and survival, leading to T cell exhaustion. Together, these effects contribute to immune evasion, tumor growth, and potential metastasis due to impaired immune surveillance in the TME. **b** CTLA-4 Pathway in immune evasion of cancer shows how CTLA-4 suppresses T cell activation to aid immune evasion. T cell activation typically requires CD28 binding to B7 ligands on APCs. However, CTLA-4, with its higher affinity for B7, outcompetes CD28, leading to reduced T cell activation. This CTLA-4-B7 binding suppresses intracellular signaling pathways in T cells, including the PI3K-Akt and NF-κB pathways, resulting in decreased T cell proliferation, reduced cytokine production, and impaired immune response. The outcome is immune evasion, allowing tumor cells to survive and proliferate. **c** TGF-β is secreted by various cells, binding to TGF-β receptors on immune cells. This interaction initiates intracellular signaling cascades, particularly through the SMAD pathway, leading to gene transcription that suppresses immune functions. Key effects include reduced production of pro-inflammatory cytokines, diminished cytotoxic molecule release, and a shift in T cell differentiation toward immunosuppressive Tregs, collectively weakening the immune response. **d** The IL-10 binds to IL-10 receptors. This binding activates the JAK1/STAT3 pathway, leading to the transcription of immunosuppressive genes that reduce immune responses. In CD8+ T cells, IL-10 signaling suppresses cytokine production and cytotoxic molecules, weakening their ability to attack cancer cells. In APCs, IL-10 impedes maturation, decreases antigen presentation, and downregulates co-stimulatory molecules, thereby limiting T cell activation. For NK cells, IL-10 reduces cytotoxic activity and receptor expression, diminishing their tumor-targeting function. IL-10 also promotes Treg differentiation, creating a feedback loop of immunosuppression that supports tumor growth and metastasis. **e** The NF-κB Pathway in immune evasion of cancer demonstrates how NF-κB activation within both tumor and immune cells promotes immune suppression. In cancer cells, NF-κB signaling induces pro-survival and proliferative genes, along with pro-inflammatory cytokines, fostering a tumor-supportive microenvironment. In immune cells, NF-κB drives the expression of immunosuppressive cytokines, recruiting Tregs and myeloid-derived suppressor cells, which reduce T cell and NK cell activity. Together, these effects enable immune evasion, supporting tumor growth and metastasis. **f** The figure illustrates the cGAS-STING pathway in immune evasion of cancer, highlighting key molecular and cellular events. Tumor cells release cytosolic DNA due to genomic instability or necrosis, which is sensed by cGAS, leading to the production of cGAMP. cGAMP binds to and activates STING, where it initiates downstream signaling, including the activation of TBK1 and phosphorylation of IRF3. This cascade promotes the production of type I interferons and pro-inflammatory cytokines, which can recruit immune cells. However, STING activation also contributes to immune evasion by inducing an inflammatory microenvironment, the recruitment of immunosuppressive cells, the upregulation of immune checkpoint molecules, and the secretion of immunosuppressive cytokines

The PD-1 receptor and its ligand, PD-L1, form a crucial immune checkpoint pathway that plays a significant role in immune evasion.^270,271^ The PD-1/PD-L1 pathway is a crucial regulatory mechanism that maintains immunological homeostasis, preventing excessive immune activation and autoimmunity.^272^ However, cancers frequently use this pathway to escape immune monitoring, contributing to tumor development and resistance to treatments.^273^ Understanding the PD-1/PD-L1 signaling pathway is crucial for developing targeted therapies aimed at enhancing anti-tumor-immune responses (Fig. 5a).

Tumors use the PD-1/PD-L1 pathway to protect themselves from immune attack.^57,273^ Many tumors upregulate PD-L1 expression due to inflammatory signals, which are produced by activated T cells.^273^ This PD-L1 overexpression generates an immunosuppressive milieu, inhibiting T cell activation even when tumor antigens are present. For example, a recent study found that combining sorafenib with doxorubicin effectively suppressed tumor growth in osteosarcoma. Sorafenib reduced doxorubicin-induced PD-L1 upregulation, enhancing the anti-tumor-immune response by increasing interferon-γ-secreting CD8+ T lymphocytes.^274^ Moreover, the interaction between PD-1 on T cells and PD-L1 on tumor cells effectively “turns off” T cells, allowing the tumor to evade immune destruction.^273,275^ This mechanism is particularly insidious because it hijacks a natural regulatory process intended to prevent tissue damage and autoimmunity, thereby creating a shield that protects the tumor from immune-mediated elimination.

The significance of this pathway in immune evasion is underscored by the success of immune checkpoint inhibitors that target this pathway.^276^ These inhibitors block PD-1 and PD-L1 interaction.^57^ Inhibiting this pathway allows T cells to target and destroy tumor cells, boosting anti-tumor immunity.^57,277^ These therapies’ effectiveness in cancers like melanoma, NSCLC, and renal cell carcinoma highlights the key role of this pathway in tumor-immune evasion.^278^

However, Tumors with high PD-L1 expression and mutational burden respond better to PD-1/PD-L1 blockers due to recognizable neoantigens.^57,279^ Additionally, Pre-existing TILs in the TME often correlate with a strong response to PD-1/PD-L1 blockade, indicating these therapies may work best in tumors already recognized by the immune system but suppressed by the PD-1/PD-L1 pathway. For instance, recent advances in neoadjuvant chemoimmunotherapy have improved outcomes in NSCLC. Unlike chemotherapy alone, neoadjuvant chemotherapy significantly increases the infiltration of CD4+ and CD8+ T cells—particularly CD8 + CD127+ and CD8+KLRG1+ phenotypes—as well as CD20+ B cells. These immune cells localize in close proximity within the TME, suggesting a cooperative interaction that contributes to improved therapeutic outcomes.^280^ Furthermore, single-cell studies reveal that major pathologic responders exhibit enhanced MHC class II-mediated antigen presentation in cancer cells alongside expansion of FCRL4 + FCRL5+ memory B cells and CD16 + CX3CR1+ monocytes, while non-responders demonstrate elevated estrogen metabolism and serum estradiol levels. Effective responses correlate with activated cytotoxic T cells, CD16 + NK cells, and LAMP3+ dendritic cells, coupled with reduced immunosuppressive Tregs and aged CCL3+ neutrophils.^281^ Spatial analyses highlight close interactions between CD127+/KLRG1 + CD8+ T cells and B/CD4+ T cells, with T follicular helper cells further amplifying anti-tumor immunity through IL-21-driven B cell IgG1/IgG3 responses.^282^ Even with the clinical success of PD-1/PD-L1 inhibitors, resistance remains a significant challenge.^283^ Tumors may acquire resistance through multiple mechanisms, such as the loss of antigen presentation machinery or the activation of alternative immunological checkpoints, or by creating a highly immunosuppressive microenvironment.^283,284^ Understanding these resistance pathways is critical for creating combination therapies capable of defeating resistance and improving patient outcomes. As a result, the PD-1/PD-L1 pathway is a critical mechanism of immune evasion in cancer, allowing tumors to avoid immune surveillance by inhibiting T cell function. The emergence of treatments targeting this pathway has transformed cancer treatment, providing considerable advantages to patients with a variety of malignancies. However, the complexity of immune evasion strategies employed by tumors necessitates ongoing research to refine these therapies and overcome resistance.

### CTLA-4 pathway

The CTLA-4 pathway is vital for immune balance and preventing autoimmunity.^285^ However, tumors often exploit the CTLA-4 pathway to evade immune detection. This pathway regulates the initial phases of T cell activation, limiting the immune system’s capacity to mount effective anti-tumor responses. Understanding the CTLA-4 signaling pathway and its role in immune evasion is vital for developing targeted immunotherapies aimed at enhancing the body’s natural defenses against cancer (Fig. 5b).

CTLA-4 acts as an inhibitory receptor. It competes with CD28 for binding to CD80/CD86 on APCs. CD28 binding provides a co-stimulatory signal for T cell activation, while CTLA-4 binding sends an inhibitory signal, reducing T cell activity.^286,287^ This inhibition occurs through multiple mechanisms, including the sequestration of CD80/CD86 away from CD28, reducing co-stimulatory signaling, and the recruitment of phosphatases like SHP-2 and PP2A, which dephosphorylate key signaling proteins involved in T cell activation. For instance, sCTLA-4, a soluble form of CTLA-4, has been identified as a significant modulator of anti-tumor immune responses. sCTLA-4 gene variations may be useful biomarkers for anti-CTLA-4 therapy and immune-related adverse effects. Although circulating blood levels of sCTLA-4 have been studied, tumor-specific mRNA expression remains unexplained. RNA-seq data reveal that while the membrane-bound form of CTLA-4 is more abundant in lung adenocarcinoma and melanoma, sCTLA-4 is correlated with immune suppression. Functional studies have shown that sCTLA-4 suppresses T cell proliferation, enhances tumor resistance to immune-mediated killing, and promotes tumor growth in vivo, highlighting its promise as a therapeutic target.^288^

Moreover, CTLA-4 regulates early T cell activation in lymphoid organs, preventing excessive activation and autoimmunity.^286,289^ It achieves this by controlling autoreactive T cell expansion,^286^ regulating B-1a B cells to prevent autoimmunity,^290^ and controlling T cell migration in conjunction with PD-1.^291^ These insights emphasize the central role of CTLA-4 in maintaining immune homeostasis and avoiding autoimmunity. However, tumors exploit this pathway to suppress anti-tumor immunity. In the TME, the expression of CTLA-4 on T cells, especially Tregs, can be upregulated, resulting in a decline in the activation and proliferation of CTLs that are crucial for recognizing and destroying cancer cells.^292,293^ For example, a recent study demonstrates that combining radiation therapy and anti-CTLA-4 with an agonistic CD40 antibody can significantly enhance anti-tumor-immune responses in immune checkpoint blockade-refractory TNBC by overcoming CTLA-4-mediated suppression. This combination therapy activates dendritic cells to prime tumor-specific CD8 T cells, while blocking CTLA-4 on regulatory T cells prevents immune suppression, leading to improved treatment outcomes in previously resistant cancers.^294,295^ Additionally, the presence of Tregs, which highly express CTLA-4, further contributes to an immunosuppressive microenvironment by outcompeting effector T cells for CD80/CD86 binding, thereby inhibiting effective immune responses.^286,293^

The impact of CTLA-4 on immune evasion is further highlighted by the fact that tumors can actively recruit and expand Tregs within the TME. These Tregs, through their high expression of CTLA-4, not only suppress CTL activity but also modulate the function of APCs, reduce their ability to deliver antigens, and generate the essential co-stimulatory signals for T cell activation.^296,297^ This creates a feedback loop in which the immunosuppressive environment is maintained and even enhanced, allowing the tumor to grow and escape immune destruction.

Given the CTLA-4 pathway’s critical involvement in immune evasion, it has emerged as a key target for cancer immunotherapy. The emergence of CTLA-4 inhibitors, such as ipilimumab, signaled the beginning of immune checkpoint blockade therapy. Ipilimumab is a monoclonal antibody that suppresses CTLA-4, preventing it from attaching to CD80/CD86. This inhibition successfully increases T cell activation by allowing CD28 to interact with CD80/CD86 without interference from CTLA-4, resulting in a more robust anti-tumor immune response. Ipilimumab’s success, especially in the treatment of metastatic melanoma, highlights the therapeutic potential of targeting the CTLA-4 pathway to overcome immune evasion.^296,298^

However, the clinical application of CTLA-4 inhibitors is not without obstacles. The widespread activation of T cells resulting from CTLA-4 inhibition can lead to immune-related adverse events (irAEs), such as colitis, hepatitis, and endocrinopathies. This increased immune activity may inadvertently target normal tissues, causing these adverse effects.^299^ For example, a recent study examined data from the FDA Adverse Events Reporting System (FAERS) and AERSMine to identify the 25 most common adverse events (AEs) linked to FDA-approved ICIs, including PD-1, PD-L1, CTLA-4, and LAG-3 inhibitors. The findings show that diarrhea, fatigue, and pyrexia are frequent AEs across different ICIs, with specific inhibitors linked to unique AEs like neutropenia, hepatotoxicity, and biliary tract infection. Rare but serious AEs, including myocarditis and myasthenia gravis, were also observed.^300^ This demonstrates the challenging balance between boosting anti-tumor immunity and maintaining immunological tolerance, which must be carefully controlled in patients receiving CTLA-4 inhibitors.

Moreover, resistance to CTLA-4 blockade can occur through several mechanisms. Tumors may upregulate other immune checkpoints to compensate for the loss of CTLA-4-mediated inhibition, thereby maintaining immune suppression. Additionally, the complex interplay between different immune cell types within the TME, including Tregs, MDSCs, and TAMs, can sustain an immunosuppressive environment even in the presence of CTLA-4 inhibition.^301,302^ For example, a recent study demonstrates that combinatorial checkpoint blockade, particularly targeting PD-1, LAG-3, and CTLA-4, significantly enhances anti-tumor immunity in ovarian cancer by overcoming compensatory upregulation of immune checkpoints and promoting a robust T cell response, thereby providing a compelling rationale for this strategy in clinical interventions.^303^ Moreover, another study reveals a new role for CD4+Foxp3− T cells in boosting the anti-tumor immune response by modulating the myeloid compartment. They activate CD103+ dendritic cells and expand TNF-α and iNOS-producing myeloid subsets, enhancing the therapeutic efficacy of dual PD-1/CTLA-4 blockade in cancer immunotherapy.^304^ Therefore, gaining insight into these resistance mechanisms is essential for optimizing the effectiveness of CTLA-4 inhibitors and for developing combination therapies that can more effectively target multiple immune evasion pathways.

### TGF-β pathway

The TGF-β pathway has a dual role in cancer, acting as a tumor suppressor in early stages and promoting tumor progression and immune evasion in later stages.^305^ TGF-β is a cytokine that affects multiple cellular processes, including proliferation, differentiation, apoptosis, and immunological responses. In cancer, the TGF-β pathway is frequently co-opted by tumors to create an immunosuppressive microenvironment that facilitates immune evasion (Fig. 5c), promoting tumor growth, metastasis, and resistance to therapies.^306,307^

TGF-β signaling activates TGFBR1 via TGFBR2, leading to R-SMADs forming a complex with SMAD4 to regulate genes involved in cell cycle arrest and apoptosis.^308^ In the early stages of cancer, TGF-β signaling exerts a tumor-suppressive effect by inducing growth arrest and apoptosis in epithelial cells. However, as the tumor progresses, cancer cells often develop resistance to these effects of TGF-β and instead exploit the pathway to promote malignant behavior. For example, a recent study emphasized TGF-β‘s significant role in resistance to ICIs in gynecological cancers. Lower TGF-β scores correlate with better survival. Immune-related toxicity predicts ICI response, while PD-L1 relates to poorer outcomes. Immunosuppressive cells are linked to decreased survival, suggesting TGF-β scores may indicate ICI treatment failure risk.^309^

TGF-β has a vital function in cancer progression by modulating the immune system. TGF-β suppresses immune function, allowing tumors to avoid detection and elimination. TGF-β is secreted in the TME by cancer cells and stromal cells.^17,310^ High TGF-β levels suppress CTLs and NK cells by reducing NKG2D ligands in lung cancer, an effect reversed by Galunisertib. Inhibiting TGF-β may enhance NK cell responses by boosting NKG2D ligand expression.^311^

In addition to its direct effects on effector immune cells, TGF-β increases the differentiation and growth of immunosuppressive cells within the TME. TGF-β promotes the development of naïve CD4+ T cells into Tregs, which maintain immunological tolerance and suppress anti-tumor immunity.^312^ For example, TGF-β signaling is crucial for the early thymic development of natural CD4+CD25+Foxp3+Tregs, as evidenced by the absence of these cells after conditional deletion of the TGF-β receptor I in T cells. However, without TGF-β signaling, the expansion of CD4+CD25+Foxp3+ thymocytes is supported by increased IL-2 production and responsiveness. This indicates a compensatory mechanism that maintains Treg populations both in the thymus and in peripheral tissues.^313^ TGF-β also enhances the immunosuppressive functions of TAMs and MDSCs, which contribute to the inhibition of CTLs and NK cells and support tumor progression through the secretion of pro-tumorigenic factors.^312,314^

Moreover, TGF-β signaling is associated with EMT. During EMT, cancer cells lose their epithelial properties and develop a more mesenchymal, migratory phenotype, which boosts their ability to invade neighboring tissues and spread to distant regions.^315,316^ This process is mediated through various signaling pathways, primarily the canonical TGF-β/SMAD pathway, which activates transcription factors that promote the EMT program.^316^ For example, a recent study reported that TGF-β-dependent signaling plays a central role in promoting EMT during advanced stages of BC. A comprehensive, literature-curated regulatory map of TGFβ-induced EMT was constructed using Systems Biology Graphical Notation (SBGN) via CellDesigner. The map includes 312 molecular entities and 426 interactions encompassing state transitions, complex formations, and translocations. This network was further converted into a Boolean model using CaSQ and simulated through Cell Collective, effectively recapitulating experimentally observed EMT behaviors. Key regulatory hubs identified from the map were validated through transcriptomic analysis, underscoring their relevance in BC metastasis.^317^ However, ALKBH5, an RNA m6A demethylase, has shown promise in suppressing TGF-β-induced EMT and metastasis in NSCLC by altering the stability and expression of critical components in the TGF-β/SMAD signaling pathway. Overexpression of ALKBH5 decreases TGFβR2 and SMAD3 expression and mRNA stability, while increasing SMAD6, resulting in reduced TGF-β signaling, EMT, and invasion of NSCLC cells. This regulatory function of ALKBH5 is mediated through its influence on m6A modifications, highlighting its ability as a therapeutic target in controlling NSCLC progression and metastasis.^318^ TGF-β is a potent inducer of EMT, and through this process, it not only promotes metastasis but also contributes to immune evasion by reducing the expression of epithelial antigens that could be recognized by the immune system.

TGF-β has a key role in immune evasion and cancer progression, making it a promising therapeutic target. To reduce TGF-β signaling in cancer, small-molecule inhibitors, monoclonal antibodies, and antisense oligonucleotides targeting ligands, receptors, or downstream signaling components are being investigated. By blocking TGF-β signaling, these therapies aim to restore the anti-tumor activity of immune cells and reverse the immunosuppressive environment within the TME. However, targeting the TGF-β pathway is challenging due to its pleiotropic effects and its involvement in normal physiological processes, which necessitates careful consideration of potential side effects.

### IL-10 pathway

The IL-10 pathway is a critical immunoregulatory mechanism that plays a complex role in cancer by modulating immune responses. IL-10 is a cytokine primarily known for its anti-inflammatory properties, which are essential in preventing excessive immune activation and maintaining immune homeostasis.^27,319^ However, in cancer, the IL-10 pathway is frequently exploited by tumors to suppress anti-tumor immunity (Fig. 5d), thereby promoting immune evasion and facilitating tumor progression.^28,320^ Understanding IL-10’s dual role in immune regulation and immune evasion is critical for developing treatment techniques that can modify this pathway to boost anti-cancer immunity.

IL-10 is yielded by a variety of immune cells, such as regulatory Tregs, B cells, macrophages, DCs, and certain subsets of T helper cells.^321^ After secretion, IL-10 exerts its effects by attaching it to the IL-10 receptor (IL-10R). The IL-10R is a heterodimer consisting of IL-10R1 and IL-10R2 subunits, and its activation stimulates the JAK-STAT signaling cascade, specifically involving the phosphorylation and activation of STAT3.^322,323^ Once activated, STAT3 translocates to the nucleus and activates the transcription of target genes that mediate the immunosuppressive effects of IL-10. Notably, the activation of STAT3 is crucial for tumor growth as it enhances the expression of PD-L1 on tumor cells. The interaction between PD-L1 and PD-1 on T cells diminishes T cell function, allowing tumors to evade immune surveillance and contributing to immune evasion.^324,325^ Additionally, STAT3 induces the production of VEGF,^193^ promoting angiogenesis and contributing to tumor growth while also having immunosuppressive effects by inhibiting dendritic cell maturation. Interestingly, STAT3 further enhances IL-10 production, creating a positive feedback loop that sustains an immunosuppressive environment by inhibiting various immune cells, including macrophages and dendritic cells.^326^ Furthermore, STAT3 activation recruits and activates MDSCs, known for suppressing T cell responses and facilitating tumor progression.^193^

In the TME, the IL-10 pathway aids immune evasion by inhibiting APC maturation and activity, limiting dendritic cells’ ability to present tumor antigens and hindering adaptive immune responses.^327,328^ Furthermore, IL-10 decreases the production of MHC class II and co-stimulatory molecules on APCs, impairing their ability to activate T cells.^328^ As a result, the anti-tumor-immune response is compromised, allowing tumor cells to escape immune detection and destruction.

IL-10 also exerts profound effects on T cells within the TME. It prevents the proliferation and cytokine production of CTLs, which are crucial for directly killing tumor cells. By reducing the production of pro-inflammatory cytokines, such as IFN-γ and TNF-α, IL-10 diminishes the effector functions of CTLs, limiting their ability to control tumor growth. Furthermore, IL-10 promotes the expansion and function of Tregs, which are key players in maintaining immune tolerance and suppressing anti-tumor immunity. Tregs produce high levels of IL-10, creating a feedback loop that reinforces the immunosuppressive environment within the TME.^329,330^

Another critical aspect of IL-10’s role in immune evasion is its impact on MDSCs and TAMs. IL-10 enhances the immunosuppressive functions of these cells, which are abundant in the TME and contribute to the inhibition of CTLs and NK cells.^331,332^ In particular, TAMs often polarize into an M2-like phenotype in response to IL-10, characterized by the release of anti-inflammatory cytokines, growth factors, and enzymes that promote tumor growth, angiogenesis, and metastasis. This polarization inhibits pro-inflammatory responses and stimulates tissue remodeling, fostering a supportive environment for tumor development.^333,334^

While IL-10’s immunosuppressive actions in the TME contribute to immune evasion, it is important to consider that IL-10 can also be anti-tumor under certain situations. For example, IL-10 might boost the cytotoxic activity of NK cells in some situations, especially when it is present at low amounts. Furthermore, IL-10 inhibits the synthesis of pro-tumorigenic cytokines, which are involved in chronic inflammation and cancer growth.^28,321^ This dual role of IL-10 underscores the complexity of its function in cancer and highlights the need for context-specific approaches when targeting the IL-10 pathway for therapeutic purposes.

Given the critical role of the IL-10 pathway in immune evasion, there is significant interest in developing strategies to modulate this pathway in cancer therapy. One approach involves the use of IL-10 inhibitors, which aim to block the immunosuppressive effects of IL-10 and restore anti-tumor immunity.^328^ However, because IL-10 also plays a role in controlling inflammation and preventing autoimmunity, careful consideration is required to avoid unintended consequences, such as exacerbating inflammation or triggering autoimmune reactions. Alternatively, some therapeutic strategies seek to harness the anti-tumor potential of IL-10 by delivering it in a controlled manner or in combination with other immune-stimulating agents to selectively enhance its beneficial effects while minimizing its immunosuppressive impact.

### NF-κB pathway

The NF-κB pathway is a complex signaling cascade involved in cancer genesis and progression. NF-κB transcription factors regulate gene expression for inflammation, immunological response, cell survival, and proliferation. In cancer, dysregulation of the NF-κB pathway can cause chronic inflammation, resistance to apoptosis, and immune evasion (Fig. 5e). Understanding the mechanisms by which the NF-κB pathway contributes to immune evasion provides valuable insights into its role in cancer. It offers potential therapeutic targets for improving anti-cancer immunity.^335,336^

NF-κB consists of five subunits: RelA, RelB, c-Rel, p50, and p52. These subunits create homo- or heterodimers that can translocate to the nucleus and bind to certain DNA regions, regulating gene transcription.^337,338^ The NF-κB pathway consists of two main signaling pathways: canonical and non-canonical. The canonical pathway is activated by pro-inflammatory cytokines, as well as pathogen-associated molecular patterns (PAMPs) recognized by TLRs.^339^ The non-canonical pathway is primarily triggered by TNF receptor superfamily members, including CD40 and lymphotoxin-β receptors.^339^

In the TME, the NF-κB pathway is often constitutively activated, which contributes to several hallmarks of cancer, including immune evasion. NF-κB promotes immune evasion primarily by regulating pro-inflammatory cytokines and chemokines that influence the TME. Chronic NF-κB activation results in the production of cytokines like IL-6, TNF-α, and IL-1β, creating a persistent inflammatory environment. This chronic inflammation can suppress anti-tumor immunity and aid tumor progression by recruiting and activating immunosuppressive cell types within the TME.^340^ This inflammatory environment attracts immunosuppressive cells, including Tregs, MDSCs, and TAMs, all of which contribute to the inhibition of CTLs and NK cells.^341^

Moreover, NF-κB activation in cancer cells boosts PD-L1 production, which binds to PD-1 on T cells, causing exhaustion and reducing T cell proliferation and anti-tumor efficacy. By promoting PD-L1 expression, NF-κB contributes to the mechanisms of immune evasion, allowing tumors to escape immune surveillance and progress unchecked. NF-κB enhances PD-L1 expression, creating an immunosuppressive barrier surrounding tumors and allowing cancer cells to escape immune monitoring.^342^ Additionally, NF-κB promotes the production of immunological checkpoint molecules, inhibiting T cell activation and activity inside the TME. For example, a previous study has suggested that B7-H4 overexpression on monocytes and macrophages in gastric cancer patients is associated with immune evasion and tumor progression. Elevated B7-H4 levels are linked to deeper tumor invasion, lymphatic and venous spread, and immunosuppressive effects on CD4+ T cells.^343^

The NF-κB pathway also promotes tumor cell survival and resistance to apoptosis, indirectly facilitating immune evasion. NF-κB facilitates the expression of anti-apoptotic genes, such as Bcl-2, Bcl-xL, and IAP proteins. This helps cancer cells avoid cell death caused by immune cells or chemotherapy drugs.^339,344^ This resistance to apoptosis allows cancer cells to remain and grow, even in the presence of an active immune response, helping to the overall evasion of immune-mediated destruction.^345^

In addition to its effects on immune cells, NF-κB signaling affects the behavior of non-immune cells in the TME, including fibroblasts and endothelial cells. Activation of NF-κB in these cells promotes angiogenesis, tissue remodeling, and metastasis by secreting growth factors and MMPs.^346^ These events not only promote tumor growth but also complicate the immunological landscape, hindering the immune system’s ability to target and eliminate cancer cells.

Given NF-κB’s central role in cancer-related inflammation, immune suppression, and cell survival, it has become a key target in cancer therapy. Strategies to inhibit NF-κB signaling include small-molecule inhibitors targeting components like IKK or NIK and drugs designed to disrupt NF-κB’s interaction with target DNA sequences, thereby preventing the transcription of pro-tumorigenic genes. However, targeting NF-κB is challenging because of its important involvement in immune function and tissue homeostasis. Systemic inhibition may lead to side effects such as increased infection risk and poor wound healing. Therefore, research is focused on therapeutic approaches that selectively target NF-κB in cancer cells or the TME while sparing healthy tissues.

### cGAS-STING pathway

The cGAS-STING pathway, a critical innate immune response mechanism, can be hijacked by tumors to evade immune detection by suppressing the activation of immune signaling, thus enabling cancer cells to escape immune surveillance and promote tumor progression (Fig. 5f). The cGAS-STING pathway detects cytosolic DNA, a sign of infection or cell damage. Normally, DNA stays in the nucleus or mitochondria. However, during stress or cell death, DNA can leak into the cytoplasm. When cGAS senses this DNA, it produces cGAMP, a secondary messenger. cGAMP binds to STING on the endoplasmic reticulum, activating a signaling cascade. STING moves to the Golgi and activates TBK1.^347,348^ TBK1 phosphorylates IRF3, which then triggers the production of type I interferons and cytokines. These molecules recruit immune cells to fight infections or tumors.^349^

In cancer, the cGAS-STING pathway detects DNA released from tumor cells undergoing apoptosis or necrosis. This DNA triggers immune activation through type I interferons and cytokines. These molecules enhance tumor visibility to the immune system. They also activate dendritic cells, NK cells, and cytotoxic T cells.^350,351^ This process makes the tumor more immunogenic and vulnerable to immune attack. However, many tumors have developed strategies to evade this immune detection by manipulating the cGAS-STING pathway. One common mechanism is the loss or mutation of cGAS or STING, which prevents the tumor cells from activating the immune response even when cytosolic DNA is present. Some cancers have mutations in the STING gene, rendering the pathway inactive and allowing the tumor to escape immune surveillance. For example, the POLE P286R mutation in endometrial carcinoma enhances the cGAS-STING pathway. It increases cGAS levels and promotes TBK1 phosphorylation. This stimulation leads to the upregulation of inflammatory gene expression. As a result, there is a stronger anti-tumor immune response. The mutation also inhibits tumor growth. These effects counteract immune evasion in cancer.^352^ In addition to genetic mutations, tumor cells may express proteins that inhibit STING activation, such as USP18, which can also affect the stability and function of cGAS. For instance, in BRAF V600E mutant melanoma, USP18 stabilizes cGAS by deubiquitinating it, promoting its protein stability, and enhancing the resistance to vemurafenib. This interaction not only prevents proper immune activation through the cGAS-STING pathway but also supports tumor survival by inducing protective autophagy, thus contributing to immune evasion and therapeutic resistance.^353^

Moreover, some tumor cells may alter their DNA repair mechanisms to limit the release of DNA into the cytoplasm. By enhancing DNA repair pathways like Ataxia Telangiectasia and Rad3-related (ATR), tumors can reduce DNA fragmentation, making it less likely for the cGAS-STING pathway to be triggered. For example, ATR has emerged as a key target for overcoming resistance to PARP inhibitors in cancer, particularly ovarian cancer. Resistance to PARPi is often linked to the restoration of homologous recombination repair or the protection of stalled replication forks. In recent studies, ATR inhibition (ATRi) has been shown to resensitize PARPi-resistant cancer cells. This occurs by enhancing replication stress, which leads to increased replication fork stalling, double-strand breaks, and apoptosis. Combination therapies involving ATRi and PARPi have demonstrated synergistic effects in models of platinum- and PARPi-resistant OVCA. This includes models with BRCA1/2 reversion and CCNE1 amplification. ATRi has also shown potential in overcoming resistance in tumors that lack an underlying HRR deficiency.^354^ Furthermore, ATRi, particularly in combination with agents like WEE1 inhibitors, offers promising therapeutic strategies.^355^ Additionally, certain ncRNAs can regulate the expression of cGAS and STING, further inhibiting the immune response. For instance, in hypoxic tumors, hypoxia-responsive miRNAs like miR-25 and miR-93 suppress the cGAS pathway, leading to immune escape by preventing recognition of mitochondrial DNA.^356^ Similarly, in multiple myeloma, exosomal miRNAs transfer from tumor cells to host monocytes/macrophages, inhibiting the cGAS-STING antiviral immune response, which compromises innate immunity and increases viral susceptibility.^357^ Additionally, the lncRNA NEAT1 promotes tumor progression by interacting with DNMT1 to suppress the cGAS/STING pathway and hinder cytotoxic T cell infiltration.^358^ These ncRNAs contribute to immune evasion by manipulating immune detection mechanisms, supporting tumor survival and progression.

Given the pivotal role of the cGAS-STING pathway in initiating immune responses against tumors, there is growing interest in developing therapeutic strategies that either activate or enhance this pathway.^359^ STING agonists, such as synthetic cGAMP analogs or cyclic dinucleotides (CDNs), are being explored as potential treatments to stimulate the immune system. These agonists can activate the cGAS-STING pathway in immune cells, promoting an anti-tumor response.^360,361^ Combining STING agonists with immune checkpoint inhibitors, such as anti-PD-1 or anti-CTLA-4 therapies, may enhance the immune system’s ability to recognize and destroy tumors. Additionally, researchers are looking into strategies that target the immune evasion mechanisms used by tumors.

As research into the cGAS-STING pathway continues, it holds significant promise for cancer immunotherapy. By targeting the immune evasion strategies employed by tumors, we can enhance the immune response and improve the effectiveness of existing treatments. Ultimately, overcoming the obstacles that tumors pose to the cGAS-STING pathway could lead to more effective and targeted cancer therapies.

## Current therapeutic strategies targeting immune evasion

**Fig. 6:**
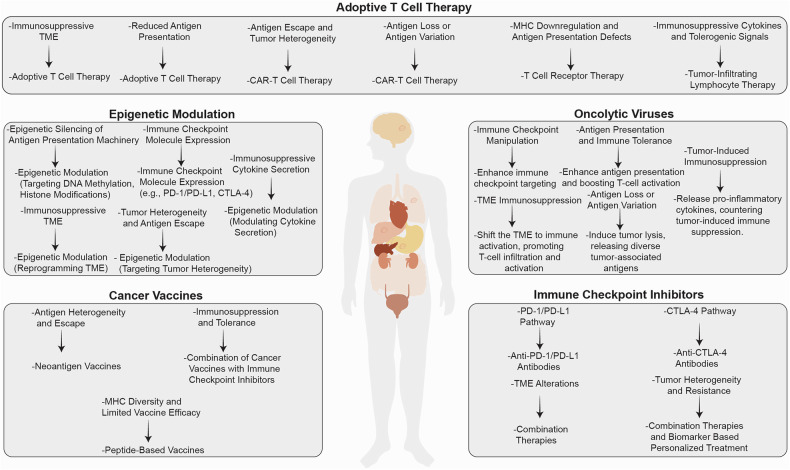
Therapeutic strategies to overcome immune evasion in cancer. Therapeutic strategies targeting immune evasion in cancer are summarized, highlighting five main approaches: immune checkpoint inhibitors, cancer vaccines, oncolytic viruses, adoptive T cell therapy, and epigenetic modulation. Immune checkpoint inhibitors restore T cell activation by targeting pathways like PD-1/PD-L1 and CTLA-4, but face challenges such as tumor heterogeneity and resistance. Cancer vaccines, including peptide-based and neoantigen vaccines, are often combined with checkpoint inhibitors but are limited by antigen heterogeneity and immunosuppressive mechanisms. Oncolytic viruses promote antigen presentation, cytokine release, and immune activation within the TME, while addressing challenges like antigen escape. Adoptive T cell therapies, including CAR-T cells and tumor-infiltrating lymphocytes, combat issues like antigen loss, MHC downregulation, and immunosuppressive signaling. Epigenetic modulation targets DNA methylation and histone modifications to reverse immune suppression, reprogram the TME, and restore antigen presentation. Together, these approaches tackle the complexities of immune evasion and aim to enhance cancer immunotherapy outcomes

There are multiple strategies for targeting immune evasion in cancer. Current therapeutic strategies targeting immune evasion include immune checkpoint inhibitors, cancer vaccines, oncolytic viruses, adoptive T cell therapy, and epigenetic modulation (Fig. 6). Immune checkpoint inhibitors block inhibitory signals that prevent T cell activation. Cancer vaccines aim to stimulate the immune system to recognize and destroy tumor cells. Oncolytic viruses selectively infect and kill cancer cells while boosting anti-tumor immunity. Adoptive T cell therapy involves modifying a patient’s T cells to enhance their ability to fight cancer. Finally, epigenetic modulation targets changes in gene expression to reinstate immune recognition of tumors. These approaches are reshaping cancer treatment by overcoming immune resistance.

### Immune checkpoint inhibitors

The ICIs are a game-changing innovation in cancer therapy, radically transforming the treatment landscape by leveraging the body’s immune system to battle malignancies. These treatments are intended to defeat cancer cells’ immune evasion mechanisms, including those involving immunological checkpoint molecules that ordinarily function as brakes on immune responses to maintain self-tolerance and prevent autoimmunity. By inhibiting these checkpoints, ICIs restore the ability of immune cells, particularly T cells, to recognize and kill cancer cells.^362,363^ Cancer cells frequently exploit immune checkpoints to evade immune detection. These checkpoints are mechanisms that tumors use to suppress T cell activity. By inhibiting these checkpoints, ICIs counteract the immune evasion strategies employed by cancer cells. This inhibition allows T cells to become activated, enhancing their ability to recognize and target cancer cells as threats.^55,364^

The two most well-characterized immune checkpoints targeted by ICIs are the CTLA-4,^365,366^ and the PD-1/PD-L1 pathways.^367^ These pathways play distinct but complementary roles in regulating immune responses, and their inhibition has led to significant clinical successes in treating various cancers. Recently, several trials focused on evaluating the safety, efficacy, and pharmacokinetics of various PD-1 and CTLA-4 inhibitors (Table 4). For example, ABBV-181, a humanized anti-PD-1 monoclonal antibody, was tested in combination with ROVA-T, an antibody-drug conjugate targeting DLL3, in small cell lung cancer (SCLC) patients. The trial found that the combination had an adequate safety profile, with common adverse events like fatigue, decreased appetite, and nausea, and showed preliminary efficacy with partial responses in some patients.^368^ Similarly, another phase 1 trial evaluated previously treated patients with advanced solid tumors. The trial demonstrated that ABBV-181 was well-tolerated, with an acceptable safety profile like other PD-1 inhibitors. Despite a low overall response rate, the study showed sustained PD-1 receptor saturation, and the pharmacokinetic (PK) data suggested a favorable dose regimen.^369^ BGB-A317, a humanized IgG4 anti-PD-1 antibody, showed promising results in a Phase I multicenter study. It demonstrated favorable safety with manageable adverse events, and early signs of anti-tumor activity were observed, with partial responses in some patients. The study also confirmed that BGB-A317’s pharmacokinetics were linear and not influenced by body weight, suggesting the possibility of fixed dosing in future trials.^370,371^ PDR001, another anti-PD-1 antibody, was studied in patients with nonfunctional neuroendocrine tumors (NETs). The results showed limited clinical activity, with modest responses observed in specific subgroups, such as atypical carcinoids, and highlighted the need for further research into biomarkers for better prediction of treatment outcomes.^372^ Lastly, MIW815 (ADU-S100), a synthetic cyclic dinucleotide that activates the STING pathway, was combined with spartalizumab, a PD-1 inhibitor, in a Phase Ib dose-escalation study. Preliminary results showed that the combination therapy had anti-tumor activity, particularly in PD-1–naive TNBC and PD-1–relapsed melanoma, with no dose-limiting toxicities (DLTs) observed, making it a promising combination for further clinical investigation.^373^

**Table 4:** Recent clinical trials involving PD-1 and CTLA-4 inhibitors

Inhibitor	NCT number	Phase	Status	Mechanism of action	Year
ABBV-181	NCT02988960	Phase 1	Active, not recruiting	It works by blocking the PD-1 receptor on T cells, preventing its interaction with PD-L1/PD-L2 ligands on tumor cells. This helps restore T cell function and enhances the immune system’s ability to target and destroy cancer cells.	2017–2025
	NCT03000257	Phase 1	Completed		2016–2022
	NCT03035279	Phase 1	Terminated		2017–2019
	NCT02539719	Phase 1	Terminated		2015–2019
	NCT03893955	Phase 1	Active, not recruiting		2019–2025
	NCT03818542	Phase 1	Terminated		2020–2020
	NCT03071757	Phase 1	Completed		2017–2022
	NCT04196283	Phase 1	Completed		2020–2022
	NCT03138408	Phase 1	Terminated		2017–2019
	NCT06158958	Phase 1	Recruiting		2024–2028
	NCT05599984	Phase 1	Recruiting		2022–2027
	NCT03639194	Phase 1	Completed		2018–2024
	NCT03821935	Phase 1	Recruiting		2019–2027
	NCT05005403	Phase 1	Recruiting		2021–2026
	NCT04807972	Phase 1/2	Terminated		2021–2024
	NCT06487559	Phase 1	Recruiting		2024–2027
	NCT06632951	Phase 2	Recruiting		2024–2028
	NCT05822752	Phase 2	Active, not recruiting		2023–2026
	NCT06236438	Phase 2/3	Recruiting		2024–2031
	NCT06109272	Phase 2/3	Recruiting		2024–2030
	NCT06628310	Phase 2	Recruiting		2024–2030
	NCT04306900	Phase 1	Completed		2020–2024
BGB-A317	NCT05494762	Phase 1	Active, not recruiting	It binds to the PD-1 receptor on T cells, blocking its interaction with PD-L1 and PD-L2 ligands on tumor cells. This inhibition enhances T cell-mediated immune responses, allowing the immune system to better target and eliminate cancer cells.	2022–2025
	NCT05644626	Phase 1	Withdrawn		2024–2025
	NCT05935098	Phase 1	Recruiting		2023–2025
	NCT06540066	Phase 1	Recruiting		2024–2027
	NCT06262581	Phase 2	Recruiting		2023–2025
	NCT05909904	Phase 2	Active, not recruiting		2023–2027
	NCT05253118	Phase 2	Recruiting		2022–2026
	NCT06430658	Phase 2	Recruiting		2024–2027
	NCT05116085	Phase 2	Active, not recruiting		2022–2027
	NCT05267054	Phase 2	Completed		2022–2024
	NCT05904496	Phase 1	Recruiting		2023–2026
	NCT05981703	Phase 1	Recruiting		2023–2027
	NCT06091943	Phase 1	Active, not recruiting		2023–2026
	NCT06235918	Phase 2	Recruiting		2023–2026
	NCT05604560	Phase 2	Recruiting		2023–2026
	NCT05445648	Phase 2	Active, not recruiting		2023–2026
	NCT06499350	Phase 1/2	Active, not recruiting		2024–2026
	NCT05614453	Phase 2	Withdraw		2023–2023
	NCT05609370	Phase 1/2	Recruiting		2023–2028
	NCT05517330	Phase 2	Recruiting		2022–2024
	NCT05659186	Phase 2	Recruiting		2022–2025
	NCT05789069	Phase 1	Recruiting		2023–2025
	NCT05622071	Phase 2	Recruiting		2023–2028
	NCT05877001	Phase 2	Recruiting		2023–2025
	NCT05577702	Phase 2	Active, not recruiting		2023–2026
	NCT05211232	Phase 3	Active, not recruiting		2022–2028
	NCT05238883	Phase 1	Recruiting		2022–2026
	NCT05586061	Phase 2	Not yet recruiting		2022–2024
	NCT06010303	Phase 2	Active, not recruiting		2023–2025
	NCT05407519	Phase 2	Recruiting		2022–2026
	NCT05798533	Phase 1	Recruiting		2023–2024
	NCT04914390	Phase 2	Recruiting		2023–2025
	NCT05536102	Phase 2	Recruiting		2022–2027
	NCT05516589	Phase 2	Recruiting		2022–2024
	NCT05189457	Phase 2	Active, not recruiting		2022–2028
	NCT05809895	Phase 2	Withdrawn		2023–2029
	NCT05526924	Phase 1	Recruiting		2023–2026
	NCT05366829	Phase 2	Recruiting		2022–2027
	NCT05791097	Phase 3	Withdrawn		2023–2027
	NCT05582265	Phase 3	Recruiting		2022–2030
PDR001	NCT05135845	Phase 2	Suspended	It works by blocking the PD-1 receptor on T cells, preventing its interaction with PD-L1 and PD-L2 ligands on tumor cells. This blockage restores T cell function, enhancing the immune system’s ability to recognize and attack cancer cells.	2022–2025
	NCT05201066	Phase 2	Recruiting		2023–2028
AGEN1884	NCT03495882	Phase 1/2	Completed	It works by blocking the CTLA-4 protein on T cells. This inhibition enhances T cell activation and proliferation, thereby improving the immune system’s ability to recognize and attack cancer cells.	2017–2022
	NCT03894215	Phase 2	Active, not recruiting		2019–2026
	NCT03411473	Phase 2	Terminated		2017–2019
	NCT04607200	Phase 2	Withdrawn		2021–2021
	NCT04430036	Phase 2	Terminated		2020–2022
	NCT02694822	Phase 1/2	Completed		2016–2022
	NCT05572970	N/A	Available		N/A
	NCT04028063	Phase 2	Recruiting		2020–2026
BMS-986218	NCT04301414	Phase 1	Active, not recruiting	BMS-986218 is a non-fucosylated anti-CTLA-4 monoclonal antibody that enhances anti-tumor immunity by blocking CTLA-4 and increasing antibody-dependent cell cytotoxicity.	2020–2025
	NCT04785287	Phase 1/2	Active, not recruiting		2021–2025
	NCT05169684	Phase 2	Completed		2022–2023
	NCT03110107	Phase 1/2	Terminated		2017–2024
Quavonlimab	NCT03179436	Phase 1/2	Completed	Quavonlimab is a monoclonal antibody that blocks the interaction of CTLA-4 with its ligands, CD80 and CD86, thereby enhancing T cell activation and anti-tumor immunity.	2017–2024
	NCT03516981	Phase 2	Active, not recruiting		2018–2025
	NCT04700072	Phase 1/2	Active, not recruiting		2021–2030
	NCT04740307	Phase 2	Active, not recruiting		2021–2026
	NCT04305041	Phase 1/2	Active, not recruiting		2020–2030
	NCT04895722	Phase 2	Active, not recruiting		2021–2027
	NCT05899049	Phase 3	Active, not recruiting		2022–2026
	NCT04736706	Phase 3	Active, not recruiting		2021–2026
	NCT04305054	Phase 1/2	Recruiting		2020–2030
	NCT04626518	Phase 1/2	Active, not recruiting		2020–2025
	NCT04626479	Phase 1/2	Active, not recruiting		2020–2026
	NCT04938817	Phase 1/2	Recruiting		2021–2029
Botensilimab	NCT06279130	Phase 2/3	Recruiting	Botensilimab is an Fc-enhanced anti-CTLA-4 antibody designed to boost both innate and adaptive anti-tumor immunity, particularly in “cold” tumors, by promoting T cell activation and depleting immunosuppressive regulatory T cells within the tumor microenvironment.	2024–2034
	NCT05571293	Phase 2	Recruiting		2023–2025
	NCT05377528	Phase 1	Active, not recruiting		2022–2027
	NCT06322108	Phase 2	Recruiting		2024–2030
	NCT04121676	Phase 1	Active, not recruiting		2019–2027
	NCT03860272	Phase 1	Recruiting		2019–2026
	NCT05529316	Phase 2	Active, not recruiting		2022–2028
	NCT06300463	Phase 2	Recruiting		2024–2027
	NCT05608044	Phase 2	Active, not recruiting		2023–2026
	NCT05928806	Phase 2	Active, not recruiting		2023–2025
	NCT05630183	Phase 2	Recruiting		2023–2024
	NCT05632328	Phase 2	Recruiting		2024–2027
	NCT06268015	Phase 2	Recruiting		2024–2028
	NCT06346197	Phase 3	Not yet recruiting		2024–2028
	NCT06076837	Phase 1	Recruiting		2023–2025
	NCT06411691	Phase 1	Recruiting		2024–2027
	NCT04028063	Phase 2	Recruiting		2020–2025
	NCT06251973	Phase 2	Recruiting		2024–2027
	NCT06575725	Phase 2	Withdrawn		2024–2027
	NCT05845450	Phase 2	Recruiting		2023–2028
	NCT05672316	Phase 1/2	Active, not recruiting		2023–2025
	NCT05864534	Phase 2	Recruiting		2024–2025
	NCT06336902	Phase 1	Not yet recruiting		2024–2027
	NCT05627635	Phase 1/2	Recruiting		2023–2025
Zalifrelimab	NCT05572970	N/A	N/A	Zalifrelimab is a monoclonal antibody that targets CTLA-4, a protein on T cells that inhibits their activity. By binding to CTLA-4, zalifrelimab blocks this inhibitory signal, allowing T cells to remain active and attack cancer cells.	N/A
	NCT05033132	Phase 2	Withdrawn		2021–2025
	NCT04943848	Phase 1	Recruiting		2022–2025
	NCT04028063	Phase 2	Recruiting		2020–2026
	NCT04827953	Phase 1/2	Active, not recruiting		2021–2024
	NCT05375903	Phase 1	Recruiting		2022–2025
XmAb22841	NCT05695898	Phase 1	Active, not recruiting	XmAb22841 is a bispecific antibody that targets two immune checkpoints, CTLA-4 and LAG-3, simultaneously. By blocking both checkpoints, XmAb22841 aims to enhance T cell activation and anti-tumor immunity more effectively than blocking either checkpoint alone.	2023–2025
XTX101	NCT04896697	Phase 1/2	Recruiting	Vilastobart (XTX101) is a tumor-activated anti-CTLA-4 antibody. Once activated, it blocks CTLA-4, enhancing the anti-tumor immune response while aiming to minimize systemic side effects often associated with traditional CTLA-4 blockade.	2021–2026

CTLA-4 is an inhibitory receptor on T cells that competes with CD28 for B7 binding on APCs, sending signals that reduce T cell activation and proliferation.^374,375^ This checkpoint is crucial during early T cell activation in lymphoid organs, helping to balance the immune response and prevent overactivation that could lead to autoimmunity. While CTLA-4 maintains immune homeostasis, tumors often exploit this pathway to evade immune detection. Several CTLA-4 inhibitors have shown promising potential in enhancing T cell immune responses and improving treatment outcomes in cancer therapy. These inhibitors work by blocking CTLA-4, thus boosting the immune system’s ability to target and destroy tumor cells. Clinical trials have demonstrated the safety and tolerability of these agents when used alone or in combination with PD-1/PD-L1 inhibitors.^376^ CTLA-4 inhibitors showed enhanced anti-tumor activity in various cancers, including NSCLC,^377^ and HCC.^378^ Ongoing studies, such as those evaluating AGEN1884, BMS-986218, and quavonlimab, continue to explore optimal dosing strategies, potential combinations, and long-term efficacy, aiming to further expand the therapeutic benefits of immune checkpoint inhibition in cancer treatment.

Several ICIs have been approved by the FDA (Table 5). The U.S. FDA first approved ipilimumab as an immune checkpoint inhibitor for treating advanced melanoma.^379^ It is a fully human monoclonal antibody that binds to CTLA-4 and inhibits its interaction with CD80/CD86. Hence, increasing T cell activation and anti-tumor immune responses.^151,380^ By inhibiting CTLA-4, ipilimumab restores the immune system’s ability to recognize and attack cancer cells, counteracting the tumor’s immune evasion strategy of suppressing T cell activity. This correlation between the immune evasion mechanism and the therapeutic effect of ipilimumab highlights how targeting CTLA-4 can help overcome immune suppression and enhance anti-tumor immunity.^381^ Investigations have shown that ipilimumab can provide long-term responses and increase survival in melanoma patients. However, because of its widespread immune system activation, it has been linked to irAEs.

**Table 5:** Overview of approved immune checkpoint inhibitors in cancer therapy

Inhibitor	Target	Mechanism of Action	Year
Pembrolizumab	PD-1	Blocks PD-1 receptor on T cells, restoring their ability to detect and attack cancer cells.	2014
Nivolumab	PD-1	Binds to PD-1 on T cells, preventing it from engaging with PD-L1, thus enhancing the immune response against the tumor.	2014
Atezolizumab	PD-1	Inhibits PD-L1 on tumor cells, preventing T cell deactivation and promoting immune surveillance.	2016
Cemiplimab	PD-1	Inhibits PD-1 receptor on T cells, allowing them to attack tumor cells effectively.	2018
Camrelizumab	PD-1	Blocks PD-1 on T cells to promote immune-mediated tumor destruction.	2021
Avelumab	PD-L1	Binds PD-L1 on cancer cells, blocking it from deactivating T cells and enhancing immune recognition.	2017
Durvalumab	PD-L1	Prevents PD-L1 on cancer cells from binding to PD-1 on T cells, thereby supporting immune-mediated cell death.	2017
Ipilimumab	CTLA-4	Targets CTLA-4 on T cells, promoting T cell activation and infiltration into tumor tissue.	2011
Tremelimumab	CTLA-4	Blocks CTLA-4 to enhance T cell activity and immune system response against cancer cells.	2022
Relatlimab	LAG-3	Targets LAG-3 on T cells, allowing increased T cell function and reducing immune exhaustion.	2022

Tremelimumab is a monoclonal antibody approved for HCC and NSCLC, used with durvalumab and chemotherapy for unresectable cases.^382^ Tremelimumab is indicated as a first-line treatment for metastatic NSCLC in patients who do not have sensitizing EGFR mutations or ALK-positive mutations. It works by binding to and inhibiting CTLA-4, a protein that modulates T cell activity, thereby preventing the interaction of the B7.1 and B7.2 ligands with CTLA-4. This inhibition enhances T cell activation, promoting tumor destruction and reducing tumor growth. The FDA-approved tremelimumab for unresectable HCC in October 2022 and for metastatic NSCLC in November 2022.^382^ Additionally, it received a positive opinion from the EMA CHMP for advanced hepatocellular carcinoma in December 2022. Initially developed by Pfizer, tremelimumab is now being investigated by AstraZeneca for various cancers.

The PD-1/PD-L1 pathway plays an important role in maintaining immunological tolerance and avoiding autoimmunity by suppressing T cell activation in peripheral tissues, particularly within the TME.^383^ When PD-1 attaches to PD-L1, it sends an inhibitory signal that lowers T cell proliferation, cytokine generation, and cytotoxic activity, effectively suppressing the anti-tumor immune response.^383^ Tumors exploit the PD-1/PD-L1 pathway to evade immune surveillance by upregulating PD-L1 expression on tumor cells.^384,385^ This immune evasion strategy enables tumors to escape detection and spread without interference from the immune system. Blocking the PD-1/PD-L1 interaction with monoclonal antibodies restores T cell function, enhancing the immune system’s ability to detect and destroy cancer cells.^386,387^ The primary action of PD-1/PD-L1 inhibitors involves blocking the interaction between PD-1 on T cells and PD-L1 on tumor cells, which enhances T cell activation and promotes anti-tumor responses. Clinical studies have shown that these inhibitors not only provide a survival advantage over traditional therapies but also lead to long-lasting remissions even after other treatments have failed.^388^

The introduction of monoclonal antibodies that block PD-1 or PD-L1 has transformed cancer therapy. Pembrolizumab,^389,390^ and nivolumab,^391^ are widely used PD-1 inhibitors, while atezolizumab, durvalumab, and avelumab specifically target PD-L1.^57,392^ These ICIs are approved for treating various cancers. The success of PD-1/PD-L1 inhibitors in clinical settings highlights their ability to counteract the immune evasion mechanism of tumor cells that exploit PD-1/PD-L1 signaling. Combining these inhibitors with CTLA-4 inhibitors like ipilimumab, or with other therapies, enhances immune responses by targeting multiple immune evasion mechanisms. This multi-pronged approach helps overcome cancer’s ability to escape immune detection through redundancy in immune evasion pathways.^393,394^

PD-1/PD-L1 inhibitors have presented durable responses and significant survival benefits in advanced cancer patients by reinvigorating exhausted T cells, leading to long-lasting remissions even after other treatments fail. Their success has spurred the development of combination therapies, such as pairing PD-1 inhibitors with CTLA-4 inhibitors, which have demonstrated superior efficacy in cancers like melanoma but also carry a higher risk of immune-related side effects.^388,395^ Other strategies involve combining ICIs with VEGF inhibitors, chemotherapy, radiation, and adoptive T cell therapies to boost anti-tumor responses. However, resistance remains a challenge, leading to ongoing research into biomarkers like PD-L1 expression, tumor mutational burden, and immune cell populations to predict responses and address resistance mechanisms. For instance, patients with high PD-L1 expression or microsatellite instability (MSI) have demonstrated greater responses to these therapies.^388,395^

### Cancer vaccines

Cancer vaccines are therapeutic strategies that activate the immune system to recognize and destroy existing tumors by targeting tumor-specific antigens. There are various types of cancer vaccines, including preventive, therapeutic, peptide-based, DNA, cell-based, mRNA, oncolytic virus, neoantigen, whole-cell, and heat shock protein vaccines (Table 6). These vaccines can be composed of various elements, including TAAs, whole tumor cells, or DCs, all designed to initiate a robust immune response specifically targeting cancer cells.

**Table 6:** Overview of cancer vaccine types, mechanisms, and targets

Vaccine Type	Examples	Mechanism	Cancer target
HPV vaccine	Gardasil, Cervarix	It is used as a preventive vaccine. It targets HPV strains 16 and 18, which are associated with cervical, anal, and oropharyngeal cancers. Stimulates the immune system to attack HPV-infected cells.	Cervical cancer, anal cancer, throat cancer
Hepatitis B vaccine	Hepatitis B vaccine	It is used as a preventive vaccine. It prevents HBV infection that can lead to chronic hepatitis and hepatocellular carcinoma. Generates immune memory to clear HBV upon exposure.	Liver cancer
Sipuleucel-T (Provenge)	Personalized vaccine	Uses patient’s own dendritic cells exposed to PAP (prostatic acid phosphatase) to boost the immune response against cancer cells.	Prostate cancer
MAGE-A3	MAGE-A3 peptide vaccine	Targets melanoma-associated antigen 3 (MAGE-A3) expressed in some cancers, aiming to stimulate T cell response to cancer cells displaying this antigen.	Melanoma, non-small cell lung cancer
NY-ESO-1	NY-ESO-1 peptide vaccine	Targets NY-ESO-1, a cancer/testis antigen highly expressed in various cancers, to boost CD4+ and CD8+ T cell responses.	Melanoma, ovarian cancer, Myxoid/round cell liposarcoma, Neuroblastoma, Synovial sarcoma,
DNA Vaccines	INO-5401	DNA vaccine encoding tumor antigens to induce T cell responses targeting cancer cells.	Glioblastoma, prostate cancer
VGX-3100	E6 and E7 genes of HPV	A DNA vaccine against HPV, aiming to treat high-grade cervical dysplasia by inducing cellular immune responses.	Cervical cancer
GVAX	Tumor cells genetically modified to secrete GM-CSF	Cancer cells are irradiated and modified to release GM-CSF, an immune stimulant, to attract immune cells to attack the cancer cells.	Pancreatic cancer, prostate cancer
Dendritic Cell Vaccines	DCVax-L	Patient’s dendritic cells are loaded with tumor lysate to stimulate T cell responses targeting tumor cells.	Glioblastoma
mRNA Vaccines	mRNA-4157	Uses mRNA encoding neoantigens to stimulate an immune response against tumor-specific mutations.	Melanoma
BNT122	mRNA vaccine with personalized neoantigens	Personalized vaccine using mRNA encoding unique neoantigens to boost specific immune response against tumor cells.	Solid tumors, melanoma
T-VEC (Talimogene laherparepvec)	Modified herpes simplex virus type 1	Engineered virus selectively infects and kills cancer cells while releasing GM-CSF to stimulate an anti-tumor immune response.	Melanoma
Reolysin	Reovirus-based therapy	Exploits cancer cells’ activated Ras pathway to selectively replicate and kill tumor cells, triggering an immune response.	Head and neck cancer, pancreatic cancer
Neoantigen Vaccines	Personalized vaccines	Created by sequencing the patient’s tumor to identify specific neoantigens unique to the tumor, then inducing an immune response against those antigens.	Various cancers
Whole-cell Vaccines	Canvaxin	Uses irradiated whole tumor cells to induce an immune response; often combined with adjuvants to enhance immunogenicity.	Melanoma
Heat Shock Protein (HSP) vaccines	HSPPC-96	Isolated from the patient’s tumor cells, these proteins are linked to tumor antigens, aiming to improve immune recognition and response.	Glioblastoma

The main mechanism of cancer vaccines involves presenting tumor antigens to the immune system. These antigens, which can be proteins, peptides, or other molecules, are either unique to cancer cells or overexpressed in them. Upon administration, cancer vaccines introduce these antigens to APCs, which process and display them on their surface with major MHC molecules.^396^ This antigen presentation is crucial for activating CTLs, which then seek out and destroy tumor cells expressing the same antigens.^396,397^ In addition to activating CTLs, cancer vaccines also stimulate helper T cells and B cells, resulting in a comprehensive immune response that involves both humoral and cellular immunity.^398^ By inducing a memory response, cancer vaccines aim to provide long-term protection against tumor recurrence.

Cancer vaccines are classified into various forms based on their composition and function. Peptide-based vaccines consist of short peptides derived from TAAs, which are presented by MHC molecules on the surface of APCs to activate T cells. While peptide-based vaccines are highly specific and can be tailored to target mutations or overexpressed proteins in cancer cells, their efficacy can be limited by MHC diversity and potential tumor antigen escape.^396,399^ DC vaccines involve extracting dendritic cells from the patient, loading them with tumor antigens in vitro, and reinfusing them. This method harnesses dendritic cells’ ability to activate T cells against cancer. Sipuleucel-T (Provenge), an FDA-approved DC vaccine for metastatic castration-resistant prostate cancer, exemplifies the potential of this approach, despite its complexity and cost.^400^ Whole-cell vaccines use either autologous or allogeneic tumor cells to stimulate an immune response, presenting a broad range of antigens. However, they may induce non-specific immune responses and often require adjuvants to enhance efficacy.^401,402^ Viral vector-based vaccines employ modified viruses to deliver tumor antigens to cells, eliciting strong immune responses, although their use is restricted by pre-existing immunity to the viral vector and potential safety concerns.^403^ The diversity of cancer vaccines addresses several challenges, such as tumor antigen heterogeneity and immune tolerance. Tumor antigen heterogeneity, a key issue in vaccine efficacy, arises when tumor cells present different antigens, leading to tumor antigen escape or tumor subclones that evade immune detection due to variations in antigen expression. Furthermore, the different vaccine types aim to overcome immune tolerance, where the immune system fails to adequately respond to tumor antigens, recognizing them as self-antigens or failing to distinguish them from normal cells, thus enhancing the overall immune response against tumors.^404,405^

The clinical success of cancer vaccines has been mixed, with some, like Sipuleucel-T for metastatic prostate cancer, improving survival, while others show limited efficacy due to challenges such as tumor-induced immunosuppression, antigen loss, and immune tolerance. Tumor heterogeneity and the resemblance of tumor antigens to self-proteins hinder robust immune responses. To address these issues, researchers are exploring adjuvants, novel tumor antigens, personalized vaccines, and combinations with immunotherapies like checkpoint inhibitors or CAR-T cell therapy. Neoantigen vaccines, which target tumor-specific mutations, offer a promising, highly personalized approach. The limited success of some cancer vaccines can be attributed to tumor-induced immunosuppression within the TME. Tumors can inhibit immune cells through the secretion of immunosuppressive factors or by inducing the expression of immune checkpoint proteins. Combining cancer vaccines with checkpoint inhibitors holds promise for overcoming this barrier by preventing immune responses from being turned off.^406,407^ Additionally, neoantigen vaccines offer a strategy to counteract immune tolerance and tumor heterogeneity. These vaccines are designed to target mutations specific to individual tumors, providing a highly personalized approach that may more effectively address antigen loss and immune evasion, without affecting normal tissues, thus improving vaccine efficacy.^407^

Overall, cancer vaccines represent a powerful and versatile approach in cancer treatment, capable of stimulating strong and long-lasting immune responses against tumor cells. Although challenges are posed by immunosuppression, antigen heterogeneity, and immune tolerance.^408^ Ongoing research and innovation are driving the development of increasingly effective and tailored vaccine approaches. As our understanding of tumor biology and immune processes deepens, cancer vaccines will play an increasingly essential role in overcoming immune evasion and enhancing patient outcomes in cancer therapy.

### Oncolytic viruses

Oncolytic viruses (OVs) are a new and promising method to cancer treatment that takes advantage of viruses’ inherent capacity to specifically target and kill tumor cells while activating the host’s immune response. Unlike conventional therapies that primarily focus on directly killing cancer cells, OVs employ a dual mechanism of action: oncolysis and immune modulation. This combination not only helps in reducing the tumor burden but also addresses one of the most challenging aspects of cancer therapy—immune evasion by the tumor.^409^ OVs counteract tumor manipulation of immune checkpoints, such as PD-L1,^410,411^ and CTLA-4,^412,413^ which tumors use to evade immune surveillance. By infecting tumor cells and modulating the TME, OVs can lower the expression of these immune checkpoints, thereby making tumor cells more susceptible to immune attack and improving the effectiveness of the immune response against cancer.^414,415^

OVs selectively infect and proliferate within tumor cells, resulting in their death and the release of TAAs, which initiate a cascade of immunological responses.^416,417^ These antigens are taken up by APCs, which activate T cells against the tumor, generating a systemic anti-tumor immune response that targets both primary and metastatic lesions. Tumors often evade immune detection by altering antigen presentation and suppressing T cell activation. Oncolytic viruses address this challenge by enhancing antigen presentation. The virus-induced lysis of tumor cells releases TAAs, which are then presented by APCs to T cells, promoting a sustained immune response.^418,419^ This process helps counteract immune tolerance, a mechanism that tumors exploit to avoid immune detection, by stimulating the immune system to recognize and target tumor-specific antigens. A key aspect of immune evasion in cancer involves the manipulation of immune checkpoints, recruitment of immunosuppressive cells, and alteration of antigen presentation, all of which OVs counteract effectively. OVs lower the expression of immune checkpoint molecules in the TME, making tumor cells more susceptible to immunological attack. Additionally, by promoting a pro-inflammatory environment, OVs enhance the infiltration and activation of effector T cells, which are crucial for a strong anti-tumor response.^416^ OVs also reverse the immunosuppressive nature of the TME by inducing the release of cytokines, shifting the TME from immune-suppressive to immune-activating. This reprogramming prevents tumors from escaping immune surveillance.^416,417^ Furthermore, OVs enhance antigen presentation by lysing tumor cells and releasing TAAs, providing APCs with a rich source of antigens, thereby ensuring robust and sustained T cell activation essential for long-term tumor control.^416,420^ Tumor cells often evolve to escape detection by the immune system through antigen loss or antigen variation.^421,422^ Oncolytic viruses help overcome this by inducing antigen release upon tumor cell lysis, providing APCs with a broader range of tumor-associated antigens, thus improving the immune response’s ability to detect and attack the tumor.^423,424^

The therapeutic application of oncolytic viruses has shown great promise, with several OVs currently undergoing studies. Talimogene laherparepvec (T-VEC), an engineered herpes simplex virus type 1 (HSV-1), is a remarkable example of an approved treatment for metastatic melanoma.^425,426^ T-VEC has been modified to express GM-CSF, which improves the anti-tumor-immune response by increasing dendritic cell recruitment and activation. Clinical studies have demonstrated that T-VEC not only reduces the size of injected tumors but also induces systemic immune responses capable of targeting distant metastases, highlighting the potential of OVs to treat metastatic disease.^425^

Despite their promising potential, several challenges remain in the clinical application of oncolytic viruses. A major challenge is delivering OVs to tumor sites, especially in metastatic cases, and the risk of the patient’s immune system neutralizing the virus before it can act. Ongoing research aims to develop combination therapies incorporating immune checkpoint inhibitors, adoptive cell therapies, and other immunomodulatory drugs to overcome these obstacles. These initiatives seek to improve the overall efficacy of OVs and optimize their therapeutic potential.

### Adoptive T cell therapy

Adoptive T cell therapy (ACT) is an advanced cancer immunotherapy that enhances the immune system’s ability to target and eliminate tumor cells by isolating, modifying, and expanding a patient’s or donor’s T cells before reintroducing them into the body. This approach directly combats immune evasion mechanisms, such as immunosuppressive TME and reduced antigen presentation, by supplying a large number of tumor-specific, activated T cells. These engineered T cells are primed to recognize specific cancer antigens, leading to a more focused and effective immune response against the tumor.^427,428^ TME is often immunosuppressive, impeding immune cell function by secreting cytokines and recruiting immunosuppressive cells.^429,430^ ACT helps overcome this challenge by reintroducing activated T cells that are engineered to target cancer cells, which can function in a more immune-activated state and counteract the immunosuppressive signals present in the TME.^431,432^ Additionally, tumor cells often evade immune detection by altering antigen presentation, such as reducing the expression of MHC molecules or evading immune surveillance mechanisms.^433,434^ ACT addresses this by introducing tumor-specific T cells that have already been primed to recognize and respond to these specific tumor-associated antigens, bypassing the need for enhanced antigen presentation and directly targeting the tumor cells.

There are several forms of adoptive T cell therapy, each with its unique advantages and applications. One of the most well-known approaches is CAR-T cell therapy.^435,436^ The therapy has demonstrated noteworthy success in treating hematological malignancies such as acute lymphoblastic leukemia (ALL) and some forms of lymphomas, resulting in full remissions in patients who have not responded to conventional treatments.^436^ Tumor heterogeneity and antigen escape, a major challenge in cancer immunotherapy, where tumors can lose or alter the expression of targeted antigens to evade immune detection. CAR-T cell therapy addresses this by engineering T cells to specifically target tumor antigens that are overexpressed in tumor cells. This approach can overcome antigen escape by focusing on tumor markers that are consistently present across a wide range of tumor cells, ensuring a more uniform and persistent immune response against the cancer, even when individual tumor cells may lose or alter specific antigens. This helps improve the efficacy of treatment and mitigates the challenges posed by tumor heterogeneity. CAR-T therapy is a novel cancer treatment that genetically modifies T cells to express chimeric antigen receptors, enabling them to identify and eliminate cancer cells, particularly in blood cancers.^437,438^ CARs combine antibody targeting with T cell destruction, consisting of an antigen-binding domain, transmembrane domain, and signaling domains that activate T cells to eliminate tumor cells.^439,440^

**Fig. 7:**
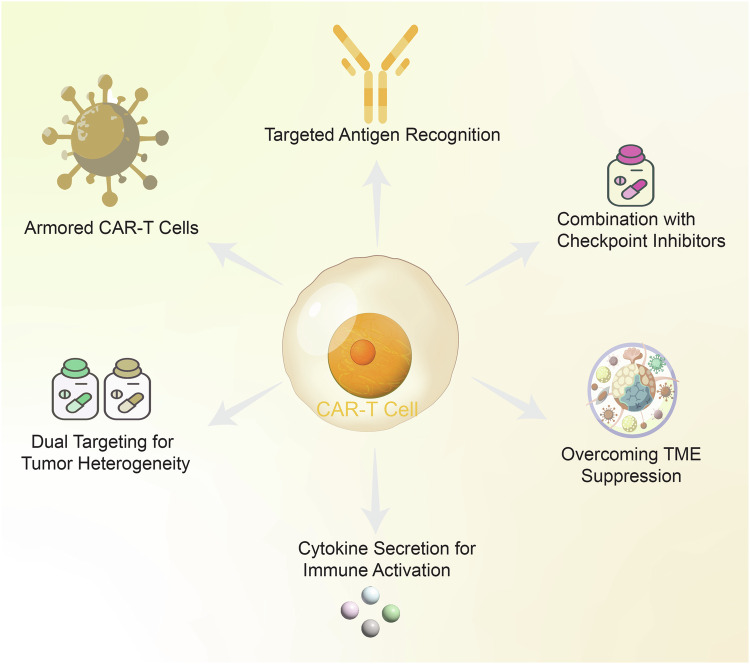
Strategies employed by CAR-T cells to counter immune evasion in cancer. This figure illustrates the multifaceted strategies employed by CAR-T cells to overcome immune evasion in cancer. CAR-T cells are engineered for targeted antigen recognition, allowing precise targeting of tumor-specific antigens, thereby minimizing off-target effects. Checkpoint inhibition is achieved by combining CAR-T cells with agents that block immune checkpoints, effectively countering inhibitory signals from the tumor. To resist the TME immunosuppressive influences, CAR-T cells are designed to withstand suppressive factors. Cytokine secretion by CAR-T cells amplifies the immune response by recruiting and activating other immune cells within the tumor vicinity. Additionally, dual-targeting CAR-T cells address tumor heterogeneity by recognizing multiple antigens, thereby reducing the risk of immune evasion through antigen loss. Lastly, armored CAR-T cells are engineered to express additional receptors, enhancing resilience against the hostile TME and supporting sustained immune activity

There are various benefits of the CAR-T cell treatment approach (Fig. 7). One of the primary benefits is that CARs are intended to identify antigens independently of MHC molecules, which are frequently downregulated in tumor cells as a mechanism of immune evasion.^441^ This advancement allows CAR-T cells to specifically target tumor cells that might evade recognition by conventional T cells. The most notable success of CAR-T cell therapy thus far has been in treating B cell malignancies. CARs designed to target the CD19 antigen—a protein commonly found on B cells—have demonstrated remarkable clinical efficacy.^442^

Tisagenlecleucel (Kymriah) and axicabtagene ciloleucel (Yescarta) were the first CAR-T cell therapies approved by the U.S. FDA, both designed to target CD19 in treating specific leukemia and lymphoma types. These therapies have shown remarkable success in patients with relapsed or treatment-resistant B cell cancers, leading to high complete remission rates in cases unresponsive to conventional treatments.^443,444^ The success of CAR-T cell therapy targeting CD19 has spurred the exploration of CAR-T therapies aimed at other antigens and various cancer types. For instance, CAR-T cell treatments targeting CD19 have received approval for use in relapsed and/or refractory B cell lymphoma as well as B cell acute lymphoblastic leukemia.^445^ Ongoing research aims to develop CAR-T cell therapies targeting additional antigens, including BCMA, which has been approved for treating multiple myeloma.

However, despite the success of CAR-T cell therapy, several challenges remain. One of the most significant issues is the development of antigen escape, where tumor cells downregulate or lose expression of the targeted antigen, rendering CAR-T cells ineffective.^446,447^ This phenomenon highlights the need for multi-targeted CARs or combination therapies that can address tumor heterogeneity and prevent relapse.

Another challenge is managing treatment-related toxicities associated with CAR-T cell therapy, with cytokine release syndrome (CRS) and neurotoxicity being the most significant. CRS is a systemic inflammatory reaction caused by the rapid activation and proliferation of CAR-T cells, leading to the release of high levels of cytokines. While CRS can pose serious risks, it is typically manageable with supportive care and immunosuppressive medications like tocilizumab, an IL-6 receptor antagonist. Neurotoxicity, often referred to as CAR-T cell-related encephalopathy syndrome (CRES), can range from mild confusion to severe neurological dysfunction.^448,449^ The mechanisms underlying neurotoxicity are not fully understood, and ongoing research is focused on developing strategies to mitigate these risks.

While CAR-T cell treatment has been highly successful in hematologic cancers, it has proven more difficult to apply to solid tumors. Solid tumors present multiple difficulties that restrict the efficiency of CAR-T cells, including the immunosuppressive TME, physical barriers that inhibit T cell penetration, and the absence of highly specific TAAs that are uniformly expressed throughout tumor cells.^450^

A dense ECM, hypoxia, and the presence of immunosuppressive cells such as Tregs, MDSCs, and TAMs characterize the TME of solid tumors.^450^ These factors collectively hinder the ability of CAR-T cells to penetrate the tumor, remain functional, and exert their cytotoxic effects. Additionally, many of the antigens expressed by solid tumors are also found on normal tissues, raising the risk of on-target, off-tumor toxicity.^450,451^

To tackle these challenges, researchers are exploring various strategies to enhance the efficacy of CAR-T cells against solid tumors. These include creating CAR-T cells that produce pro-inflammatory cytokines or chemokines to modify the TME, using CARs that target multiple antigens to decrease antigen escape, and designing CAR-T cells with improved migratory and persistence characteristics. Furthermore, combination therapies combining CAR-T cells with checkpoint inhibitors, oncolytic viruses, or other immunotherapies are being studied to synergize anti-tumor responses.^451,452^

The future of CAR-T cell therapy focuses on refining its design to treat a broader range of cancers, including the development of “universal” CAR-T cells that bypass patient-specific MHC dependence, enabling off-the-shelf therapies for faster deployment. Advances like CRISPR-Cas9 gene editing are enhancing safety, reducing immunogenicity, and improving CAR-T cell functionality. Combining CAR-T cells with therapies like immune checkpoint inhibitors shows promise in overcoming resistance and boosting anti-tumor responses. Additionally, the incorporation of safety switches allows controlled elimination of CAR-T cells to mitigate severe toxicity, enhancing the safety of this treatment.

Another form of ACT is TIL therapy. TILs are T cells that have naturally infiltrated the tumor but are often present in insufficient numbers or are functionally impaired due to the immunosuppressive TME. TIL therapy entails extracting T cells from a patient’s tumor, expanding them outside the body to enhance their quantity and functionality, and then reinfusing them into the patient. This approach has demonstrated significant potential, especially in the treatment of solid tumors, where it can lead to sustained responses in individuals with advanced disease. Tumors are capable of creating an immune-tolerant environment, where infiltrating immune cells, including T cells, are either inactivated or suppressed by the TME.^453,454^ TIL therapy addresses this by expanding and activating the T cells outside the body, thereby bypassing the immune suppression occurring at the tumor site. This process reintroduces functionally enhanced T cells that are better equipped to overcome tumor-induced immune tolerance, allowing them to resume their anti-tumor functions and effectively target and destroy tumor cells.^455^

T Cell Receptor (TCR) therapy is a form of ACT where T cells are genetically modified to express TCRs that recognize specific peptide-MHC complexes on tumor cells. This approach allows targeting of intracellular antigens presented on the cell surface by MHC molecules, broadening the range of cancer antigens addressed compared to CAR-T cell therapies. TCR treatment has shown effectiveness in treating various malignancies.^456,457^ Tumors evade immune recognition by downregulating MHC molecules, impairing tumor-antigen presentation to immune cells. TCR therapy counters this by genetically modifying T cells to express specific TCRs that recognize peptide-MHC complexes, allowing them to target and respond to intracellular antigens even when presented by MHC molecules.^458,459^ This approach helps bypass the tumor’s evasion of immune detection by enhancing the recognition of altered or diminished antigen presentation on tumor cells, making the immune response more effective.

Despite the success of adoptive T cell therapy, several obstacles remain, including identifying appropriate target antigens specific to cancer cells while avoiding off-target effects and potential damage in normal tissues. The TME challenges the adoptively transferred T cells due to immunosuppressive factors. Researchers are exploring combination therapies and gene editing, like CRISPR/Cas9, to enhance T cell efficacy and accessibility, aiming for broader patient availability.

### Epigenetic modulation

Epigenetic modulation has emerged as an intriguing method in cancer treatment, particularly for overcoming immune evasion. Epigenetic alterations play a crucial role in regulating gene expression without altering the underlying DNA sequence. In cancer, these modifications are often co-opted by tumor cells to silence tumor suppressor genes, activate oncogenes, and alter the immune landscape of the TME.^460^ By targeting these epigenetic alterations, it is possible to reprogram cancer cells and the surrounding TME to enhance immune recognition and response, offering a promising approach to combat immune evasion.^461^ Tumor cells often evade immune recognition by epigenetically silencing the expression of antigen presentation machinery through DNA methylation and histone modifications.^462,463^ This silencing prevents the presentation of tumor antigens to immune cells, enabling the tumor to escape immune surveillance. Targeting these epigenetic changes can restore the expression of antigen presentation machinery, improving immune recognition and response, and enhancing the overall efficacy of the immune system in targeting the tumor.^461,464^

Beyond antigen presentation, epigenetic alterations also regulate immune checkpoint molecules, which tumors exploit to suppress T cell activity.^198,465^ Targeting these epigenetic changes can modulate immune checkpoint pathways, and studies show that epigenetic drugs can alter PD-L1 expression, suggesting potential synergy when combined with immune checkpoint inhibitors.^198^ Additionally, epigenetic modulation influences TME, often characterized by immunosuppression through Tregs, MDSCs, and cytokines. Epigenetic drugs, such as EZH2 inhibitors, can reduce Treg suppressive activity and reprogram the TME to support anti-tumor immunity.^465^

The therapeutic application of epigenetic regulation in cancer therapy is fast progressing, with several therapies authorized and many more in clinical studies. To enhance effectiveness, these medications are often combined with other treatment methods like chemotherapy, radiation, and immunotherapy. For instance, pairing epigenetic drugs with immune checkpoint inhibitors has shown promise in various cancers, including NSCLC and melanoma. These combinations seek to overcome resistance to single-agent therapy and offer a more comprehensive approach to immune evasion.

## Emerging approaches in targeting immune evasion

### Bispecific antibodies

Bispecific antibodies represent an innovative category of therapeutic agents that provide a promising approach to overcoming immune evasion in cancer. Unlike conventional monoclonal antibodies, bispecific antibodies are designed to simultaneously recognize and bind to two different antigens (Table 7). This dual targeting ability enables bispecific antibodies to connect cancer cells with immune cells, thereby enhancing the immune response against tumors that have developed strategies to escape immune detection.^466^

**Table 7:** Bispecific antibodies, their targets, indications, and mechanisms of action

Bispecific Antibody	Targets	Indication	Mechanism
Blinatumomab (Blincyto)	CD19 and CD3	Acute lymphoblastic leukemia (ALL)	Directs T cells to CD19-expressing cancer cells, leading to T cell-mediated cytotoxicity^620^.
Emicizumab (Hemlibra)	Factor IXa and Factor X	Hemophilia A	Mimics missing activated Factor VIII activity in hemophilia A patients^621,622^.
REGN1979	CD20 and CD3	Non-Hodgkin’s lymphoma (NHL)	Engages T cells to kill CD20-positive B cells^623,624^.
MGD011	CD19 and CD3	B cell malignancies	Induces T cell-mediated cytotoxicity against CD19-expressing B cells^625,626^.
Catumaxomab	EpCAM and CD3	EpCAM-positive cancers	Cross-links EpCAM on tumor cells with CD3 on T cells to direct immune response against the tumor^627^.
Glofitamab	CD20 and CD3	Lymphoma	Engages T cells to eliminate malignant CD20-positive B cells^628^.
BAY 2010112	PSMA and CD3	Prostate cancer	Directs T cells to prostate cancer cells expressing PSMA^629,630^.
AFM13	CD30 and CD16A	Hodgkin lymphoma, non-Hodgkin lymphoma	Engages NK cells to target and kill CD30-positive tumor cells^631,632^.
M7824	PD-L1 and TGF-β	Various cancers	M7824 simultaneously blocks PD-L1, a key immune checkpoint, and TGF-β, an immunosuppressive cytokine^633,634^.
Mosunetuzumab	CD20 and CD3	Relapsed or refractory follicular lymphoma	Engages T cells to eliminate malignant CD20-positive B cells^635,636^.
AFM11	CD19 and CD3	B cell malignancies	Redirects T cells to CD19-expressing B cells, inducing cytotoxicity^637,638^.
DuoBody-CD3xCD20	CD20 and CD3	Non-Hodgkin lymphoma	Engage T cells to eliminate CD20-positive malignant B cells^639,640^.

One primary mechanism of bispecific antibodies is their ability to redirect cytotoxic T cells to tumor cells. These antibodies have one arm that binds to a specific antigen on cancer cell surfaces, like CD19 or HER2, and another arm that binds to CD3, a component of the T cell receptor complex on T cells.^467,468^ Bispecific antibodies physically connect T cells to tumor cells, positioning immune cells closer to cancer cells and enhancing T cell activation and subsequent tumor cell lysis. This approach effectively counters immune evasion tactics used by tumors, such as downregulating MHC molecules or producing immunosuppressive substances that typically limit T cell function.^467,468^

Blinatumomab, one of the first bispecific T cell engager (BiTE) antibodies to receive clinical approval, demonstrates the therapeutic potential of this approach. It targets CD19 on B cell malignancies and CD3 on T cells, enabling direct T cell-mediated killing of cancer cells.^469^ Its success in treating ALL has sparked significant interest in developing similar bispecific antibodies for other types of cancer, including solid tumors. For example, a recent study showed that blinatumomab improves overall survival in relapsed or refractory B cell ALL, with a median survival of 7.7 months versus 4.0 months for chemotherapy, and a complete remission rate of 34% compared to 16%.^470^ Bispecific antibodies are versatile because they can be customized to target a wide range of tumor-associated antigens, making them adaptable to different cancer types and patient populations.

Beyond redirecting T cells, bispecific antibodies are also being developed to target other immune effector cells, such as NK cells and macrophages. These approaches try to use the immune system’s entire range of capabilities to battle cancer. For example, bispecific antibodies that interact with NK cells are designed to bind tumor antigens and CD16, an activating receptor on NK cells, boosting NK cell-mediated cytotoxicity.^471,472^ Similarly, bispecific antibodies can be engineered to modulate the TME by recruiting and activating macrophages to phagocytose tumor cells or by blocking immunosuppressive signals within the tumor niche.^473,474^

The discovery of bispecific antibodies has the potential to circumvent some of the limitations of traditional immunotherapies, such as immune checkpoint inhibitors. While immune checkpoint inhibitors have changed cancer treatment by allowing the immune system to fight tumors, not all patients react to these drugs. Bispecific antibodies can be employed in conjunction with checkpoint inhibitors to improve therapeutic efficacy or as stand-alone therapy when checkpoint inhibition is ineffective. By providing a direct means of engaging immune cells with cancer cells, bispecific antibodies can bypass some of the resistance mechanisms that limit the effectiveness of other immunotherapies. For example, the combination of bispecific antibodies with PD-1/PD-L1 inhibitors offers a promising strategy in cancer immunotherapy. Such as bispecific antibodies like M7824, which target both PD-L1 and TGF-β, have shown notable clinical effectiveness in NSCLC when administered alongside PD-1 inhibitors.^475,476^ Additionally, next-generation bispecific antibodies targeting PD-L1 in combination with other immune checkpoints have shown significant anti-tumor activity in preclinical and early clinical studies. These innovative therapies have the potential to enhance the efficacy of existing immune checkpoint blockade treatments.^466,476^

Bispecific antibodies are emerging as a cutting-edge approach in cancer immunotherapy, offering promising potential by bridging immune cells and tumor cells to enhance immune recognition and attack cancer. Despite challenges in their design, manufacturing, and pharmacokinetics, ongoing research is refining these agents to improve stability, half-life, and specificity. Advances in protein engineering are driving the development of more potent and versatile bispecific antibodies, which are being explored in clinical trials for various cancers, both as monotherapy and in combination with other treatments. As these innovations progress, bispecific antibodies are expected to play a crucial role in overcoming immune evasion and improving patient outcomes.

### Nanotechnology and drug delivery systems

Nanotechnology and improved drug delivery systems are quickly developing as transformative tools in cancer immunotherapy, providing novel solutions to overcome immune evasion. The ability of tumors to evade immune surveillance remains a significant barrier to effective cancer treatment, often leading to treatment resistance and disease progression. By leveraging the unique properties of nanoparticles and engineered delivery systems, researchers are developing targeted therapies that can more effectively modulate the immune system, improve the delivery of immunotherapeutic agents, and enhance the overall anti-tumor response.^477,478^

Nanoparticles, with their small size and customizable surface properties, have emerged as powerful tools for delivering therapeutic agents directly to tumor sites. This targeted strategy improves drug accumulation within the TME and greatly lowers off-target effects, making it particularly beneficial in immunotherapy, where precise modulation of the immune response is critical for therapeutic success.^479^ Targeted delivery of nanoparticles can occur through passive or active mechanisms. Passive targeting takes advantage of the improved permeability and retention effect, allowing nanoparticles to collect in tumors due to the leaky vasculature around them. This enhances the concentration of therapeutic medicines at the tumor site while limiting the impact on healthy tissues.^479^ Active targeting, on the other hand, entails altering nanoparticle surfaces with specific ligands, such as antibodies or peptides, allowing the nanoparticles to attach preferentially to receptors overexpressed on cancer cells. This approach further improves the precision of immunotherapeutic agent delivery, ensuring that agents like cytokines or checkpoint inhibitors are released exactly where they are needed. Furthermore, recent discoveries have given rise to smart nanoparticles that may respond to biological cues or external stimuli. This allows for the controlled release of therapeutic drugs in response to specific conditions within the TME.^480,481^

**Fig. 8:**
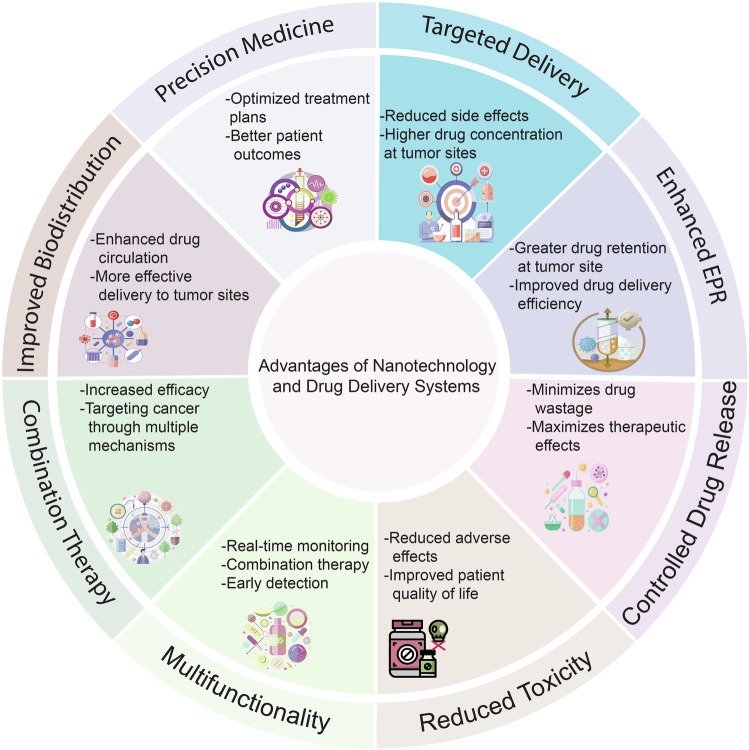
Advantages of nanotechnology and drug delivery systems in cancer treatment. This figure illustrates the advantages of nanotechnology and drug delivery systems in cancer treatment. Nanoparticles enhance targeted drug delivery by selectively binding to cancer cells, minimizing the exposure of healthy tissues, and reducing systemic toxicity. The enhanced permeability and retention (EPR) effect allows nanoparticles to accumulate at tumor sites due to leaky blood vessels, improving drug retention and therapeutic efficacy. Controlled drug release mechanisms ensure sustained and precise drug delivery, while multifunctionality enables simultaneous therapy and monitoring. Additionally, nanoparticles facilitate combination therapies, improve biodistribution by evading immune clearance, and allow for personalized treatment approaches, optimizing patient outcomes and minimizing side effects

Nanotechnology and drug delivery systems offer several advantages (Fig. 8). One of the primary advantages of using nanotechnology in cancer immunotherapy is the ability to co-deliver multiple agents simultaneously.^482^ For example, studies have shown that encapsulation of antigens and adjuvants in nanoparticles, such as E2 protein nanoparticles, can efficiently activate dendritic cells and T cells both in vitro and in vivo. The combined delivery of tumor-associated antigens with adjuvants like CpG ODN in nanoparticles has been demonstrated to induce strong CD8+ T cell proliferation and enhanced IFN-γ production, leading to prolonged overall survival in mouse tumor models.^483^ This co-delivery strategy can help overcome the immune tolerance often seen in tumors, where the immune system fails to recognize tumor-associated antigens as threats. By presenting these antigens in a more immunogenic context, nanoparticles can help break this tolerance and stimulate a robust anti-tumor immune response.

In addition to enhancing antigen presentation, nanotechnology also plays a crucial role in enhancing the efficacy of ICIs blockers in cancer therapy by addressing the limitations posed by resistance mechanisms within the TME. By enabling targeted delivery of ICIs, nanoparticles can increase the localized concentration of these drugs at tumor sites, thereby minimizing systemic toxicity and enhancing therapeutic effects.^484^ Functionalized with targeting ligands, these nanoparticles can precisely bind to receptors overexpressed on tumor or immune cells, overcoming barriers that hinder the effectiveness of ICIs. Moreover, the enhanced permeability and retention effect allows nanoparticles to accumulate preferentially in tumors, improving drug delivery,^484^ while stimuli-responsive systems release therapeutic agents in response to specific TME conditions, such as pH changes or enzymes, further optimizing treatment timing and precision.^484^ This nanoparticle-based approach can also help to circumvent resistance mechanisms by ensuring higher concentrations of ICIs reach the tumor, potentially improving outcomes for patients who have not responded to conventional ICI administration.^485^

Another promising application of nanotechnology in targeting immune evasion is the development of nanoscale vaccines. These nanovaccines are designed to mimic the size and structure of pathogens, improving their uptake by APCs and enhancing the subsequent activation of T cells. By delivering tumor antigens in this highly immunogenic format, nanovaccines can elicit strong and durable anti-tumor immune responses, making them an attractive option for cancer immunoprophylaxis and therapy.^486,487^ Additionally, nanovaccines can be tailored to include adjuvants or immune-stimulatory molecules, further boosting their immunogenicity and overcoming the immune suppression often seen in the TME.

Nanotechnology also plays a critical role in modulating the TME itself, which is essential for enhancing the effectiveness of cancer immunotherapy, as tumors often create an immunosuppressive TME that facilitates immune evasion. Nanoparticles can be engineered to deliver therapeutic agents that specifically target and reprogram this immunosuppressive environment. By targeting immunosuppressive cells, nanoparticles help restore the anti-tumor activity of effector immune cells, thereby boosting the efficacy of immunotherapy.^488,489^ Furthermore, nanoparticles can convert TAMs from an M2 to an M1 phenotype, boosting the anti-tumor immune response and altering the TME to support immune cell activity.^490^ Advanced nanoparticle systems, designed to respond to specific TME stimuli like pH changes, can also release their therapeutic payloads in the acidic environment of tumors, improving the targeted delivery of drugs and cytokines.^491,492^ This dual action of nanoparticles—targeted delivery and TME modulation—holds significant clinical potential in improving cancer treatment outcomes, making them a promising tool in the future of cancer immunotherapy.

Furthermore, researchers are exploring nanotechnology-based drug delivery systems to improve the pharmacokinetics and biodistribution of conventional cancer treatments, such as chemotherapy and radiotherapy, when combined with immunotherapy. These advanced delivery systems can target tumors more precisely, reduce off-target effects, and optimize the therapeutic potential of combined cancer treatments, enhancing the overall efficacy of cancer therapy. These systems can be engineered to release their therapeutic payloads in response to specific tumor stimuli, such as shifts in pH or enzymatic activity, ensuring that the active agents are triggered solely in the presence of cancer cells.^485^ This targeted approach not only improves treatment efficacy but also lowers the collateral damage to healthy tissues, which is a common limitation of traditional cancer therapies.

The use of nanotechnology in cancer immunotherapy offers a paradigm shift in our approach to treating immune-evasive malignancies. As research in this field continues to advance, the potential for nanotechnology to revolutionize cancer treatment becomes increasingly apparent. By enabling the precise delivery of immunotherapeutic agents, enhancing the immunogenicity of tumor antigens, and modulating the TME, nanotechnology offers a multifaceted approach to overcoming immune evasion and improving patient outcomes.

### Modulation of the tumor microenvironment

Modulation of the TME has emerged as a critical strategy in targeting immune evasion in cancer. The TME consists of a complex network of cancer cells, stromal cells, immune cells, and ECM components that collectively create a highly dynamic and immunosuppressive milieu. This milieu not only promotes tumor development and metastasis but also helps malignancies elude immune monitoring. By modifying the TME, researchers aim to reverse immune suppression, enhance immune cell infiltration, and restore the immune system’s ability to recognize and eliminate cancer cells.^6,493^

One of the most significant obstacles provided by the TME is the existence of various immunosuppressive cells, including Tregs, MDSCs, and TAMs. These cells release cytokines and other substances that suppress the activity of effector T cells and NK cells, which are critical for anti-tumor immunity.^494,495^ Emerging therapeutic approaches focus on selectively targeting these immunosuppressive populations to diminish their influence within the TME.^494,496^ For example, inhibitors of the CSF-1 receptor (CSF1R), which is expressed on TAMs, have shown promise in reducing the population of these cells, thereby alleviating their suppressive effects and reactivating anti-tumor-immune responses.^497^

Another key aspect of TME modulation is the normalization of the aberrant vasculature often found in tumors.^498,499^ Tumor blood vessels are typically disorganized, leaky, and poorly perfused, leading to hypoxic conditions that further promote immune suppression and resistance to therapy. Strategies aimed at normalizing the tumor vasculature can improve oxygenation and enhance the delivery of both immune cells and therapeutic agents to the tumor site. For instance, the use of angiogenesis inhibitors, such as bevacizumab, has been explored to normalize the vasculature and reduce hypoxia, thereby creating a more favorable environment for immune cell infiltration and activity.^498,500^

The ECM within the TME plays a crucial role in immune evasion. It can function as a physical barrier, preventing immune cells from penetrating the tumor core. Additionally, ECM components like collagen and hyaluronan can engage with immune cells to trigger immunosuppressive signaling pathways. Modulation of the ECM through enzymatic degradation or inhibition of its synthesis has been explored to enhance immune cell access to the tumor. For example, hyaluronidase enzymes can degrade hyaluronan, reducing the physical barrier and improving T cell infiltration into tumors.^493,501^

In addition to targeting cellular and structural components of the TME, metabolic modulation is gaining attention as a strategy to counteract immune evasion. The TME is often characterized by a unique metabolic profile that includes increased glycolysis, lactic acid accumulation, and depletion of essential nutrients such as glucose and amino acids.^502^ These metabolic conditions can impair the function of immune cells, particularly T cells, which rely on specific metabolic pathways for their activation and effector functions.^503,504^ Therapeutic approaches that modulate tumor metabolism, such as the use of glycolysis inhibitors or agents that modulate the tumor’s acidic environment, are being investigated to enhance the metabolic fitness of immune cells and improve their anti-tumor activity.

Furthermore, targeting the signaling pathways within the TME that contribute to immune suppression is another promising approach. For instance, the TGF-β signaling pathway is known to play a critical role in maintaining an immunosuppressive TME.^17^ Inhibitors of TGF-β signaling are being developed to block its effects, thereby reducing immune suppression and enhancing the efficacy of other immunotherapies.^505,506^ Similarly, targeting the Wnt/β-catenin signaling pathway, which is associated with the exclusion of immune cells from the TME, is being explored as a means to enhance immune infiltration and responsiveness.

Combination therapies that integrate TME modulation with existing immunotherapies, such as immune checkpoint inhibitors, are being actively explored to enhance the overall efficacy of cancer treatment. By reprogramming the TME to be more permissive to immune attack, these strategies aim to overcome resistance to therapies like PD-1/PD-L1 inhibitors, which have shown promise in preclinical models. Targeting the cellular, structural, metabolic, and signaling components of the TME can improve the effectiveness of immunotherapy and open new treatment avenues for cancers resistant to conventional approaches. As our understanding of the TME evolves, these therapies hold great potential to transform cancer treatment and improve patient outcomes.

### Immune agonists and modulators

Immune agonists and modulators are at the forefront of emerging approaches aimed at targeting immune evasion in cancer. These therapeutic agents are designed to enhance the activity of the immune system against tumor cells by either stimulating immune responses or modulating the immune environment to overcome the immunosuppressive tactics employed by tumors. By specifically activating immune cells or modifying immune pathways, immune agonists and modulators offer a novel and promising avenue for cancer treatment.^507^

Immune agonists typically function by binding to specific receptors on immune cells, thereby stimulating their activity. One of the key targets for immune agonists is the co-stimulatory receptors on T cells, such as OX40 (CD134), 4-1BB (CD137), and CD40. These receptors play a crucial role in T cell activation, proliferation, and survival. Agonistic antibodies targeting these receptors have been developed to enhance T cell responses against tumors.^508^ For example, OX40 agonists have indeed shown promise in enhancing responses to various cancer therapies, including chemotherapy and radiotherapy. OX40 (CD134) is a co-stimulatory receptor on T cells that, when activated, can enhance T cell proliferation, survival, and cytotoxic function. This leads to improved anti-tumor immunity.^91^ Preclinical studies have demonstrated that OX40 agonists can augment the efficacy of ICIs like anti-PD-1 and anti-CTLA-4.^509^ Additionally, OX40 agonists have been shown to enhance the effects of traditional cancer therapies such as chemotherapy and radiotherapy by promoting a more robust immune response against tumor cells.

Similarly, CD40 agonists activate APCs, enhancing tumor antigen presentation and T cell stimulation, leading to stronger anti-tumor immune responses.^510,511^ The interaction between CD40 and its ligand, CD40L, is crucial for the maturation of dendritic cells, enabling more effective antigen presentation and sustained T cell responses.^512^ Moreover, combining CD40 agonists with other therapies, such as chemotherapy or checkpoint inhibitors, has shown promise in improving therapeutic outcomes by maximizing immune activation and overcoming tumor evasion.^510,513^

In addition to immune agonists, modulators of the immune environment are being developed to counteract the immunosuppressive mechanisms within the TME.^514^ These modulators include agents that target cytokines, chemokines, and other signaling molecules that contribute to immune suppression. For example, IL-2 and IL-12 are cytokines with potent immune-stimulatory properties, and recombinant versions or analogs of these cytokines are being explored as cancer therapies.^515^ IL-2 has a long history of use in cancer immunotherapy, but its high toxicity has limited its application. Recent efforts have focused on engineering IL-2 variants with reduced toxicity and enhanced specificity for immune-activating receptors, thereby improving its therapeutic index.

Checkpoint modulators represent another category of immune modulators that are gaining traction in cancer therapy. While immune checkpoint inhibitors targeting PD-1/PD-L1 and CTLA-4 have revolutionized cancer treatment, resistance to these drugs poses a challenge. To combat this, novel checkpoint modulators targeting other inhibitory receptors are being developed.^516,517^ These modulators are designed to relieve the immune suppression imposed by these additional checkpoints, thereby reinvigorating exhausted T cells and enhancing their ability to attack tumors.

Furthermore, emerging approaches are exploring the use of small molecules and peptide-based modulators that can selectively alter immune signaling pathways. These modulators can fine-tune the immune response by either enhancing immune activation or reducing excessive inflammation that may cause autoimmunity. For instance, STING agonists are being studied for their potential to activate innate immune responses within the TME, resulting in type I interferon production and subsequent activation of adaptive immunity.^518,519^ These agonists have shown promise in preclinical models, particularly in combination with other immunotherapies, such as checkpoint inhibitors and vaccines.

The development of immune agonists and modulators represents a rapidly evolving field with significant potential to enhance the efficacy of cancer immunotherapy. By specifically targeting the immune system’s regulatory pathways, these agents offer a tailored approach to overcoming immune evasion, one of the major challenges in cancer treatment. As research continues to unravel the complexities of immune regulation in cancer, these emerging therapies hold great promise for improving outcomes in patients with difficult-to-treat tumors. The integration of immune agonists and modulators into the broader immunotherapy landscape is likely to play a key role in the next generation of cancer treatments, providing new hope for patients facing immune-resistant cancers.

## Challenges and future directions

**Fig. 9:**
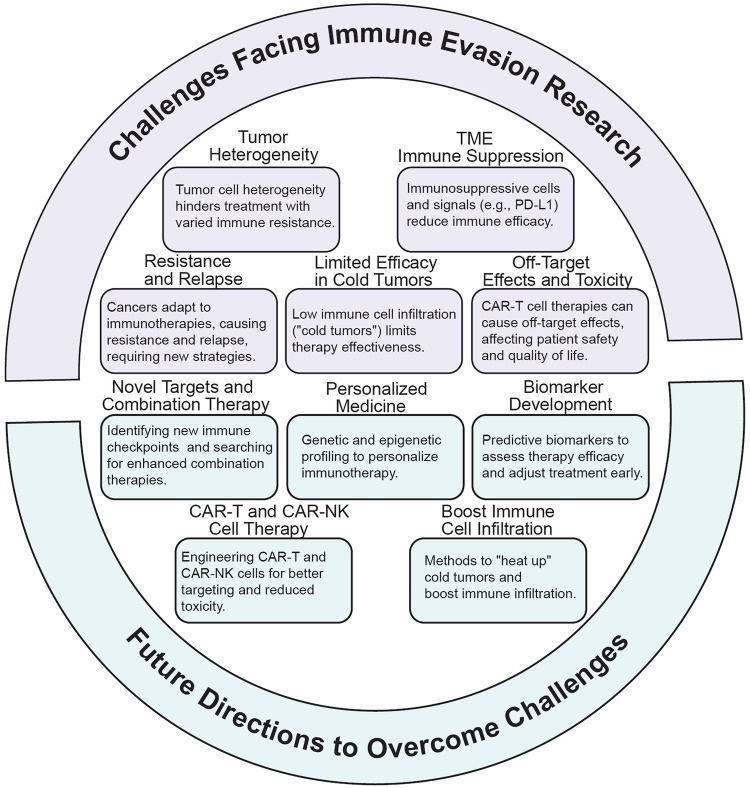
Challenges and future directions in overcoming immune evasion in cancer. This figure outlines the key challenges and future directions in addressing immune evasion in cancer. The “Challenges” section highlights issues such as tumor heterogeneity, where spatial and temporal variations in immune resistance complicate treatment; immune suppression within the TME, driven by cells like Tregs and MDSCs and inhibitory signals like PD-L1 expression; treatment resistance and relapse, which require novel strategies for sustained responses; the limited efficacy of immunotherapies in “cold tumors” with low immune cell infiltration; and off-target effects and toxicity associated with immune-based therapies like CAR-T cells. The “Future Directions” section focuses on potential solutions, including the identification of novel therapeutic targets such as new immune checkpoints and tumor antigens, combination therapies that integrate immune checkpoint inhibitors with other treatments, personalized medicine approaches based on genetic and epigenetic profiling, the development of predictive biomarkers for early assessment of therapy efficacy and resistance, advancements in CAR-T and CAR-NK cell therapies to enhance targeting and reduce toxicity, and strategies to increase immune cell infiltration in cold tumors, such as oncolytic viruses or local modifications of the TME

This section examines the obstacles in overcoming cancer’s adaptive strategies to evade immune detection, highlighting the need for innovative approaches to enhance therapeutic effectiveness (Fig. 9). Resistance to immunotherapy remains one of the most significant challenges in cancer treatment, with many tumors developing mechanisms to evade immune detection and destruction. This resistance can be categorized as primary, adaptive, or acquired. Primary resistance occurs when patients do not respond to initial immunotherapy, often due to insufficient tumor neoantigens or the TME.^520,521^ Adaptive resistance involves changes in the TME or immune system after initial therapy, allowing tumors to evade ongoing immune surveillance.^522^ Acquired resistance, on the other hand, occurs in patients who initially respond but later experience tumor progression, potentially due to new mutations or the loss of target antigens.^523^ Recent studies have also highlighted the role of TME factors in promoting resistance to immunotherapy. These factors may induce the expression of immune checkpoint inhibitors or alter the function of immune cells within the TME. For example, in melanoma, exercise was shown to alleviate tumor hypoxia, enhancing the immune microenvironment by increasing cytotoxic T cells and decreasing regulatory T cells, which improved the efficacy of PD-1/PD-L1 blockade.^524^ Similarly, in esophageal adenocarcinoma (OAC), hypoxia and nutrient deprivation upregulated immune checkpoint molecules on T cells, promoting an immune-resistant phenotype.^525^ Addressing these challenges necessitates a thorough understanding of the underlying mechanisms, which include the activation of immune checkpoint molecules, the release of immunosuppressive cytokines, and the recruitment of Tregs and MDSCs to the TME. However, the current limitations in targeting these mechanisms stem from the complexity and heterogeneity of the TME, which can vary greatly across different patients and tumor types. For instance, the varying expression of PD-L1 across different stages of cancer progression makes it difficult to predict which patients will benefit from PD-1/PD-L1 blockade. Combination therapy targeting multiple immune evasion mechanisms, as well as new drugs capable of reversing resistance, are critical in overcoming these hurdles. For example, a classical immunotherapy combination involving a PD-1 inhibitor and a CTLA-4 inhibitor has been approved for the treatment of various cancers. This suggests that blocking multiple immune checkpoints simultaneously could help overcome resistance. The increased variety of immune checkpoints often contributes to treatment resistance, but dual or even multiple checkpoint blockade has the potential to address this challenge.^526,527^ By incorporating data from diverse omics layers, it may be possible to identify novel biomarkers for early prediction of resistance, guide personalized therapeutic strategies, and develop more effective combination therapies. Furthermore, investigating the molecular interactions between immune checkpoint inhibitors and other therapies could provide insights into how to counteract immune evasion more effectively. The need to improve our understanding of how the immune system adapts to different types of cancers and how resistance develops over time is essential for the design of therapies that can maintain long-term effectiveness.

Identifying reliable biomarkers to predict responses to immunotherapy is crucial for personalizing treatment and optimizing therapeutic outcomes. Current biomarkers, such as PD-L1 expression and tumor mutational burden, provide some predictive value but are not universally applicable across all tumor types or patients.^528^ The complexity of tumors and the dynamic nature of the immune response make it challenging to find biomarkers that consistently predict who will benefit from immunotherapy. Recent research has focused on exploring additional biomarkers, including gene expression profiles,^529^ immune cell composition within the TME, and specific immune cell subtypes like exhausted T cells.^530^ Advances in single-cell sequencing have enabled the identification of novel immune markers at a detailed level, providing deeper insights into the immune landscape of individual tumors.^531^ However, one of the significant limitations of current biomarker strategies is the lack of standardization across platforms and patient populations, which affects their reproducibility and clinical utility. Furthermore, tumor heterogeneity within the same patient and between patients complicates the identification of universal biomarkers. Additionally, circulating biomarkers, such as cell-free DNA (cfDNA),^532^ and CTCs,^533^ are being investigated for their potential to offer real-time monitoring of treatment response and resistance, potentially leading to more tailored and effective treatment strategies.

The variability in patients’ responses to immunotherapy highlights the need for personalized treatment strategies.^534^ Personalized immunotherapy aims to tailor treatment based on each patient’s unique genetic, molecular, and immune characteristics.^534,535^ This approach involves using genomic and transcriptomic data to identify specific mutations, neoantigens, and immune signatures that can be targeted with customized therapies. A promising area in personalized immunotherapy is the development of neoantigen-based vaccines, designed to elicit an immune response against tumor-specific antigens unique to each patient.^535,536^ However, several challenges remain in the widespread application of personalized immunotherapy. One major challenge is the identification of tumor-specific neoantigens that are both immunogenic and present across all tumor cells, as mutations in neoantigens may not always be accessible to the immune system. Furthermore, the heterogeneous nature of tumors can lead to the evolution of new neoantigens over time, which complicates the targeting process.^537^ Additionally, adoptive cell therapies, such as CAR-T cell therapy, can be personalized by engineering T cells to recognize patient-specific tumor antigens. The integration of artificial intelligence and machine learning into personalized immunotherapy is also being explored, with the potential to predict treatment responses and optimize therapeutic regimens based on individual patient data, offering a more precise and effective approach to cancer treatment.

Integrating multi-omics approaches, genomics, transcriptomics, proteomics, and metabolomics is key to understanding immune evasion and optimizing immunotherapy. Genomic and transcriptomic data reveal mutations and gene expression changes, while proteomics uncovers protein interactions and metabolomics highlights metabolic shifts in the TME. One challenge, however, is the complexity and heterogeneity of tumor samples, which can lead to variability in omics data.^538,539^ This necessitates the development of more robust analytical techniques and platforms to ensure the reproducibility and accuracy of multi-omics data across different tumor types and patient populations.^540,541^ By combining these insights, researchers can create a comprehensive model of tumor-immune dynamics, guiding the development of more effective therapies. Furthermore, the integration of multi-omics data can help identify key regulatory nodes in the TME, such as metabolic pathways or signaling networks, that may be responsible for immune evasion. These findings could reveal new therapeutic targets or combination strategies that can overcome the immune resistance mechanisms present in various tumor types.^542^ This holistic approach paves the way for overcoming resistance, identifying novel targets, and improving the precision of cancer immunotherapy. In the future, real-time monitoring of multi-omics data could also offer valuable insights into how tumors evolve during treatment and how immune resistance develops, enabling clinicians to adjust therapy strategies in a more timely and personalized manner.

## Conclusion

In conclusion, the complex and multifaceted mechanisms of immune evasion in cancer represent a significant challenge in oncology, necessitating a comprehensive understanding of the underlying biological processes. Tumor-induced immune suppression, immune checkpoint regulation, and modulation of the TME are central to how cancers escape immune surveillance. Additionally, the influence of genetic and epigenetic modifications, coupled with tumor heterogeneity, further complicates the immune landscape, leading to differential responses to therapies and contributing to resistance. The signaling pathways, such as PD-1/PD-L1, CTLA-4, TGF-β, IL-10, NF-κB, and cGAS-STING, play critical roles in promoting immune evasion, highlighting the importance of targeting these pathways in therapeutic strategies. Current approaches, including immune checkpoint inhibitors, CAR-T cell therapy, cancer vaccines, and emerging modalities like oncolytic viruses and bispecific antibodies, demonstrate the potential to overcome immune resistance. However, challenges such as the development of resistance, the need for predictive biomarkers, and the personalization of immunotherapy underscore the complexities involved in effectively targeting immune evasion. Future directions emphasize integrating multi-omics approaches and novel technologies like nanotechnology to refine and enhance therapeutic outcomes. As research progresses, the ongoing exploration of immune evasion mechanisms and therapeutic innovations promises to advance cancer treatment, aiming to achieve more durable and personalized responses in patients.
